# Ketones as directing groups in photocatalytic sp^3^ C–H fluorination†
†Electronic supplementary information (ESI) available. CCDC 1556373, 1556374 and 1556555. For ESI and crystallographic data in CIF or other electronic format see DOI: 10.1039/c7sc02703f



**DOI:** 10.1039/c7sc02703f

**Published:** 2017-08-11

**Authors:** Desta Doro Bume, Cody Ross Pitts, Fereshte Ghorbani, Stefan Andrew Harry, Joseph N. Capilato, Maxime A. Siegler, Thomas Lectka

**Affiliations:** a Department of Chemistry , Johns Hopkins University , 3400 N. Charles St. , Baltimore , MD 21218 , USA . Email: lectka@jhu.edu

## Abstract

The ubiquitous ketone carbonyl group generally deactivates substrates toward radical-based fluorinations, especially sites closest to it. Herein, ketones are used instead to direct aliphatic fluorination using Selectfluor, catalytic benzil, and visible light. Selective β- and γ-fluorination are demonstrated on rigid mono-, di-, tri-, and tetracyclic (steroidal) substrates employing both cyclic and exocyclic aliphatic ketones as directing groups.

## Abstract 2

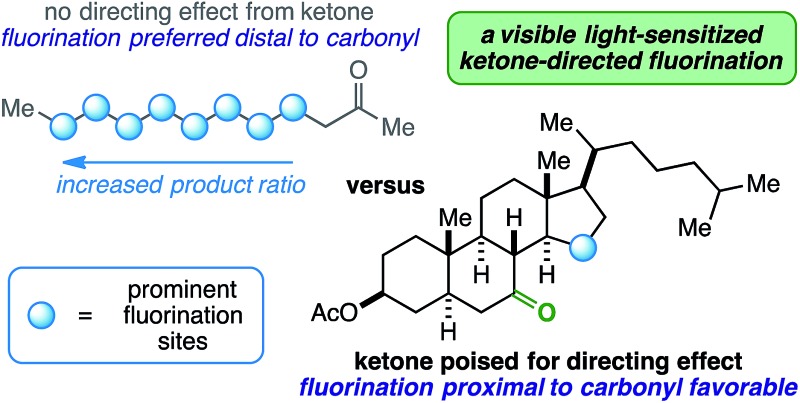
Visible light-sensitization allows conformationally rigid ketones to act as “directing groups” for aliphatic fluorination using Selectfluor, catalytic benzil, and LEDs.

## 

Innate selectivity in aliphatic C–H bond fluorination is achieved when most other C–H bonds are either sterically hindered or electronically deactivated. These factors allow very little control and versatility with respect to radical fluorination of intricate substrates, especially at sites near electron-withdrawing groups such as ketones. Consider the fluorination of 2-dodecanone (Fig. 1). Using existing methods, the reaction will result in a complicated mixture of fluorinated isomers with the relative product ratio increasing the farther the site is from the ketone – a well-documented manifestation of the “polar effect”.^1^ What if the desired site of fluorination is in proximity to the carbonyl (beyond the α-position accessible through enolate chemistry^2^)? Under the right conditions, it is possible that the role of a ketone can be switched from a deactivator to an activator (*i.e.* directing group) on rigid molecular skeletons where the ketone oxygen atom is properly poised.^3^ Herein, we report the ability of ketones to function as directing groups under visible light-sensitized fluorination conditions, thus allowing greater control over regioselectivity in radical-based fluorination.

**Fig. 1:**
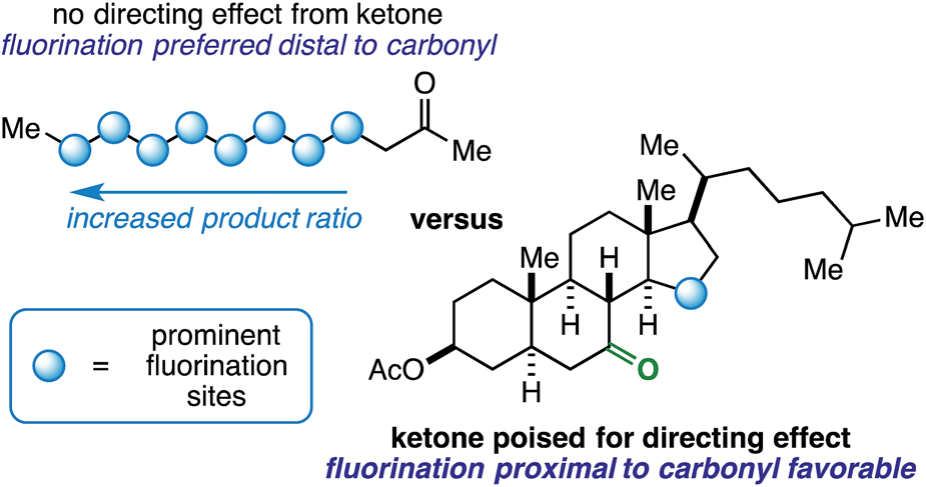
Linear aliphatic ketones *versus* rigid ketones poised for directing effect.

Considering the prominent role of fluorine in medicinal chemistry,^4^ surprisingly few directed sp^3^ C–H fluorination reactions have been developed beyond extant benzylic^5^ or allylic fluorination methods.^6^ Several aliphatic fluorination methods have been reported recently using transition metal catalysts,^7^ radical initiators,^8^ organic molecule catalysts,^9^ and photosensitizers,^10^ but these methods generally are geared toward small, symmetrical molecules or those with more activated or accessible C–H bonds. With respect to more biologically relevant molecules, selective β-fluorination of amino acid derivatives has been achieved through palladium catalysis using a chelating auxiliary ligand in a three-step ligand installation-fluorination-ligand removal process.^11–13^ In our laboratory, we have recently developed an enone-directed photochemical fluorination of polycyclic terpenoid derivatives through direct 300 nm photolysis.^14^ Unfortunately, under the same reaction conditions (using ultraviolet light), we found that ketones afford highly unselective fluorination and are not optimal directing groups; thus, a different approach was necessary.

We imagined that a milder procedure that employs visible light sensitization could allow the necessary balance between reactivity and selectivity to bring the more general and important concept of a ketone-directed reaction to fruition (Fig. 2). Accordingly, we report a visible light-sensitized ketone-directed C–H fluorination method using catalytic benzil (10 mol%), Selectfluor (as a putative atomic source of fluorine^15^), and cool white LED's.^16^ Under these mild conditions, predictably selective β- or γ-fluorination can be achieved based on proximity of the hydrogen atom to the ketone. Both cyclic and exocyclic ketones are demonstrated to direct fluorination effectively on a variety of mono-, di-, tri-, and tetracyclic systems (such as steroidal ketones) in up to 85% yield. In accord with most excited-state ketone hydrogen atom transfer (HAT) chemistry, we found that structural rigidity plays an important role in attaining both desired reactivity and selectivity.^17^ However, we report initial findings that an electron transfer mechanism (either concerted PCET or stepwise ET/PT) is more likely operative.

**Fig. 2:**
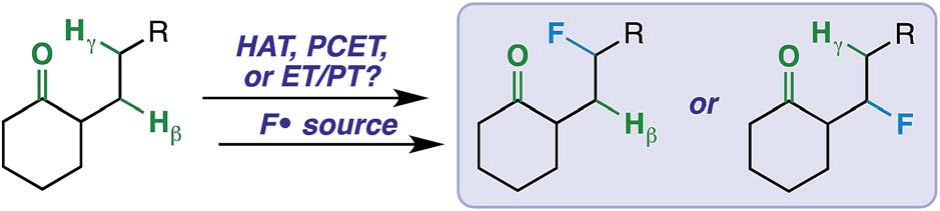
Possible designs for a ketone-directed aliphatic fluorination.

In order to establish an optimal photosensitizer, we began by screening a variety of compounds with a steroidal ketone test substrate (**1**) poised for γ-hydrogen atom transfer, Selectfluor, and a cool white LED source. Note that the LED source, with a sharp absorbance cut-off at *ca.* 400 nm by UV-vis analysis (see ESI†), was used instead of a compact fluorescent light (CFL) source, as the latter has a minor absorbance in the ultraviolet region. Accordingly, we focused primarily on putative sensitizers that possess absorbances above 400 nm; this measure was taken to avoid undesirable reactivity from direct excitation of the substrate and/or fluorine source (corroborated by control experiments that show no reaction in the absence of a sensitizer or light). Although a number of compounds effected the fluorination reaction to form **2** (Table 1), we found the overall best results (82% yield) using a catalytic amount of benzil – a well-established triplet sensitizer that is commercially available, extremely cost-effective, and easy to handle.^18,19^

**Table 1:** Screening for visible light sensitizers

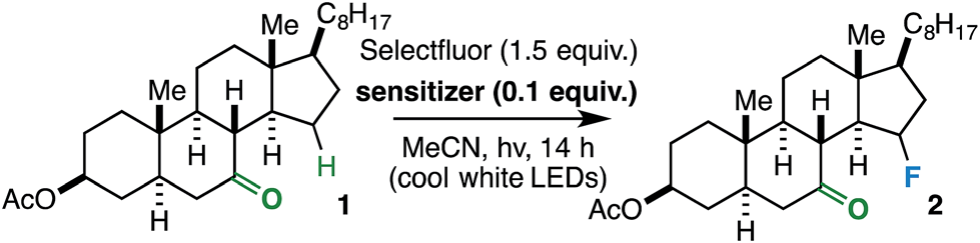		
Entry	Sensitizer	^19^F NMR yield (%)
1	—	0
2	Benzophenone	Trace
3	9-Fluorenone	Trace
4	Xanthone	Trace
5	9,10-Phenanthrenequinone	54
6	Benzil	73^*a*^
**7**	**Benzil**	**82**

^*a*^Reaction with 2.0 equiv. Selectfluor.

It is important to note that the use of other N–F reagents as putative sources of atomic fluorine, *i.e.* NFSI and *N*-fluoropyridinium tetrafluoroborate, do not result in the desired fluorinated product **2**. Although NFSI can also react with alkyl radicals, Selectfluor has been shown to react at a faster rate and may be more likely to participate in electron transfer processes (discussed below).^15a,24^ Additionally, no fluorination reaction was observed upon stirring all three components in the dark at room temperature or running the photochemical reaction under ambient air. Heating the reaction mixture to reflux in the dark also did not afford **2**, but trace unidentified tertiary fluorides were observed in the ^19^F NMR spectrum of the crude reaction mixture. Finally, a slight decrease in product yield was observed when using Selectfluor in greater than 1.5 equiv. (Table 1, entry 6); this is a function of a decrease in selectivity, as greater quantities of other fluorinated isomers were observed by ^19^F NMR analysis of the crude reaction mixture.

With an optimized protocol in hand, we focused our efforts on evaluation of the substrate scope with respect to a variety of common ring systems (Table 2). Menthone contains two tertiary carbon sites, but we observe strictly compound **3** in 55% yield under fluorination conditions, consistent with the notion of ketone involvement (note that although a putative 6-membered transition state from one of the methyl groups can be imagined, we did not observe primary fluorides). Compounds **4** and **5** represent examples of benzylic fluorination through putative 5-membered transition states. It is important to note that ethylbenzene does not undergo benzylic fluorination under the same conditions, suggesting the ketone plays a necessary role. In addition, compound **4** demonstrates reaction compatibility with a boron-based functional group (pinacolborane) that is used widely in cross-coupling applications.^20^

**Table 2:** Substrate scope: mono-, di-, tri-, and exocyclic ketone directing groups for fluorination of cyclic and exocyclic sp^3^ C–H sites^*a*^

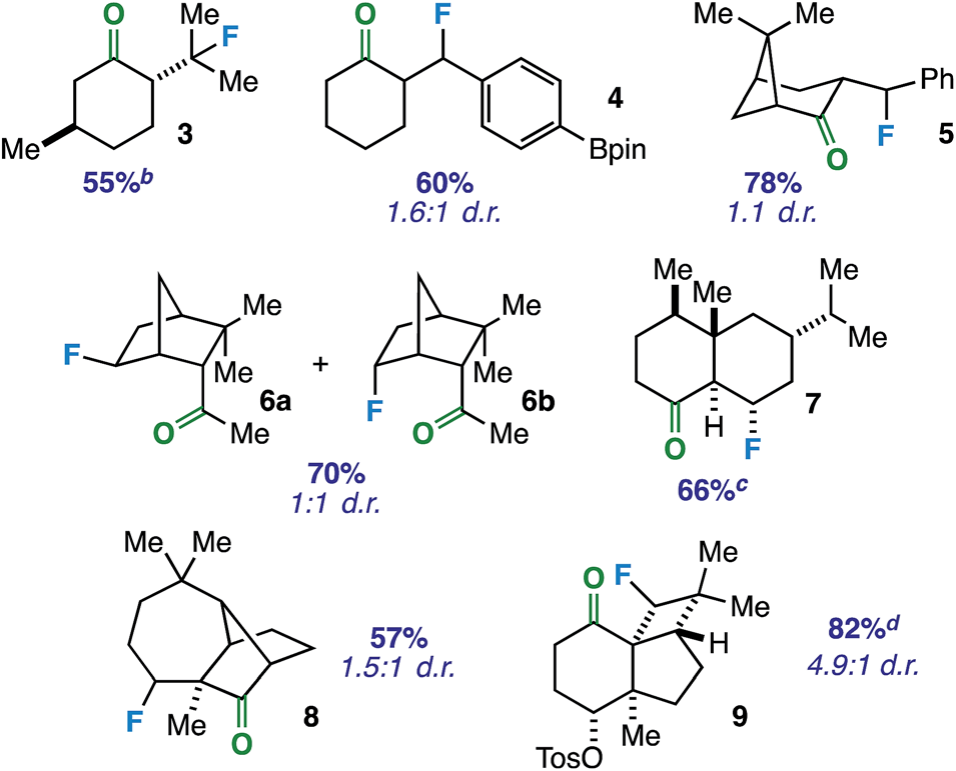

^*a*^Unless otherwise specified, all reactions were stirred in MeCN with Selectfluor (1.5 equiv.) and benzil (10 mol%) and irradiated with cool white LED's for 14 h. Yields include both diastereomers and were determined by integration of ^19^F NMR signals relative to an internal standard and confirmed by isolation of products through column chromatography on silica gel. Major diastereomer (with respect to C–F bond) depicted where known.

^*b*^ ^19^F NMR yield.

^*c*^Diastereomeric ratio (d.r) not determined.

^*d*^Yield based on recovered starting material.

In these instances, the tertiary and benzylic C–H sites are arguably more activated toward fluorination. Thus, we examined substrates that should target specific secondary carbon sites. Employing an exocyclic ketone on a rigid norbornane scaffold, we were able to access a mixture of *exo* and *endo* fluorides (**6**) at the predicted site in 70% yield. Beyond bridged bicyclic systems, there are also opportunities for ketone-directed fluorination on certain decalone cores. For instance, compound **7** (derived from sesquiterpenoid valencene) was formed selectively in the presence of other tertiary carbon sites distal from the ketone. Subsequently, we examined directed fluorination on more complex tricyclic ring systems. For one, a longifolene-derived ketone provided selective fluorination of the most accessible carbon site on the cycloheptane ring (**8**). Remarkably, we were also able to target a C–H bond on a strained cyclobutane ring to form fluorinated kobusone derivative **9**. What is more, this reaction proceeded smoothly in the presence of an oxidized sulfur-containing functional group (*i.e.* a tosylate).

Considering the prevalence and importance of biologically active steroidal ketones,^21^ we surveyed the fluorination of ketones akin to cholesterol derivative **2** (Table 3). Compounds **10–12** represent cholesterol, testosterone, and progesterone derivatives with starting ketones at C7 also poised for C15 functionalization. Note that compound **10** also exhibits reaction tolerance of aliphatic chlorides. Selective γ-fluorination was observed in each case (62–85% yield).

**Table 3:** Substrate scope: steroidal ketone directing groups for predictable γ- or β-fluorination of sp^3^ C–H sites^*a*^

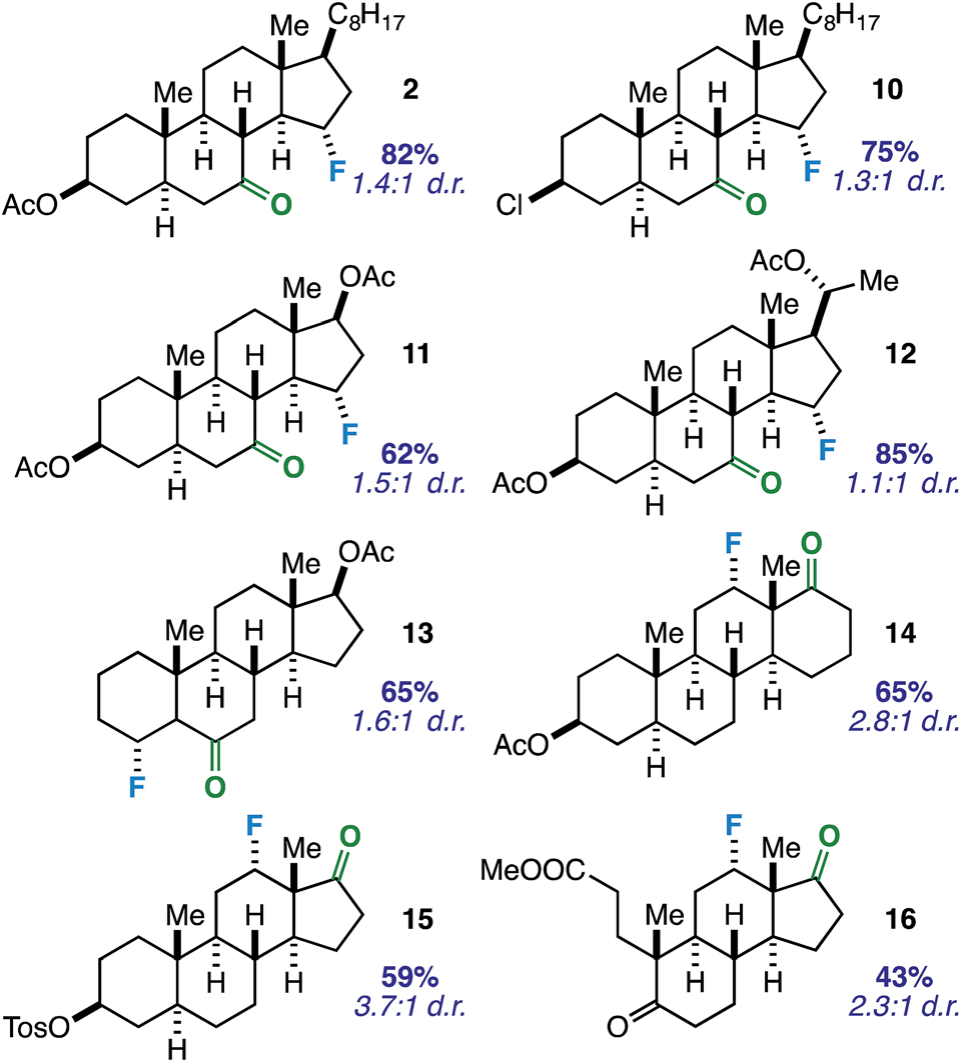

^*a*^Unless otherwise specified, all reactions were stirred in MeCN with Selectfluor (1.5 equiv.) and benzil (10 mol%) and irradiated with cool white LED's for 14 h. Yields include both diastereomers and were determined by integration of ^19^F NMR signals relative to an internal standard and confirmed by isolation of products through column chromatography on silica gel. Major diastereomer (with respect to C–F bond) depicted where known.

Subsequently, we applied the ketone-directed reaction to β-fluorination on the steroid core. Thus, a C6 steroidal ketone was found to fluorinate the C4 position through a putative 5-membered transition state to afford **13** in 65% yield. No evidence of degradation to the corresponding enone was observed following column chromatography on silica gel. In another instance, C12-fluorinated *trans*-androsterone derivative **14** (with an expanded D-ring) was also readily accessible. Recognizing that fluorinated *trans*-androsterone derivatives may be more desirable with the cyclopentane ring intact, we asked: will the cyclopentanone also access C12 fluorination through a 5-membered transition state? To our satisfaction, compound **15** was formed in 59% yield. We also examined a tricyclic secosteroid substrate (**16**) as another example of a cyclopentanone moiety directing fluorination to the adjacent cyclohexane ring.

Importantly, note that the virtue of the tetra- and tricyclic ring systems discussed thus far is their decreased conformational flexibility; this allows for selective, predictable fluorination in a somewhat paradoxical manner. That is, more complex polycyclic carbon frameworks, in general, promote selective C–H fluorination where it intuitively may inhibit it in other non-directed circumstances. Thus, this method appears to be best suited for late-stage fluorination of larger, more intricate structures.^22^

On another note, the ideal substrates for this reaction have a clear distinction over the preference for γ- *vs.* β-fluorination based on geometric constraints. However, how does the reaction proceed when both 5- and 6-membered transition states are possible? Progesterone, with an acetyl group at C17, can act as a probe and also provide a real-world example of when this competitive fluorination could be of interest (*i.e.* to access different fluorinated bioactive steroids). Accordingly, we found that the free rotation of the σ-bond between C17 and C20 allows fluorination of both C12 (**17**) and C16 (**18**) in a ratio of 1.0 : 3.1 (55% total yield, Scheme 1). Although the regioselectivity is modest, this may be an asset in a medicinal chemistry setting where multiple fluorinated regioisomers of similar steroids are desirable for biological testing.^23^

**Scheme 1:**
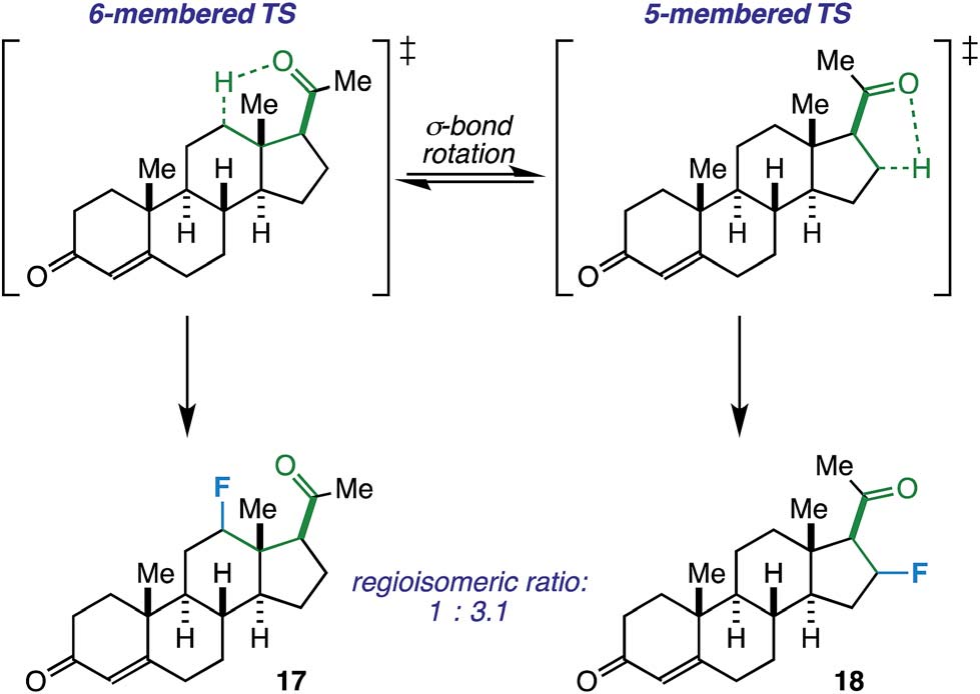
Fluorinated progesterone product ratio from putative 5- *vs.* 6-membered transition states.

At this point, we have demonstrated cyclic (5- and 6-membered rings) and exocyclic aliphatic ketones directing fluorination on either cyclic (4-, 5-, 6-, and 7-membered rings) or short, linear side-chain sites. How does the reaction hold up to linear aliphatic ketones? Using 2-heptanone as the substrate, we observed δ-, γ-, and β-fluorination in 2.3 : 1.3 : 1.0, respectively, in the ^19^F NMR spectrum of the crude reaction mixture. This could indicate an indiscriminate radical chain mechanism instead of a directed reaction,^24,25^ as it exhibits features of the so-called polar effect.^1^ In order to expand on this result, we also ran the reaction with 2-decanone and 2-dodecanone. In each case, there was a large preference for fluorination at the penultimate carbon atom alongside multiple secondary fluoride isomers (Fig. 1).

Thus, under the same reaction conditions, the rigid ketones afford selective β- or γ-fluorination and the conformationally flexible ketones do not. Perhaps the linear ketones (1) prefer intermolecular over intramolecular HAT and/or (2) promote cage escape of the *N*-centered radical derived from Selectfluor that is a key player in radical chain mechanisms.^24,25^ Accordingly, we ran the reactions with the linear ketones under more dilute conditions to favor intramolecular HAT,^26^ but observed the same product distributions by ^19^F NMR. What is more, a HAT mechanism directed by a ketone would imply accessibility of the ketone triplet excited state. The reported triplet energy of benzil (∼53 kcal mol^–1^),^27^ which is the only chromophore present under our conditions, is not high enough to undergo triplet–triplet energy transfer with aliphatic ketones^28^ (typically with triplet energies of ∼80 kcal mol^–1^).^29^ Therefore, the ketone triplet state should not be present in any significant concentration, and a HAT mechanism seems unlikely for both flexible and rigid ketones.^30^

Conceivably, the benzil triplet state can promote the reaction instead by facilitating electron transfer from the substrate to Selectfluor;^31,32^ this would result in formation of the well-established *N*-centered radical intermediate. As alternative ways to generate this intermediate, we subjected the linear ketones to our established copper(i)/Selectfluor^7*a*^ and BEt_3_/Selectfluor^8^ protocols and found nearly identical fluorinated product distributions in each case. Interestingly, when representative rigid cyclic ketones (*e.g.* starting ketones for compounds **2** and **11**) were also subjected to the BEt_3_/Selectfluor protocol (in absence of light and a sensitizer), the same selectivity was observed as the visible light-sensitized reaction (Scheme 2). Thus, this putative *N*-centered radical intermediate is likely the key player in the mechanism for both flexible and rigid ketones. As this intermediate is known to be a powerful oxidant, it is possible that an electron transfer (ET) mechanism is operative whereby the ketone assists in proton transfer (PT) instead of HAT.^33^ An electron transfer mechanism is also consistent with our observation that the reaction is best suited for our relatively large substrates (with relatively low ionization potentials). Additionally, if the ketone is not properly poised to act as the intramolecular “base,” then it is possible other reaction components could act as intermolecular bases (MeCN, the amine derived from Selectfluor, *etc.*), which can explain the loss of selectivity in conformationally flexible ketones *versus* rigid ketones. Lastly, at this time, it is unclear whether the mechanism is concerted (proton-coupled electron transfer, or PCET) or stepwise (electron transfer/proton transfer, or ET/PT)^34^ and whether it involves a chain propagation or a closed cycle; we will explore these aspects in future studies.

**Scheme 2:**
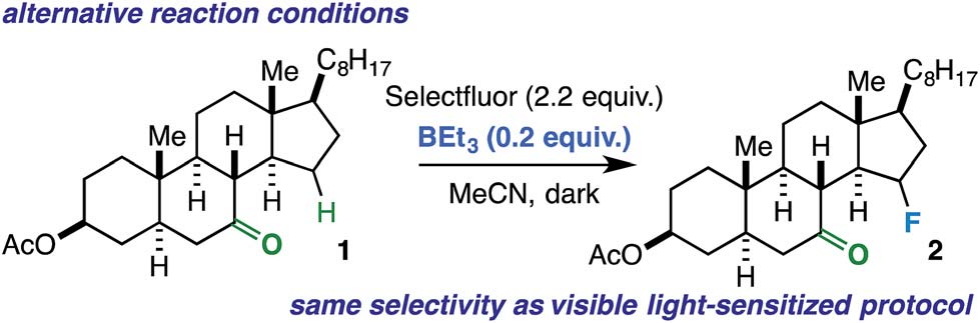
Same selectivity observed using BEt_3_/Selectfluor protocol as an alternative way to generate the *N*-centered radical intermediate from Selectfluor in the absence of light and benzil.

## Conclusions

In summary, this visible light sensitization approach creates an opportunity to use ubiquitous ketones as directing groups in photochemical sp^3^ C–H fluorination. In a somewhat paradoxical manner, the method is best suited for complex, polycyclic molecules (likely due to increased conformational rigidity); however, its utility as a directed reaction is also demonstrated to be more general. It allows easy access to fluorinated products that have not been synthesized previously in good yields and selectivity, and it represents a necessary leap forward in directing radical fluorination. Future studies will seek to elucidate the reaction mechanism by exploring the nature of putative electron transfer processes.

## Conflicts of interest

There are no conflicts to declare.

## Supplementary Material

Supplementary information

Crystal structure data

## References

[cit1] (a) Walling C., Free Radicals in Solution, Wiley, New York, NY, 1957.

[cit2] Paull D. H., Scerba M. T., Alden-Danforth E., Widger L. R., Lectka T. (2008). J. Am. Chem. Soc..

[cit3] Ariel S., Ramamurthy V., Scheffer J. R., Trotter J. (1983). J. Am. Chem. Soc..

[cit4] Purser S., Moore P. R., Swallow S., Gouverneur V. (2008). Chem. Soc. Rev..

[cit5] Bloom S., Pitts C. R., Woltornist R., Griswold A., Holl M. G., Lectka T. (2013). Org. Lett..

[cit6] Braun M.-G., Doyle A. (2013). J. Am. Chem. Soc..

[cit7] Bloom S., Pitts C. R., Miller D., Haselton N., Holl M. G., Urheim E., Lectka T. (2012). Angew. Chem., Int. Ed..

[cit8] Pitts C. R., Ling B., Woltornist R., Liu R., Lectka T. (2014). J. Org. Chem..

[cit9] Amaoka Y., Nagamoto M., Inoue M. (2013). Org. Lett..

[cit10] Bloom S., Knippel J. L., Lectka T. (2014). Chem. Sci..

[cit11] Hull K. L., Anani W. Q., Sanford M. S. (2006). J. Am. Chem. Soc..

[cit12] Zhu R.-Y., Tanaka K., Li G.-C., He J., Fu H.-Y., Li S.-H., Yu J.-Q. (2015). J. Am. Chem. Soc..

[cit13] Li Z., Song L., Li C. (2013). J. Am. Chem. Soc..

[cit14] Pitts C. R., Bume D. D., Harry S. A., Siegler M. A., Lectka T. (2017). J. Am. Chem. Soc..

[cit15] Rueda-Becerril M., Sazepin C. C., Leung J. C. T., Okbinoglu T., Kennepohl P., Paquin J.-F., Sammis G. M. (2012). J. Am. Chem. Soc..

[cit16] Zhao J., Wu W., Sun J., Guo S. (2013). Chem. Soc. Rev..

[cit17] Wagner P. and Park B.-S., in Organic Photochemistry, ed. A. Padwa, Marcel Dekker, Inc., New York, NY, 1991, vol. 11, ch. 4, pp. 227–366.

[cit18] Herkstroeter W. G., Lamola A. A., Hammond G. S. (1964). J. Am. Chem. Soc..

[cit19] Both benzil and 9,10-phenanthrenequinone have more significant absorptions in the LED emission region (>400 nm) than benzophenone, 9-fluorenone, and xanthone. Such overlap is key to their success (and a testament to their role) as photosensitizers in this reaction

[cit20] Miyaura N., Suzuki A. (1995). Chem. Rev..

[cit21] Michaudel Q., Journot G., Regueiro-Ren A., Goswami A., Guo Z., Tully T. P., Zou L., Ramabhadran R. O., Houk K. N., Baran P. S. (2014). Angew. Chem., Int. Ed..

[cit22] Neumann C. N., Ritter T. (2015). Angew. Chem., Int. Ed..

[cit23] In medicinal chemistry, a “fluorine scan” is common practice in drug derivatization and for studying SAR's. Thus, access to different regioisomers may be desirable. See: Gillis E. P. Eastman K. J. Hill M. D. Donnelly D. J. Meanwell N. A., J. Med. Chem., 2015, 58 , 8315 –8359 . 26200936 10.1021/acs.jmedchem.5b00258

[cit24] Pitts C. R., Bloom S., Woltornist R., Auvenshine D. J., Ryzhkov L. R., Siegler M. A., Lectka T. (2014). J. Am. Chem. Soc..

[cit25] Pitts C. R., Ling B., Snyder J. A., Bragg A. E., Lectka T. (2016). J. Am. Chem. Soc..

[cit26] Reactions were performed at 2-fold, 4-fold, and 8-fold dilutions from our standard concentration

[cit27] Evans T. R., Leermakers P. E. (1967). J. Am. Chem. Soc..

[cit28] Turro N. J., in Modern Molecular Photochemistry, The Benjamin/Cummings Publishing Company, Inc., Menlo Park, CA, 1978, ch. 9, pp. 296–361.

[cit29] Nau W. M., Scaiano J. C. (1996). J. Phys. Chem..

[cit30] In addition, one would expect to observe byproducts from Norrish II cleavage in linear and certain exocyclic ketones if the reaction goes through a HAT mechanism – such byproducts were not observed in any significant amount

[cit31] Electron transfer may occur directly from the substrate to the benzil triplet state or to a benzil/Selectfluor exciplex. See: Kee J. W. Shao H. Kee C. W. Lu Y. Soo H. S. Tan C.-H., Catal. Sci. Technol., 2017, 7 , 848 –857 .

[cit32] Griesbeck A. G., Hoffmann N., Warzecha K.-D. (2007). Acc. Chem. Res..

[cit33] Hsieh C.-C., Jiang C.-M., Chou P.-T. (2010). Acc. Chem. Res..

[cit34] Warren J. J., Tronic T. A., Mayer J. M. (2010). Chem. Rev..

