# Supplementary material for: Ketones as directing groups in photocatalytic sp3 C–H fluorination
Source: Chem Sci. 2017 Aug 11;8(10):6918–23. doi: 10.1039/c7sc02703f (PMC5637129; doi:10.1039/c7sc02703f)
Supplement: Supplementary file 1 [file SC-008-C7SC02703F-s001.pdf]

Electronic Supplementary Material (ESI) for Chemical Science.
This journal is © The Royal Society of Chemistry 2017

Supporting Information

Ketones as Directing Groups in Photocatalytic sp^3 C-H Fluorination

Desta Doro Bume,† Cody Ross Pitts,† Fereshte Ghorbani, Stefan Andrew Harry, Joseph N. Capilato, Maxime A. Siegler, Thomas Lectka*

Department of Chemistry, Johns Hopkins University, 3400 N. Charles St., Baltimore, MD, 21218, United States

Contents:

General Information, S2
General Fluorination Procedure, S2
Syntheses of Starting Materials, S3-S14
Fluorinated Product Characterization Data, S15-S24
NMR Spectral Data of Fluorinated Products, S25-S75
NMR Spectral Data of Starting Materials, S76-S103
UV-vis Spectrum of Cool White LED Light Source, S104
UV-vis Spectra of Photosensitizers, S105-109
X-ray Crystallographic Data, S110-S120
References, S121

General Information

Unless otherwise stated, all reactions were carried out under strictly anhydrous conditions and N_2 atmosphere. All solvents were dried and distilled by standard methods. All ^{1}H spectra were acquired on a 400 MHz NMR spectrometer in $CDCl_3$, ^{19}F spectra were acquired on a 300 MHz NMR spectrometer in CD_3CN or $CDCl_3$, and ^{13}C NMR spectra were acquired on a 400 MHz NMR spectrometer in $CDCl_3$. The ^{1}H, ^{13}C, and ^{19}F NMR chemical shifts are given in parts per million (δ) with respect to an internal tetramethylsilane (TMS, δ = 0.00 ppm) standard and/or 3-chlorobenzotrifluoride (δ = −64.2 ppm relative to $CFCl_3$).[1] NMR data are reported in the following format: chemical shift (integration, multiplicity (s = singlet, d = doublet, t = triplet, q = quartet, m = multiplet), coupling constants (Hz)). IR data were obtained using an ATR-IR instrument. Spectral data were processed with Bruker software. Photochemical reactions were run in front of a 72-LED work light (Designers Edge L1923). HPLC purification (if necessary) was conducted on a Teledyne Isco CombiFlash EZ Prep system using a Dynamax-60A SiO_2 column and HPLC grade EtOAc and hexanes. The Gaussian '09 package was used for all calculations.[2]

General Fluorination Procedure

Selectfluor (133 mg, 0.38 mmol), benzil (5.0 mg, 0.025 mmol), and the substrate (0.25 mmol) were added to an oven-dried μω vial equipped with a stir bar; the vial was then sealed with a cap w/ septum using a crimper and evacuated/refilled with N_2 multiple times. Anhydrous CH_3CN (4 mL) was added, and the reaction mixture was irradiated with a cool white LED work light while stirring. After 14 h, a 0.3 mL aliquot was taken for ^{19}F NMR yield determination, and the rest of the reaction mixture was transferred to a separatory funnel, diluted with H_2O, and extracted into CH_2Cl_2. The combined organic layers were washed with H_2O and brine, then dried with $MgSO_4$, filtered through Celite, and concentrated. The crude reaction mixture was purified via gradient column chromatography on silica gel eluting with EtOAc:hexanes.

Syntheses of Starting Materials

3β-Acetoxy-5α-cholestan-7-one[3,4]

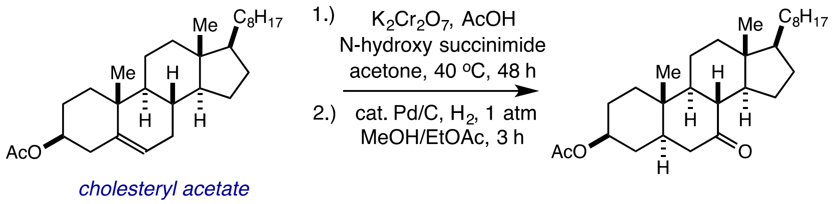

Cholesteryl acetate (4.0 g, 9.3 mmol) was dissolved in a mixture of acetone (475 mL) and acetic acid (50 mL) in a round-bottom flask equipped with a stir bar and reflux condenser under N_2. The reaction mixture was treated with *N*-hydroxysuccinimide (10.7 g, 93.0 mmol) and $K_2Cr_2O_7$ (11.0 g, 37.2 mmol), and then the reaction mixture was stirred at 40 °C for 48 h. The reaction mixture was cooled to rt, quenched with 10% aqueous sodium metabisulfite solution, filtered through Celite, and extracted into Et_2O. The combined organic layers were washed with saturated $NaHCO_3$ and brine, and then dried with $MgSO_4$ and concentrated. The crude residue was recrystallized in MeOH to provide 3β-acetoxy-cholest-5-en-7-one (3.28 g, 80%).

A balloon filled with hydrogen was placed over a round-bottom flask containing a solution of 3β-acetoxy-cholest-5-en-7-one (1.20 g, 2.7 mmol) and 10% Pd/C (147 mg) in MeOH (45 mL) and EtOAc (15 mL). The reaction was then stirred at rt for 3 h. The catalyst was removed by filtration through Celite, and the filtrate was concentrated. The crude residue was recrystallized using MeOH/EtOAc to obtain 3β-acetoxy-5α-cholestan-7-one (1.09 g, 90%).

White solid; m.p. 189-191 ºC. ^{1}H NMR (400 MHz, $CDCl_3$): δ 4.70-4.62 (m, 1H), 2.35-2.29 (m, 2H), 2.22-2.14 (m, 1H), 2.03-1.95 (m, 2H), 2.01 (s, 3H), 1.92-1.83 (m, 2H), 1.77 (dt, *J* = 13.4, 3.5 Hz, 1H), 1.69-1.61 (m, 1H), 1.60-1.46 (m, 6H), 1.42-1.21 (m, 5H), 1.19-0.95 (m, 9H), 1.08 (s, 3H), 0.89 (d, *J* = 6.5 Hz, 3H), 0.85 (d, *J* = 6.7 Hz, 3H), 0.84 (d, *J* = 6.6 Hz, 3H), 0.63 (s, 3H); $^{13}C\{^1H\}$ NMR (100 MHz, $CDCl_3$): δ 211.5, 170.4, 72.8, 55.02, 55.00, 49.96, 48.9, 46.5, 45.9, 42.5, 39.5, 38.7, 36.1, 36.0, 35.8, 35.7, 33.9, 28.4, 28.0, 27.1, 25.0, 23.8, 22.8, 22.6, 21.8, 21.3, 18.8, 12.1, 11.7; υ_{max} (ATR-IR): 1728, 1706 cm^{-1}; λ_{max} (CH_3CN): 293 nm; HRMS (ESI) m/z $C_{29}H_{48}O_3Na^+$: calc 467.349566, observed 467.349739.

2-(4-(4,4,5,5-Tetramethyl-1,3,2-dioxaborolan-2-yl)benzyl)cyclohexan-1-one[5]

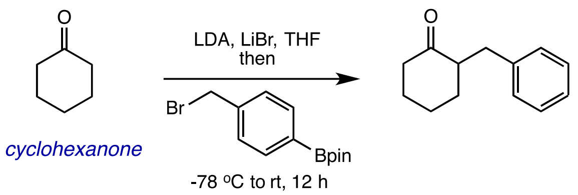

To a flame-dried three-neck round-bottom flask equipped with a stir bar and reflux condenser under N_2 was added LiBr (0.24 g, 3.0 mmol), diisopropylamine (0.45 mL, 3.2

mmol), and THF (15 mL). The reaction mixture was cooled to -20 °C, and then slowly treated with n-BuLi (2.0 mL, 3.2 mmol, 1.6 M in hexanes) and stirred for 30 min. The reaction mixture was cooled to -78 °C, and then cyclohexanone (0.29 mL, 2.8 mmol) was added dropwise and stirred for an additional 30 min. Subsequently, 4-bromomethylphenylboronic acid pinacol ester (1.00 g, 3.4 mmol) dissolved in THF (2.0 mL) was added dropwise, the reaction mixture was slowly warmed to rt, and stirred for 12 h. Then the reaction was quenched with 1.0 M HCl, extracted into Et_2O, the combined organic layers were dried over $MgSO_4$, filtered through Celite, and concentrated. The crude residue was purified via column chromatography on silica gel eluting with EtOAc:hexanes to provide 2-(4-(4,4,5,5-tetramethyl-1,3,2-dioxaborolan-2-yl)benzyl)cyclohexan-1-one (0.79 g, 90 %).

White solid; m.p. 78-80 ºC. ^1H NMR (400 MHz, $CDCl_3$): δ 7.74 (d, J = 8.0 Hz, 2H), 7.18 (d, J = 7.9 Hz, 2H), 3.25 (dd, J = 13.7, 4.6 Hz, 1H), 2.59-2.51 (m, 1H), 2.46-2.39 (m, 2H), 2.35-2.27 (m, 2H), 2.08-1.96 (m, 2H), 1.83-1.77 (m, 1H), 1.72-1.49 (m, 2H), 1.38-1.28 (m, 1H), 1.34 (s, 12H); $^{13}C\{^1H\}$ NMR (100 MHz, $CDCl_3$): δ 211.8, 143.6, 134.6, 128.4, 83.4, 52.1, 41.9, 35.5, 33.2, 27.8, 24.8, 24.7, 24.7; υ_{max} (ATR-IR): 1710 cm^{-1}; λ_{max} (CH_3CN): 296 nm; HRMS (ESI) m/z $C_{19}H_{26}BO_3Na^+$: calc 337.194546, observed 337.194437.

<u>3-Benzylbicyclo[3.1.1]heptan-2-one</u>[5]

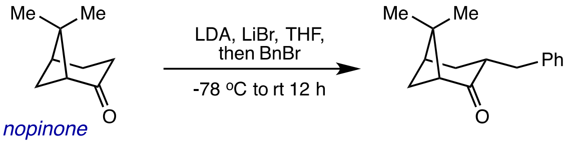

To a flame-dried three-neck round-bottom equipped with a stir bar and reflux condenser under N_2 was added LiBr (0.48 g, 5.5 mmol), diisopropylamine (0.80 mL, 5.7 mmol), and THF (17 mL). The reaction mixture was cooled to -20 °C, and then slowly treated with n-BuLi (3.60 mL, 5.7 mmol, 1.6 M in hexanes) and stirred for 30 min. The reaction mixture was cooled to -78 °C, and then (1R)-(+)-nopinone (0.70 mL, 5.0 mmol) dissolved in THF (2.0 mL) was added dropwise and stirred for an additional 30 min. Subsequently, benzyl bromide (0.90 mL, 7.5 mmol) dissolved in THF (2.0 mL) was added dropwise, the reaction mixture was slowly warmed to rt, and stirred for 12 h. The reaction was then quenched with 1.0 M HCl, extracted into Et_2O, the combined organic layers were dried over $MgSO_4$, filtered through Celite, and concentrated. The crude residue was purified via column chromatography on silica gel eluting with EtOAc:hexanes to provide 3-benzylbicyclo[3.1.1]heptan-2-one (0.91 g, 80 %).

Colorless oil. ^1H NMR (400 MHz, $CDCl_3$): δ 7.34-7.27 (m, 2H), 7.25-7.19 (m, 3H), 3.40-3.35 (m, 1H), 2.78-2.60 (m, 3H), 2.55-2.47 (m, 1H), 2.22-2.16 (m, 1H), 2.01-1.92 (m, 1H), 1.77 (dt, J = 14.1, 3.4 Hz, 1H), 1.31 (d, J = 10.5 Hz, 1H), 1.32 (s, 3H), 0.89 (s, 3H); $^{13}C\{^1H\}$ NMR (100 MHz, $CDCl_3$): δ 214.8, 139.5, 129.1, 128.3, 126.2, 58.2, 45.0, 41.7, 40.9, 40.5, 27.3, 25.7, 25.5, 22.0; υ_{max} (ATR-IR): 1706 cm^{-1}; λ_{max} (CH_3CN): 295 nm; HRMS (ESI) m/z $C_{14}H_{16}ONa^+$: calc 251.141420, observed 251.140246.

Acetylnorcamphane[6]

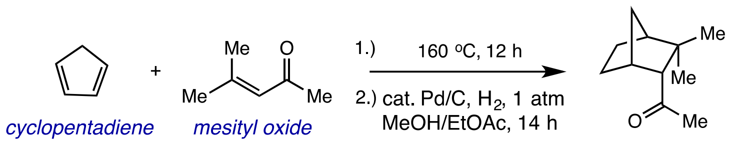

A mixture of cyclopentadiene (1.19 g, 17.8 mmol) and mesityl oxide (8.76 g, 89.0 mmol) was heated to 160 °C in an autoclave for 14 h. The reaction mixture was then slowly cooled to rt, the starting materials were distilled off, and the remaining residue was purified via column chromatography eluting with EtOAc:hexanes to provide 2-acetyl-3,3-dimethylbicyclo[2.2.1]hept-5-ene (0.30 g, 10%).

A balloon filled with hydrogen was placed over a round-bottom flask containing a solution of 2-acetyl-3,3-dimethylbicyclo[2.2.1]hept-5-ene (0.30 g, 1.83 mmol) and 10% Pd/C (73 mg) in MeOH (20 mL) and EtOAc (5 mL). The reaction mixture was then stirred at rt for 14 h. The catalyst was removed by filtration through Celite, and the filtrate was concentrated. The crude residue was purified via column chromatography eluting with EtOAc:hexanes to provide acetylnorcamphane (0.28 g, 92%). The endo- and exo-isomers were purified via HPLC.

Colorless oil. ^{1}H NMR (400 MHz, $CDCl_3$): δ 2.42-2.39 (m, 1H), 2.37-2.35 (m, 1H), 2.06 (s, 3H), 1.94-1.86 (m, 1H), 1.81-1.79 (m, 1H), 1.66-1.56 (m, 2H), 1.40-1.31 (m, 1H), 1.29-1.18 (m, 2H), 1.16 (s, 3H), 0.98 (s, 3H); ^{13}C{^{1}H} NMR (100 MHz, $CDCl_3$): δ 210.3, 63.8, 49.8, 41.3, 38.2, 37.4, 32.4, 31.7, 24.5, 23.1, 21.4; υ_{max} (ATR-IR): 1700 cm^{-1}; λ_{max} (CH_3CN): 291 nm.

Nootkat-1-one[7,8,9]

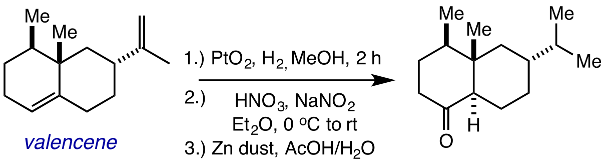

To a flame-dried round-bottom flask equipped with a stir bar was dissolved valencene (3.70 g, 18.0 mmol) and PtO_2 (100 mg, 0.44 mmol) in MeOH. The resulting solution was exposed to H_2 via balloon and stirred for 2 h. The catalyst was then removed by filtration through Celite, the filtrate was concentrated, and the crude residue was purified via column chromatography eluting with EtOAc:hexanes to provide dihydrovalencene (3.34 g, 90%).

The dihydrovalencene (1.00 g, 4.85 mmol) was dissolved in Et_2O (40 mL) using a 250 mL three-neck round-bottom flask equipped with a stir bar and condenser. After cooling the solution to 0 ºC, aqueous 60-70% HNO_3 (25 mL) was added drop wise over 20 min, and the reaction mixture was stirred for additional 5 min. At this point, $NaNO_2$ (0.69 g, 2.0 mmol) was added, and the reaction mixture was slowly warmed to rt over 2 h. The reaction mixture was then transferred to a separatory funnel containing 40 mL of cold water. The aqueous layer was removed without agitation, and then the Et_2O layer was washed with cold H_2O, 1.0 M NaOH, and then H_2O. The combined organic layers were

dried over Na_2SO_4, filtered through Celite, and concentrated. The crude residue (1.00 g) was dissolved in AcOH (40 mL) and diluted with H_2O (4 mL) in a round-bottom flask equipped with a stir bar and a condenser. The resulting reaction mixture was treated with Zn dust in portions over 20 min at rt, and was then heated to reflux for 4 h. Upon cooling to rt, the mixture was diluted with EtOAc, filtered through Celite (to remove residual Zn and related byproducts). The organic layer was washed with H_2O, dried over MgSO4, filtered through Celite, and concentrated. The crude residue was purified via silica gel column chromatography eluting with EtOAc:hexanes to provide nootkat-1-one (0.30 g, 49%).
Colorless oil. ^{1}H NMR (400 MHz, $CDCl_3$): δ 2.42-2.23 (m, 2H), 2.07 (dd, J = 12.2, 3.2 Hz, 1H), 1.88-1.59 (m, 6H), 1.50-1.32 (m, 2H), 1.27-1.14 (m, 1H), 0.89-0.80 (m, 11H), 0.62 (s, 3H); $^{13}C\{^1H\}$ NMR (100 MHz, $CDCl_3$): δ 212.9, 58.0, 42.8, 42.2, 41.8, 41.2, 38.1, 32.8, 31.4, 28.2, 20.6, 19.9, 19.4, 14.4, 12.0; υ_{max} (ATR-IR): 1713 cm^{-1}; λ_{max} (CH_3CN): 292 nm; HRMS (ESI) m/z $C_{15}H_{26}ONa^+$: calc 245.187587, observed 245.187501.

(+)-Longicamphenylone[10,11]

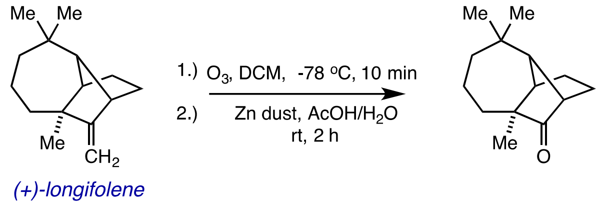

To a flame-dried three-neck round-bottom flask equipped with a stir bar was added (+)-longifolene (mixture of isomers, 1.00 g, 4.9 mmol) and DCM (25 mL). The solution was then cooled to -78 °C, purged with oxygen for 5 min, and then a stream of ozone gas was bubbled through the solution for 10 min (excess ozone was quenched by bubbling through a saturated aqueous $NaSO_3$). Subsequently, the solution was purged with oxygen for 5 min, warmed to rt under N_2, diluted with aqueous 50% AcOH (12 mL), and then treated with a Zn dust (0.50 g, 7.4 mmol). After stirring the reaction mixture for 2 h, residual Zn was filtered off, extracted into DCM, and successively washed with H_2O and saturated $NaHCO_3$. The organic layer was dried over $MgSO_4$, filtered through Celite and concentrated. The crude residue was purified via column chromatography on silica gel eluting with EtOAc:hexanes to afford (+)-longicamphenylone.

White solid; m.p. 49-50. ^{1}H NMR (400 MHz, $CDCl_3$): δ 2.52 (dm, J = 5.4 Hz, 1H), 2.42-2.40 (m, 1H), 1.97-1.86 (m, 2H), 1.78-1.72 (m, 1H), 1.68-1.63 (m, 2H), 1.56-1.38 (m, 4H), 1.24-1.17 (m, 1H), 1.10-1.06 (m, 1H), 1.01 (s, 3H), 0.95 (s, 3H), 0.90 (s, 3H); $^{13}C\{^1H\}$ NMR (100 MHz, $CDCl_3$): δ 225.6, 60.6, 51.1, 48.6, 43.1, 42.9, 40.2, 36.7, 33.5, 29.1, 25.3, 25.2, 20.1; υ_{max} (ATR-IR): 1745 cm^{-1}; λ_{max} (CH_3CN): 290 nm; HRMS (ESI) m/z $(C_{14}H_{22}O_2)_2Na^+$: calc 467.322572, observed 467.313270.

5α-Tosyl-15-norpanasinsan-8-one[10, 11,12,13]

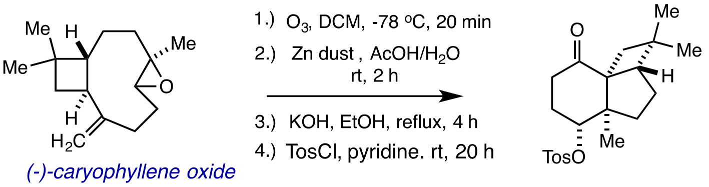

To a flame-dried three-neck round-bottom flask equipped with a stir bar was added (-)-caryophyllene oxide (5.00 g, 22.7 mmol) and DCM (120 mL). The solution was then cooled to -78 °C, purged with oxygen for 5 min, and then a stream of ozone gas was bubbled through the solution for 20 min (excess ozone was quenched by bubbling through a saturated aqueous $NaSO_3$). Subsequently, the solution was purged with oxygen for 5 min, warmed to rt under N_2, diluted with aqueous 50% AcOH (60 mL), and then treated with a Zn dust (2.23 g, 34.1 mmol). After stirring the reaction mixture for 2 h, residual Zn was filtered off, extracted into DCM, and successively washed with H_2O and saturated $NaHCO_3$. The organic layer was dried over $MgSO_4$, filtered through Celite and concentrated to afford kobusone (5.00 g, 99%) that was used without further purification.

A solution of kobusone (1.00 g, 4.5 mmol) and KOH (12.00 g, 213.9 mmol) in EtOH (120 mL) was heated to reflux for 4 h. Then, the reaction mixture was diluted with H_2O (150 mL), extracted into Et_2O, dried over MgSO4, filtered through Celite and concentrated. The residue was purified via column chromatography on silica gel eluting with EtOAc:hexanes to provide 5α-hydroxy-15-norpanasinsan-8-one (0.8 g, 80%).

To a flame-dried round-bottom equipped with a stir bar under N_2 was added 5α-hydroxy-15-norpanasinsan-8-one (0.80 g, 3.6 mmol) and pyridine (20 mL). The reaction mixture was treated with TosCl (1.16 g, 6.1 mmol), and stirred at rt for 20 h. Then, the reaction was quenched with water, diluted with EtOAc, washed successively with 1.0 M HCl, H_2O, saturated $NaHCO_3$, and brine. The organic layer was dried over $MgSO_4$, filtered through Celite, and the filtrate was concentrated. The crude residue was purified via column chromatography on silica gel eluting with EtOAc:hexanes to provide 5α-tosyl-15-norpanasinsan-8-one (0.70 g, 52%).

Viscous oil. ^{1}H NMR (400 MHz, $CDCl_3$): δ 7.83-7.79 (m, 2H), 7.36-7.33 (m, 2H), 4.72 (dd, J = 12.1, 4.4 Hz, 1H), 2.53-2.44 (m, 2H), 2.45 (s, 3H), 2.34-2.25 (m, 2H), 2.13-2.07 (m, 1H), 2.03-1.92 (m, 1H), 1.89-1.66 (m, 3H), 1.59-1.50 (m, 1H), 1.32 (d, J = 12.3 Hz, 1H), 0.96 (s, 3H), 0.84 (s, 3H), 0.83 (s, 3H); $^{13}C\{^1H\}$ NMR (100 MHz, $CDCl_3$): δ 209.8, 144.8, 134.2, 129.8, 127.6, 80.6, 58.6, 54.4, 52.6, 37.0, 35.7, 33.1, 31.1, 29.6, 27.6, 24.9, 24.4, 21.6, 12.7; υ_{max} (ATR-IR): 1699 cm^{-1}; λ_{max} (CH_3CN): 294 nm; HRMS (ESI) m/z $(C_{21}H_{28}O_4S)_2Na^+$: calc 775.330881, observed 775.330542.

3β-Chloro-5α-cholestan-7-one[3,4]

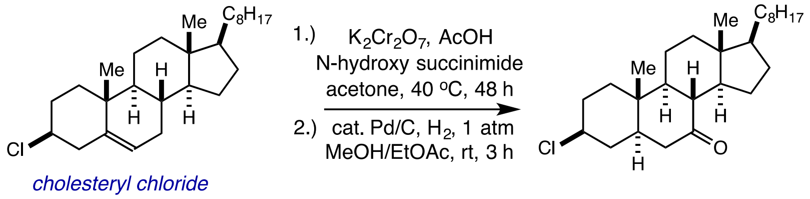

Cholesteryl chloride (5.00 g, 12.3 mmol) was dissolved in a mixture of acetone (300 mL) and acetic acid (30 mL) in a round-bottom flask equipped with a stir bar and reflux condenser under N_2. The reaction mixture was treated with *N*-hydroxysuccinimide (11.2 g, 114 mmol) and $K_2Cr_2O_7$ (14.5 g, 49.3 mmol), and then the reaction mixture was stirred at 40 °C for 48 h. The reaction mixture was cooled to rt, quenched with 10% aqueous sodium metabisulfite solution, filtered through Celite, and extracted into Et_2O. The combined organic layers were washed with saturated $NaHCO_3$ and brine, and then dried over $MgSO_4$ and concentrated. The crude residue was recrystallized in MeOH to provide 3β-chloro-cholest-5-en-7-one (3.28 g, 80%).

A balloon filled with hydrogen was placed over a round-bottom flask containing a solution of 3β-chloro-cholest-5-en-7-one (1.00 g, 2.39 mmol) and 10% Pd/C (80 mg) in MeOH (30 mL) and EtOAc (20 mL). The reaction mixture was then stirred at rt for 3 h. The catalyst was removed by filtration through Celite, and the filtrate was concentrated. The crude residue was recrystallized using MeOH to obtain 3β-chloro-5α-cholestan-7-one (1.00 g, 99%).

White solid; m.p. 128-130 °C. ^{1}H NMR (400 MHz, $CDCl_3$): δ 3.85-3.77 (m, 1H), 2.37-2.30 (m, 2H), 2.22-2.13 (m, 1H), 2.11-1.95 (m, 3H), 1.91-1.73 (m, 5H), 1.57-1.46 (m, 4H), 1.40-1.28 (m, 4H), 1.26-1.19 (m, 1H), 1.16-1.06 (m, 6H), 1.1 (s, 3H), 1.05-0.96 (m, 3H), 0.89 (d, *J* = 6.6 Hz, 3H), 0.853 (d, *J* = 6.6 Hz, 3H), 0.848 (d, *J* = 6.6 Hz, 3H), 0.64 (s, 3H); $^{13}C\{^1H\}$ NMR (100 MHz, $CDCl_3$): δ 211.3, 58.6, 55.0, 54.98, 50.0, 48.8, 48.3, 45.7, 42.5, 39.4, 39.2, 38.6, 37.7, 36.1, 35.8, 35.6, 32.7, 28.4, 28.0, 24.9, 23.7, 22.8, 22.5, 21.7, 18.8, 12.0, 11.7; υ_{max} (ATR-IR): 1699 cm^{-1}; λ_{max} (CH_3CN): 292 nm; HRMS (ESI) m/z $(C_{27}H_{45}ClO)_2Na^+$: calc 863.621009, observed 863.620294.

3,17-Diacetoxy-androstan-7-one[14,15,3,4]

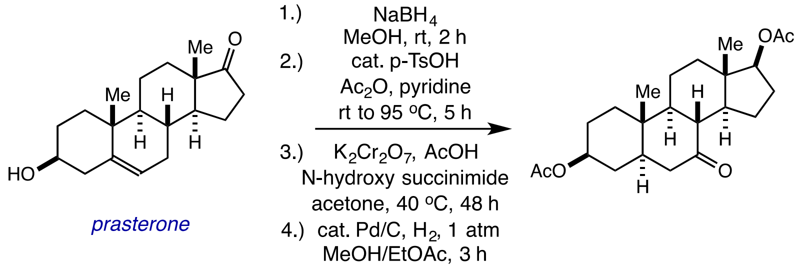

To a flame-dried round-bottom equipped with a stir bar under N_2 was added prasterone (4.00 g, 13.9 mmol) and MeOH (75 mL). The reaction mixture was treated with $NaBH_4$ (0.53 g, 13.9 mmol) in portions over 10 min, and then stirred for an additional 2 h. The

resulting white precipitate was collected by filtration and dried to provide 5-androstenediol (3.50 g, 87%).

The 5-androstenediol from the previous step (3.10 g, 10.7 mmol), p-TsOH•H_2O (60 mg, 0.30 mmol), and acetic anhydride (4.6 mL) were dissolved in pyridine (6.0 mL) under N_2. After stirring for 1 h, the reaction mixture was heated to 95 °C and stirred for an additional 3.5 h. The reaction mixture was then cooled to rt and diluted with H_2O (150 mL). The white precipitate was collected by filtration, washed with H_2O, and dried to provide androstenediol-3,17-diacetate (3.52 g, 85%).

Androstenediol-3,17-diacetate (1.93 g, 5.2 mmol) was dissolved in a mixture of acetone (200 mL) and acetic acid (20 mL) in a round-bottom flask equipped with a stir bar and reflux condenser under N_2. The reaction mixture was treated with *N*-hydroxysuccinimide (5.93 g, 52 mmol) and $K_2Cr_2O_7$ (6.06 g, 21 mmol), and then the reaction mixture was stirred at 40 °C for 48 h. The reaction mixture was cooled to rt, quenched with 10% aqueous sodium metabisulfite solution, filtered through Celite, and extracted into Et_2O. The combined organic layers were washed with saturated $NaHCO_3$ and brine, and then dried over $MgSO_4$ and concentrated. The crude residue was recrystallized in MeOH to provide 3β,17β-diacetoxyandrost-5-ene-7-one (1.64 g, 82%).

A balloon filled with hydrogen was placed over a round-bottom flask containing a solution of 3β,17β-diacetoxyandrost-5-ene-7-one (1.00 g, 2.57 mmol) and 10% Pd/C (140 mg) in MeOH (40 mL) and EtOAc (10 mL). The reaction mixture was then stirred at rt for 3 h. The catalyst was removed by filtration through Celite, and the filtrate was concentrated. The crude residue was recrystallized using MeOH to afford 3,17-diacetoxy-androstan-7-one (0.70 g, 70%).

White solid; m.p. 189-191 ºC. ^{1}H NMR (400 MHz, $CDCl_3$): δ 4.69-4.58 (m, 2H), 2.38-2.28 (m, 2H), 2.26-2.13 (m, 2H), 2.06-1.96 (m, 1H), 2.01 (s, 3H), 2.00 (s, 3H), 1.90-1.85 (m, 1H), 1.80-1.40 (m, 10H), 1.26-1.17 (m, 1H), 1.14-1.01 (m, 3H), 1.08 (s, 3H), 0.76 (s, 3H); $^{13}C\{^1H\}$ NMR (100 MHz, $CDCl_3$): δ 210.5, 170.9, 170.3, 81.9, 72.5, 54.8, 49.5, 46.0, 45.5, 43.7, 42.5, 35.8, 35.72, 35.70, 33.7, 27.4, 27.0, 24.5, 21.24, 21.20, 21.0, 12.0, 11.6; υ_{max} (ATR-IR): 1724, 1706 cm^{-1}; λ_{max} (CH_3CN): 292 nm; HRMS (ESI) m/z $C_{23}H_{34}O_5Na^+$: calc 413.229845, observed 413.230264.

3β,20α-Diacetoxy-5α-pregnan-7-one[14,15,3,4]

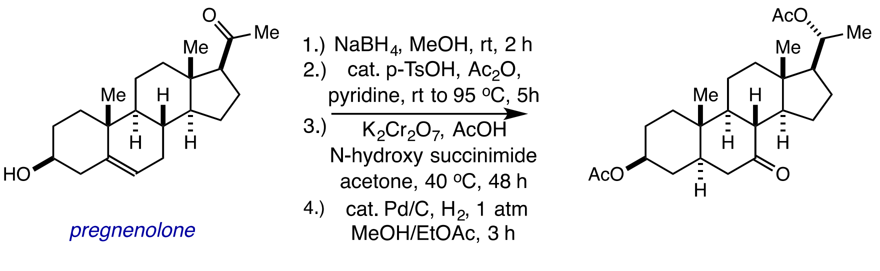

To a flame-dried round-bottom flask equipped with a stir bar under N_2 was added pregnenolone (4.00 g, 12.6 mmol) and MeOH (80 mL). The reaction mixture was treated with $NaBH_4$ (0.96 g, 25.3 mmol) in portions over 10 min, and then stirred for an additional 2 h. The resulting white precipitate was collected by filtration and dried to provide pregn-5-ene-3β,20α-diol (3.00 g, 75%).

The pregn-5-ene-3β,20α-diol from the previous step (2.50 g, 7.9 mmol), p-TsOH•H_2O (48 mg, 0.24 mmol), and acetic anhydride (4 mL) were dissolved in pyridine (5 mL) under N_2. After stirring for 1 h, the reaction mixture was heated to 95 °C and stirred for an additional 4 h. The reaction mixture was then cooled to rt and diluted with H_2O (130 mL). The white precipitate was collected by filtration, washed with H_2O, and dried to provide pregn-5-en-3β,20α-diyl diacetate (2.40 g, 76%).

Pregn-5-en-3β,20α-diyl diacetate (2.40 g, 5.0 mmol) was dissolved in a mixture of acetone (300 mL) and acetic acid (30 mL) in a round-bottom flask equipped with a stir bar and reflux condenser under N_2. The reaction mixture was treated with *N*-hydroxysuccinimide (6.90 g, 60 mmol) and $K_2Cr_2O_7$ (7.06 g, 24 mmol), and then the reaction mixture was stirred at 40 °C for 48 h. The reaction mixture was cooled to rt, quenched with 10% aqueous sodium metabisulfite solution, filtered through Celite, and extracted into Et_2O. The combined organic layers were washed with saturated aqueous $NaHCO_3$ and brine, and then dried over $MgSO_4$ and concentrated. The crude residue was recrystallized in MeOH to provide 3β,20α-diacetoxypregn-5-en-7-one (1.56 g, 75%).

A balloon filled with hydrogen was placed over a round-bottom flask containing a solution of 3β,20α-diacetoxypregn-5-en-7-one (0.80 g, 1.9 mmol) and 10% Pd/C (140 mg) in MeOH (40 mL) and EtOAc (10 mL). The reaction mixture was then stirred at rt for 3 h. The catalyst was removed by filtration through Celite, and the filtrate was concentrated. The crude residue was purified via column chromatography eluting with EtOAc:hexanes to provide 3β,20α-diacetoxy-5α-pregnan-7-one (0.60 g, 76%).
White solid; m.p. 149-150 ºC. ^1H NMR (400 MHz, $CDCl_3$): δ 4.87-4.79 (m, 1H), 4.71-4.63 (m, 1H), 2.37-2.23 (m, 3H), 2.07-2.04 (m, 1H), 2.01 (s, 3H), 2.00 (s, 3H), 1.93-1.86 (m, 1H), 1.82-1.75 (m, 3H), 1.68-1.42 (m, 8H), 1.25-1.00 (m, 5H), 1.14 (d, J = 6.1 Hz, 3H), 1.08 (s, 3H), 0.61 (s, 3H); $^{13}C\{^1H\}$ NMR (100 MHz, $CDCl_3$): δ 211.1, 170.4, 170.3, 72.7, 72.6, 54.9, 53.9, 49.7, 48.3, 46.3, 45.7, 42.3, 38.1, 35.9, 35.8, 33.8, 27.1, 25.6, 24.9, 21.7, 21.5, 21.3, 19.9, 12.5, 11.7; υ_{max} (ATR-IR): 1729, 1708 cm^{-1}; λ_{max} (CH_3CN): 292 nm; HRMS (ESI) m/z $C_{25}H_{38}O_5Na^+$: calc 441.261145, observed 441.261728.

<u>17β-Acetoxy-5α-androstan-6-one</u>[13,14,15,8,9,4]

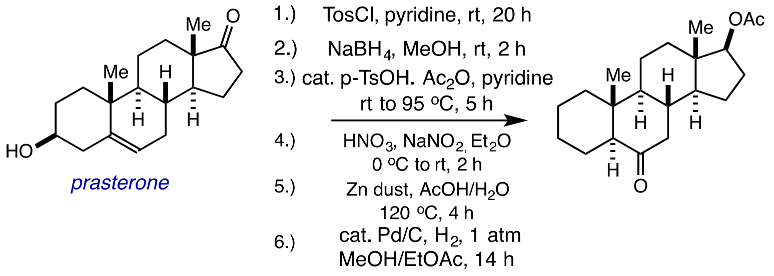

To a flame-dried round-bottom equipped with a stir bar under N_2 was added prasterone (5.00 g, 17.3 mmol) and pyridine (70 mL). The reaction mixture was treated with TosCl (5.00 g, 26.2 mmol), and stirred at rt for 20 h. The reaction was then quenched with H_2O, diluted with EtOAc, washed successively with 1.0 M HCl, H_2O, saturated $NaHCO_3$, and brine. The organic layer was dried over $MgSO_4$, filtered through Celite, and the filtrate

was concentrated. The crude residue was purified via column chromatography eluting with EtOAc:hexanes to provide 3β-tosyloxyandrost-5-ene-17-one (5.60 g, 74%).

The 3β-tosyloxyandrost-5-ene-17-one (4.50 g, 10.0 mmol) was dissolved in MeOH (65 mL), and reaction mixture was treated with $NaBH_4$ (0.76 g, 20.0 mmol) in portions over 10 min, and then stirred for an additional 2 h. The resulting white precipitate was collected by filtration and dried to provide 3β-tosyloxyandrost-5-ene-17β-ol (4.30 g, 75%).

The 3β-tosyloxyandrost-5-ene-17β-ol from the previous step (4.30 g, 9.0 mmol), p-TsOH•H_2O (60 mg, 0.30 mmol), and acetic anhydride (30 mL) were dissolved in pyridine (30 mL) under N_2. After stirring for 1 h, the reaction mixture was heated to 95 °C and stirred for an additional 4 h. The reaction mixture was then cooled to rt and diluted with H_2O (150 mL). The white precipitate was collected by filtration, washed with H_2O, and dried over $MgSO_4$ to provide 17β-acetoxy-3β-p-tolylsulphonyloxyandrost-5-ene (4.25 g, 87%).

The 17β-acetoxy-3β-p-tolylsulphonyloxyandrost-5-ene (4.25 g, 8.7 mmol) was dissolved in Et_2O (85 mL) using 500 mL three-neck round-bottom flask equipped with a stir bar and condenser. After cooling the solution to 0 ºC, aqueous 60-70% HNO_3 (64 mL) was added dropwise over 20 min, and the reaction mixture was stirred for additional 5 min. At this point, $NaNO_2$ (0.90 g, 13.1 mmol) was added, and the reaction mixture was slowly warmed to rt over 2 h. The reaction mixture was then transferred to a separatory funnel containing 60 mL of cold water. The aqueous layer was removed without agitation, and the Et_2O layer was washed with cold H_2O, 1.0 M NaOH, and then H_2O. The combined organic layers were dried over Na_2SO_4, filtered through Celite, and concentrated. The crude residue (3.65 g) was dissolved in AcOH (70 mL) and diluted with H_2O (7 mL) in a round-bottom flask equipped with a stir bar and a condenser. The resulting reaction mixture was treated with Zn dust in portions over 30 min at rt, and was then heated to reflux for 4 h. Upon cooling to rt, the mixture was diluted with EtOAc, filtered through Celite (to remove residual Zn and related byproducts). The organic layer was washed with H_2O, dried over $MgSO_4$, filtered through Celite, and concentrated. The crude residue was purified via column chromatography eluting with EtOAc:hexanes to provide 17β-acetoxy-5α-androst-2-en-6-one (0.90 g, 39%).

A balloon filled with hydrogen was placed over a round-bottom flask containing a solution of 17β-acetoxy-5α-androst-2-en-6-one (0.65 g, 2.0 mmol) and 10% Pd/C (100 mg) in MeOH (32 mL) and EtOAc (8 mL). The reaction mixture was then stirred at rt for 14 h. The catalyst was removed by filtration through Celite, and the filtrate was concentrated. The crude residue was purified via column chromatography eluting with EtOAc:hexanes to provide 17β-acetoxy-5α-androstan-6-one (0.40 g, 60%).

White solid; m.p. 124-126 ºC. ^{1}H NMR (400 MHz, $CDCl_3$): δ 4.63-4.58 (m, 1H), 2.27 (dd, J = 13.0, 4.5 Hz, 1H), 2.20-2.10 (m, 2H), 2.02 (s, 3H), 1.96 (td, J = 12.7, 1.2 Hz, 1H), 1.86-1.65 (m, 5H), 1.60-1.33 (m, 6H), 1.31-1.19 (m, 5H), 1.17-1.07 (m, 2H), 0.76 (s, 3H), 0.71 (s, 3H); $^{13}C\{^1H\}$ NMR (100 MHz, $CDCl_3$): δ 212.0, 171.0, 82.3, 58.8, 54.2, 51.2, 46.2, 43.0, 41.7, 38.1, 37.6, 36.5, 27.3, 25.1, 23.2, 21.3, 21.1, 20.6, 20.4, 13.0, 12.0; υ_{max} (ATR-IR): 1734, 1710 cm^{-1}; λ_{max} (CH_3CN): 291 nm; HRMS (ESI) m/z $C_{21}H_{32}O_3Na^+$: calc 355.224366, observed 355.224678.

3β-Acetoxy-D-homo-5α-androstan-17a-one[16,17]

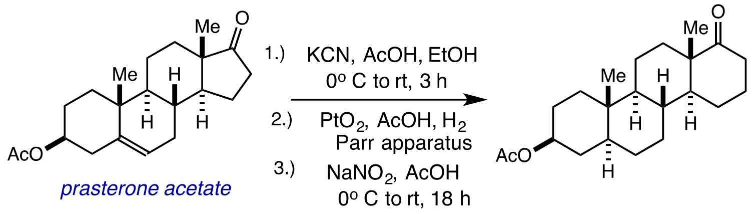

Prasterone acetate (5.00 g, 15.1 mmol) was dissolved in EtOH (150 mL) and treated with KCN (31.5 g, 484 mmol) while stirring. The reaction mixture was cooled to 0 °C and AcOH (35 mL) was added dropwise; the reaction mixture was stirred for 1 h. The reaction mixture was stirred at rt for an additional 2 h and then quenched with H_2O. The white precipitate was collected by filtration, washed with H_2O, washed with 2% aqueous AcOH, and then dried over $MgSO_4$. The crude residue (4.80 g, 12.4 mmol), PtO_2 (1.00 g), and AcOH (150 mL) were shaken under H_2 at 40 psi in a Parr apparatus for 48 h. The solution was filtered through Celite, concentrated, and diluted with water (80 mL). Neutral impurities were removed by extracting into Et_2O. The aqueous layer was then transferred to a round-bottom flask, along with AcOH (10 mL), and cooled to 0 °C. Then, $NaNO_2$ (2.40 g, 34.8 mmol) dissolved in water (8 mL) was added to the reaction mixture, which was then stirred for 2 h at 0 °C. The reaction mixture was warmed to rt and stirred for additional 16 h. The precipitated white solid was collected via filtration, washed with H_2O, and dried. The crude residue was purified via column chromatography eluting with EtOAc:hexanes to provide 3β-acetoxy-D-homo-5α-androstan-17a-one (2.40 g, 56%).

White solid; m.p. 118-120 °C. ^{1}H NMR (400 MHz, $CDCl_3$): δ 4.70-4.61 (m, 1H), 2.59 (td, J = 14.0, 6.7 Hz, 1H), 2.17 (dm, J = 14.1 Hz, 1H), 2.06-1.95 (m, 1H), 1.99 (s, 3H), 1.89-1.69 (m, 5H), 1.65-1.50 (m, 3H), 1.48-1.27 (m, 6H), 1.25-1.09 (m, 4H), 1.06 (s, 3H), 1.02-0.94 (m, 1H), 0.87-0.74 (m, 1H), 0.79 (s, 3H), 0.68-0.61 (m, 1H); $^{13}C\{^1H\}$ NMR (100 MHz, $CDCl_3$): δ 216.4, 170.6, 73.5, 53.2, 51.4, 48.3, 43.9, 37.1, 36.4, 35.6, 35.1, 33.8, 32.4, 31.2, 28.4, 27.3, 25.9, 22.9, 21.4, 19.9, 16.9, 12.1; υ_{max} (ATR-IR): 1732, 1701 cm^{-1}; λ_{max} (CH_3CN): 293 nm; HRMS (ESI) m/z $C_{22}H_{34}O_3Na^+$: calc 369.240016, observed 369.240001.

3β-Tosyloxy-5α-androstan-17-one[13,4]

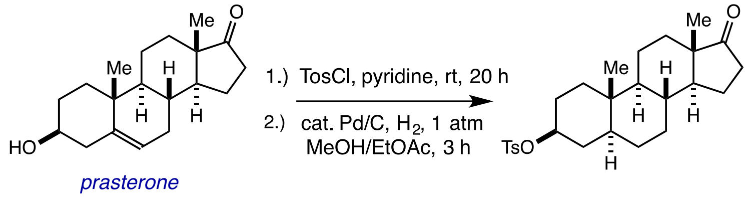

To a flame-dried round-bottom equipped with a stir bar under N_2 was added prasterone (5.00 g, 17.3 mmol) and pyridine (70 mL). The reaction mixture was treated with tosyl chloride (5.00 g, 26.2 mmol), and stirred at rt for 20 h. The reaction was then quenched

with H_2O, diluted with EtOAc, washed successively with 1.0 M HCl, H_2O, saturated $NaHCO_3$, and brine. The organic layer was dried over $MgSO_4$, filtered through Celite, and the filtrate was concentrated. The crude residue was purified via column chromatography on silica gel eluting with EtOAc:hexanes to provide 3β-tosyloxyandrost-5-ene-17-one (5.60 g, 74%).

A balloon filled with hydrogen was placed over a round-bottom flask containing a solution of 3β-tosyloxyandrost-5-ene-17-one (0.65 g, 1.5 mmol) and 10% Pd/C (80 mg) in MeOH (24 mL) and EtOAc (6 mL). The reaction mixture was then stirred at rt for 3 h. The catalyst was removed by filtration through Celite, and the filtrate was concentrated. The crude residue was purified via column chromatography eluting with EtOAc:hexanes to provide 3β-tosyloxy-5α-androstan-17-one (0.60 g, 90%).

White solid; m.p. 157-158 ºC. ^{1}H NMR (400 MHz, $CDCl_3$): δ 7.78 (d, J = 8.3 Hz, 2H), 7.32 (d, J = 8.0 Hz, 2H), 4.44-4.36 (m, 1H), 2.45-2.38 (m, 1H), 2.43 (s, 3H), 2.09-2.00 (m, 1H), 1.93-1.87 (m, 1H), 1.79-1.71 (m, 3H), 1.68-1.44 (m, 7H), 1.33-1.17 (m, 5H), 1.13-1.05 (m, 1H), 0.98-0.87 (m, 2H), 0.83 (s, 3H), 0.80 (s, 3H), 0.67-0.60 (m, 1H); $^{13}C\{^1H\}$ NMR (100 MHz, $CDCl_3$): δ 221, 144.3, 134.7, 129.7, 127.6, 82.1, 54.1, 51.3, 47.7, 44.8, 36.7, 35.8, 35.3, 34.9, 34.8, 31.4, 30.7, 28.3, 28.1, 21.7, 21.6, 20.4, 13.8, 12.1; υ_{max} (ATR-IR): 1734 cm^{-1}; λ_{max} (CH_3CN): 298 nm; HRMS (ESI) m/z $C_{26}H_{36}O_4SNa^+$: calc 467.222651, observed 467.222407.

Methyl 5,17-dioxo-A-nor-3,4-seco-androstan-3-oate[18,19,20,21]

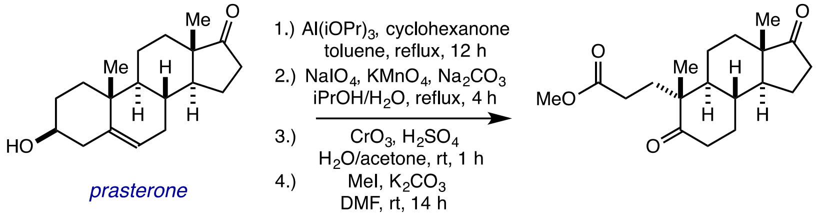

To an oven-dried round-bottom flask equipped with a stir bar under N_2 was added prasterone (8.50 g, 29.5 mmol), aluminum isopropoxide (17.20 g, 84.0 mmol), cyclohexanone (14 mL) and dry toluene (45 mL). The mixture was refluxed for 12 h and then cooled to rt, quenched with H_2O, diluted with EtOAc and filtered through Celite. The organic layer was separated, washed with brine, dried over Na_2SO_4 and concentrated to provide testosterone. The crude residue was purified via column chromatography on silica gel eluting with EtOAc:hexanes (4.67 g, 55%).

To a round-bottom flask containing testosterone (4.70 g, 16.3 mmol) was added isopropanol (65 mL) followed by Na_2CO_3 (2.00 g, 18.8 mmol). A preheated solution of $NaIO_4$ (19.2 g, 89.7 mmol) and $KMnO_4$ (0.13 g, 0.82 mmol) in H_2O (54 mL) was added dropwise over 30 min using an addition funnel. The mixture was refluxed for 4 h, and was then cooled to rt. The reaction mixture was concentrated and then was acidified with 1.0 M HCl, extracted into DCM, washed with brine, dried over Na_2SO_4, filtered through Celite, and concentrated to provide 5-oxo-A-nor-3,5-seco-17β-hydroxy-androstan-3-oic acid that was used without a purification in the next step.

The 5-oxo-A-nor-3,5-seco-17β-hydroxy-androstan-3-oic from the previous step (884 mg, 2.9 mmol) was dissolved in acetone (29 mL) and the mixture was cooled to 0 °C.

Jones reagent (4.4 mmol) was then added dropwise, the mixture was warmed to rt, and stirred for 1 h. The reaction mixture was quenched with H_2O, extracted into DCM, washed with brine, dried over Na_2SO_4, filtered through Celite, and concentrated to provide A-nor-3,5-seco-5,17-diketo-androstan-3-oic acid, which was used without purification in the next step.

To an oven-dried round-bottom flask was added the crude product from the previous step (0.21 g, 0.69 mmol), K_2CO_3 (0.19 g, 1.4 mmol), and dry DMF (2.3 mL). The reaction mixture was stirred for 30 min at rt. Iodomethane (0.05 mL, 0.82 mmol) was then added, and the reaction mixture was stirred for 14 h. At this point, the reaction mixture was quenched with H_2O, extracted into Et_2O, washed with brine, dried over Na_2SO_4, filtered through Celite, and concentrated. The residue was purified via column chromatography on silica gel eluting with EtOAc:hexanes to provide methyl 5,17-dioxo-A-nor-3,4-seco-androstan-3-oate (73 mg, 33%).

Colorless oil. ^{1}H NMR (400 MHz, $CDCl_3$): δ 3.60 (s, 3H), 2.58-2.40 (m, 2H), 2.30-2.22 (m, 2H), 2.16-1.93 (m, 5H), 1.61-1.49 (m, 4H), 1.32-1.20 (m, 4H), 1.09 (s, 3H), 0.88 (s, 3H); $^{13}C\{^1H\}$ NMR (100 MHz, $CDCl_3$): δ 219.7, 213.6, 174.0, 51.4, 50.6, 50.3, 47.7, 47.4, 37.6, 35.5, 34.3, 30.9, 29.8, 29.4, 29.0, 21.7, 20.6, 20.3, 13.6; υ_{max} (ATR-IR): 1740, 1736, 1702 cm^{-1}; λ_{max} (CH_3CN): 293 nm; HRMS (ESI) m/z $C_{19}H_{28}O_4Na^+$: calc 343.187980, observed 343.187631.

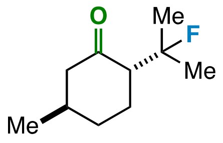

Table 2. Compound 3. The reaction was run according to the general procedure, and the product yield was determined by ^{19}F NMR analysis. Spectral data match the literature for this compound.[22]

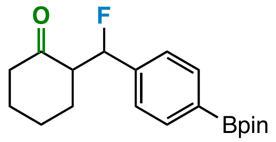

Table 2. Compound 4. The reaction was run according to the general procedure, and the minor diastereomer was isolated.

White solid; m.p. 94-95 °C. ^{1}H NMR (400 MHz, $CDCl_3$): 7.81 (2H, d, *J* = 7.6 Hz), 7.31 (2H, d, *J* = 8.2 Hz), 6.11 (1H, dd, *J* = 46.6, 3.9 Hz), 2.72-2.60 (1H, m), 2.50-2.44 (1H, m), 2.33-2.24 (1H, m), 2.09-2.00 (2H, m), 1.95-1.88 (1H, m), 1.78-1.66 (2H, m), 1.61-1.49 (1H, m), 1.34 (12H, s); ^{13}C{^{1}H} NMR (100 MHz, $CDCl_3$): 209.1 (d, *J* = 2.2 Hz), 142.3, 142.1, 134.75, 134.74, 124.7, 124.6, 90.5 (d, *J* = 175.1 Hz), 83.8, 56.2 (d, *J* = 22.9 Hz), 42.2, 27.0, 26.24, 26.18, 24.85, 24.84, 24.5; ^{19}F NMR (282 MHz, $CDCl_3$): -193.7 (1F, dd, *J* = 46.5, 24.1 Hz); υ_{max} (ATR-IR): 1710 cm^{-1}; HRMS (ESI) m/z $C_{19}H_{26}BFO_3Na^+$: calc 355.185124, observed 355.184717.

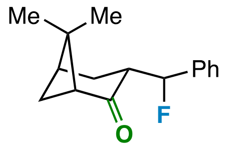

Table 2. Compound 5. The reaction was run according to the general procedure, and the product was isolated as a mixture of diastereomers.

Colorless oil. ^{1}H NMR (400 MHz, $CDCl_3$): 7.41-7.27 (5H, m), 6.19 (1H, dd, *J* = 44.4, 5.5 Hz), 3.45-3.32 (1H, m), 2.51-2.47 (1H, m), 2.23-1.97 (4H, m), 1.28 (3H, s), 0.95 (3H, s), 0.40 (1H, d, *J* = 10.7 Hz); ^{13}C{^{1}H} NMR (100 MHz, $CDCl_3$): 210.8 (d, *J* = 10.0 Hz), 137.7, 137.5, 128.4, 128.3, 126.2, 126.1, 94.2 (d, *J* = 175.8 Hz), 58.3 (d, *J* = 2.9 Hz), 49.5 (d, *J* = 23.6 Hz), 40.6, 39.9, 25.8, 23.8, 22.7 (d, *J* = 2.2 Hz), 22.0; ^{19}F NMR (282 MHz, $CDCl_3$): -185.0 (1F, dd, *J* = 44.2, 14.9 Hz), -192.4 (dd, *J* = 47.6, 41.3 Hz); υ_{max} (ATR-IR): 1706 cm^{-1}; HRMS (ESI) m/z $C_{16}H_{19}FONa^+$: calc 269.131215, observed 269.130823.

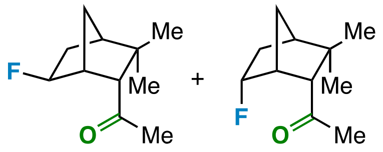

Table 2. Compound 6a and 6b. The reaction was run according to the general procedure, and product was isolated as a mixture of diastereomers.

Colorless oil. ^{1}H NMR (400 MHz, $CDCl_3$): 5.67-4.81 (1H, m), 2.66-2.16 (3H, m), 2.13-1.89 (4H, m), 1.68-1.31 (3H, m), 1.26-1.24 (3H, m), 0.95-0.90 (3H, m); ^{13}C{^{1}H} NMR (100 MHz, $CDCl_3$): 209.8, 209.67, 209.66, 93.9, 92.2, 92.1, 90.5, 71.72, 61.70, 60.9, 60.8, 55.1, 54.9, 48.89, 48.88, 47.8, 47.6, 35.0, 33.81, 33.80, 33.5, 32.5, 32.3, 32.07, 32.06, 32.0, 22.7, 22.2; ^{19}F NMR (282 MHz, $CDCl_3$): -167.6 (1F, dddd, J = 55.6, 41.3, 22.9, 8.0 Hz), -168.33 (1F, ddddd, J = 55.6, 41.3, 15.5, 10.3, 5.2 Hz); υ_{max} (ATR-IR): 1703 cm^{-1}.

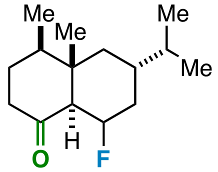

Table 2. Compound 7. The reaction was run according to the general procedure, and the major diastereomer was isolated. Regiochemical assignment was made on the basis of 1) chemical shift in the ^{19}F NMR spectrum that indicates a secondary fluoride on a cyclohexane ring and 2) identification of $^{2}J_{CF}$- and $^{3}J_{CF}$-coupling to distinguishable peaks in the ^{13}C NMR spectrum, i.e. C4a, C6, C7, and C8a *vide infra*. Stereochemical assignment was made on the basis of 1) chemical shift and splitting in the ^{19}F NMR spectrum that indicates F_{eq} on a cyclohexane ring and 2) identification of the two distinct *trans* diaxial $^{3}J_{HH}$-coupling constants (11.1 and 10.3 Hz) in the ^{1}H NMR spectrum.

Viscous oil. ^{1}H NMR (400 MHz, $CDCl_3$): 4.96 (1H, dddd, J = 48.1, 11.1, 10.3, 5.3 Hz), 2.45 (1H, td, J = 13.2, 7.3 Hz), 2.37-2.30 (2H, m), 2.21-2.14 (1H, m), 1.95-1.89 (1H, m), 1.87-1.78 (1H, m), 1.71-1.60 (2H, m), 1.55-1.43 (1H, m), 1.40-1.30 (1H, m), 1.13 (1H, m), 0.97-0.92 (1H, m), 0.90-0.86 (9H, m), 0.63 (3H, s); ^{13}C{^{1}H} NMR (100 MHz, $CDCl_3$): 209.4, 87.5 (d, J = 170.3 Hz, C8), 62.5 (d, J = 15.8 Hz, C8a), 44.0 (d, J = 7.4 Hz, C4a), 42.8 (d, J = 1.1 Hz), 41.8, 41.3 (d, J = 1.8 Hz), 36.5 (d, J = 10.3 Hz, C6), 34.8 (d, J = 18.1 Hz, C7), 32.4 (d, J = 1.1 Hz), 32.0, 19.9, 19.3, 14.4, 13.1; ^{19}F NMR (282 MHz, $CDCl_3$): -177.0 (1F, dm, J = 48.2 Hz); υ_{max} (ATR-IR): 1720 cm^{-1}; HRMS (ESI) m/z $C_{15}H_{25}FONa^{+}$: calc 263.178165, observed 263.177975.

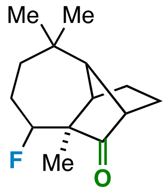

Table 2. Compound 8. The reaction was run according to the general procedure, and the major diastereomer was isolated.

Regiochemical assignment was made on the basis of 1) chemical shift and $^{2}J_{HF}$-coupling in the ^{19}F NMR spectrum that indicates a secondary fluoride and 2) identification of $^{2}J_{CF}$- and $^{3}J_{CF}$-coupling to distinguishable peaks in the ^{13}C NMR spectrum, i.e. C4, C6, and C9 (carbonyl carbon) *vide infra*. Stereochemistry was not assigned.

White solid; m.p. 64-67 °C. ^{1}H NMR (400 MHz, $CDCl_3$): 4.53 (1H, dm, *J* = 46.3 Hz), 2.63 (1H, dm, *J* = 5.3 Hz), 2.29 (1H, br s), 2.00-1.85 (3H, m), 1.83-1.77 (1H, ddd, *J* = 12.4, 9.1, 3.2 Hz), 1.70 (1H, br s), 1.68-1.60 (1H, m), 1.48-1.42 (1H, m), 1.25 (3H, s), 1.20-1.07 (2H, m), 1.04 (3H, s), 0.97 (3H, s); ^{13}C{^{1}H} NMR (100 MHz, $CDCl_3$): 219.0 (d, *J* = 2.6 Hz), 99.8 (d, *J* = 180.6 Hz), 60.2, 51.9, 51.2 (d, *J* = 15.8 Hz), 42.6 (d, *J* = 5.9 Hz), 33.3, 31.9, 30.6, 28.6, 27.7 (d, *J* = 23.6 Hz), 26.0, 24.7 (d, *J* = 1.8 Hz), 22.4 (d, *J* = 1.8 Hz); ^{19}F NMR (282 MHz, $CDCl_3$): -181.9 (1F, dm, *J* = 47.0 Hz);
υ_{max} (ATR-IR): 1741 cm^{-1}; HRMS (ESI) m/z $C_{14}H_{21}FONa^{+}$: calc 247.146865, observed 247.146615.

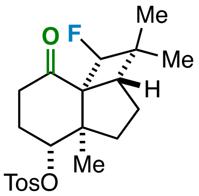

Table 2. Compound 9. The reaction was run according to the general procedure, and the major diastereomer was isolated.

Regiochemical assignment was made on the basis of 1) chemical shift and $^{2}J_{HF}$-coupling in the ^{19}F NMR spectrum as well as the large, diagnostic $^{1}J_{CF}$-coupling in the ^{13}C NMR spectrum that indicate a secondary fluoride on a cyclobutane ring and 2) identification of $^{2}J_{CF}$- and $^{3}J_{CF}$-coupling to distinguishable peaks in the ^{13}C NMR spectrum *vide infra*. Stereochemistry was not assigned.

Viscous oil. ^{1}H NMR (400 MHz, $CDCl_3$): 7.82-7.78 (2H, m), 7.37-7.33 (2H, m), 4.51 (1H, dd, *J* = 53.0, 0.9 Hz), 4.39 (1H, dd, *J* = 10.9, 6.3 Hz), 2.48-2.45 (1H, m), 2.46 (3H, s), 2.44-2.35 (2H, m), 2.13-2.01 (2H, m), 1.92-1.87 (1H, m), 1.64-1.47 (2H, m), 1.36-1.30 (1H, m), 1.23-1.22 (6H, m), 0.95 (3H, d, *J* = 0.8 Hz); ^{13}C{^{1}H} NMR (100 MHz, $CDCl_3$): 206.8 (d, *J* = 1.1 Hz), 145.1, 134.0, 129.9, 127.7, 93.3 (d, *J* = 233.7 Hz), 79.3 (d, *J* = 1.8 Hz), 65.2 (d, *J* = 20.6 Hz), 50.2 (d, *J* = 1.5 Hz), 49.1 (d, *J* = 10.0 Hz), 39.5 (d, *J* =

22.1 Hz), 37.7, 37.3, 25.8, 23.1, 22.4, 21.7, 21.6 (d, J = 9.2 Hz), 13.9; ^{19}F NMR (282 MHz, $CDCl_3$): -192.7 (1F, dd, J = 53.4, 7.5 Hz); υ_{max} (ATR-IR): 1706 cm^{-1}; HRMS (ESI) m/z $C_{21}H_{27}FO_4SNa^+$: calc 417.150629, observed 417.149999.

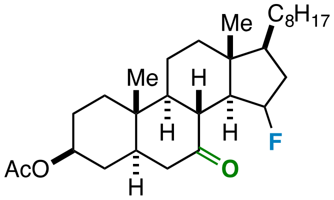

Table 3. Compound 2. The reaction was run according to the general procedure, and the major diastereomer was isolated. Regiochemical assignment was made on the basis of 1) chemical shift in the ^{19}F NMR spectrum that indicates a secondary fluoride, 2) $^2J_{HF}$-coupling in the ^{1}H and ^{19}F NMR spectra that indicates cyclopentane ring fluorination (54.9 Hz), and 3) identification of $^2J_{CF}$- and $^3J_{CF}$-coupling to distinguishable peaks in the ^{13}C NMR spectrum, i.e. C13, C14, and C16 *vide infra*. Stereochemical assignment was made on the basis of 1) a larger $^3J_{HH}$-coupling constant assigned to the triplet (8.5 Hz) than the doublet (2.5 Hz) in the ^{1}H NMR spectrum, indicative of two protons *trans* to the C15 proton and 2) analogy to X-ray crystal structure of compound **12**.

White solid; m.p. 119-120 °C. ^{1}H NMR (400 MHz, $CDCl_3$): 4.78 (1H, dtd, J = 54.9, 8.5, 2.5 Hz), 4.71-4.63 (1H, m), 2.55 (1H, t, J = 11.3 Hz), 2.35 (1H, t, J = 13.1 Hz), 2.18-2.04 (2H, m), 2.02 (3H, s), 1.98-1.75 (5H, m), 1.71-1.60 (2H, m), 1.58-1.42 (6H, m), 1.39-1.29 (3H, m), 1.27-1.12 (5H, m), 1.11-1.09 (4H, m), 1.07-0.99 (1H, m), 0.91 (3H, d, J = 6.6 Hz), 0.86 (3H, d, J = 6.6 Hz), 0.85 (3H, d, J = 6.7 Hz), 0.68 (3H, s); ^{13}C{^{1}H} NMR (100 MHz, $CDCl_3$): 209.3, 170.4, 95.2 (d, J = 179.9 Hz, C15), 72.6, 54.8, 54.4 (d, J = 17.3 Hz, C14), 52.6, 48.7, 46.1, 45.8, 44.0 (d, J = 5.9 Hz, C13), 39.4, 38.6, 37.7, 37.4, 36.0, 35.9 (d, J = 19.2 Hz, C16), 34.9, 33.7, 28.0, 27.1, 23.7, 22.8, 22.5, 21.5, 21.3, 18.5, 13.2, 11.7; ^{19}F NMR (282 MHz, $CDCl_3$): -166.8 (1F, dm, J = 55.6 Hz); υ_{max} (ATR-IR): 1736, 1716 cm^{-1}; HRMS (ESI) m/z $C_{29}H_{47}FO_3Na^+$: calc 485.340145, observed 485.340635.

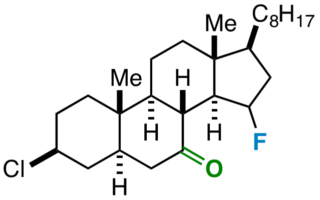

Table 3. Compound 10. The reaction was run according to the general procedure, and the major diastereomer was isolated. Regiochemical assignment was made on the basis of 1) chemical shift in the ^{19}F NMR spectrum that indicates a secondary fluoride, 2) $^2J_{HF}$-coupling in the ^{1}H and ^{19}F NMR spectra that indicates cyclopentane ring fluorination (>50 Hz), and 3) identification of $^2J_{CF}$- and $^3J_{CF}$-coupling to distinguishable peaks in the

^{13}C NMR spectrum, i.e. C13, C14, and C17 *vide infra*. Stereochemical assignment was made on the basis of 1) a larger $^3J_{HH}$-coupling constant assigned to the triplet (8.7 Hz) than the doublet (2.2 Hz) in the ^1H NMR spectrum, indicative of two protons *trans* to the C15 proton and 2) analogy to X-ray crystal structure of compound **12**.

White solid; m.p. 107-109 °C. ^1H NMR (400 MHz, $CDCl_3$): 4.75 (1H, dtd, J = 55.1, 8.7, 2.2 Hz), 3.86-3.78 (1H, m), 2.56 (1H, t, J = 11.2 Hz), 2.37 (1H, t, J = 13.3 Hz), 2.17-2.03 (3H, m), 1.98-1.68 (7H, m), 1.63-1.42 (5H, m), 1.38-1.12 (8H, m), 1.11 (3H, s), 1.08-1.01 (2H, m), 0.91 (3H, d, J = 6.6 Hz), 0.86 (3H, d, J = 6.7 Hz), 0.85 (3H, d, J = 6.6 Hz), 0.68 (3H, s); $^{13}C\{^1H\}$ NMR (100 MHz, $CDCl_3$): 209.1, 95.2 (d, J = 180.2 Hz, C15), 58.4, 54.9, 54.3 (d, J = 16.6 Hz, C14), 52.6, 48.8, 47.9, 45.7, 44.0 (d, J = 5.9 Hz, C13), 39.4, 39.1, 38.6, 37.7, 37.5 (d, J = 24.7 Hz, C16), 36.0, 35.9, 34.9, 32.6, 28.0, 23.7, 22.8, 22.5, 21.4, 18.5, 13.2, 11.7; ^{19}F NMR (282 MHz, $CDCl_3$): -168.7 (1F, dm, J = 55.1 Hz); υ_{max} (ATR-IR): 1717 cm^{-1}; HRMS (ESI) m/z $C_{27}H_{44}ClFONa^+$: calc 461.295670, observed 461.295693.

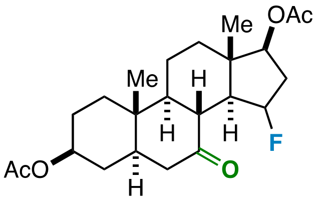

Table 3. Compound 11. The reaction was run according to the general procedure, and the major diastereomer was isolated. Regiochemical assignment was made on the basis of 1) chemical shift in the ^{19}F NMR spectrum that indicates a secondary fluoride, 2) $^2J_{HF}$-coupling in the ^1H and ^{19}F NMR spectra that indicates cyclopentane ring fluorination (>50 Hz), and 3) identification of $^2J_{CF}$- and $^3J_{CF}$-coupling to distinguishable peaks in the ^{13}C NMR spectrum, i.e. C13, C14, and C17 *vide infra*. Stereochemical assignment was made on the basis of 1) a larger $^3J_{HH}$-coupling constant assigned to the triplet (8.9 Hz) than the doublet (3.0 Hz) in the ^1H NMR spectrum, indicative of two protons *trans* to the C15 proton and 2) analogy to X-ray crystal structure of compound **12**.

White solid; m.p. 159-160.5 °C. ^1H NMR (400 MHz, $CDCl_3$): 4.87 (1H, t, J = 8.9 Hz), 4.86 (1H, dtd, J = 53.9, 8.9, 3.0 Hz), 4.71-4.63 (1H, m), 2.58 (1H, t, J = 11.2 Hz), 2.48-2.34 (1H, m), 2.36 (1H, t, J = 13.3 Hz), 2.17-2.06 (2H, m), 2.04 (3H, s), 2.02 (3H, s), 1.93-1.76 (3H, m), 1.71-1.39 (7H, m), 1.29-1.17 (2H, m), 1.13-1.05 (1H, m), 1.10 (3H, s), 0.79 (3H, s); $^{13}C\{^1H\}$ NMR (100 MHz, $CDCl_3$): 208.5, 170.7, 170.4, 93.0 (d, J = 184.3 Hz, C15), 78.3 (d, J = 2.6 Hz, C17), 72.5, 54.6, 49.9 (d, J = 18.1 Hz, C14), 48.6, 45.8, 45.6, 44.1 (d, J = 5.2 Hz, C13), 36.8, 36.6, 36.0, 35.8, 35.7, 33.7, 27.0, 21.3, 21.1, 21.0, 13.5, 11.7; ^{19}F NMR (282 MHz, $CDCl_3$): -167.6 (1F, dm, J = 53.3 Hz); υ_{max} (ATR-IR): 1735 (br), 1717 cm^{-1}; HRMS (ESI) m/z $C_{23}H_{35}FO_5Na^+$: calc 431.221209, observed 431.220332.

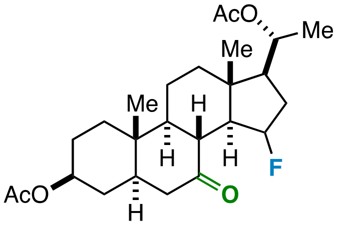

Table 3. Compound 12. The reaction was run according to the general procedure, and both diastereomers were isolated. Regiochemical assignment was made on the basis of 1) chemical shift in the ^{19}F NMR spectrum that indicates a secondary fluoride, 2) $^{2}J_{HF}$-coupling in the ^{1}H and ^{19}F NMR spectra that indicates cyclopentane ring fluorination (>50 Hz), and 3) identification of $^{2}J_{CF}$- and $^{3}J_{CF}$-coupling to distinguishable peaks in the ^{13}C NMR spectrum, i.e. C8, C13, C14, C16, and C17 *vide infra*. Stereochemical assignment for the major diastereomer was made on the basis of X-ray crystallography. Stereochemical assignment for the minor diastereomer was made on the basis of X-ray crystallography.

(*Major Diastereomer*) White solid; m.p. 154-155 °C. ^{1}H NMR (400 MHz, $CDCl_3$): 4.94-4.64 (3H, m), 2.56 (1H, t, J = 11.3 Hz), 2.37 (1H, t, J = 13.1 Hz), 2.17-2.11 (1H, m), 2.04 (3H, s), 2.02 (3H, s), 1.99-1.38 (14H, m), 1.37-1.22 (2H, m), 1.18 (3H, d, J = 6.1 Hz), 1.10 (3H, s), 0.68 (3H, s); ^{13}C{^{1}H} NMR (100 MHz, $CDCl_3$): 208.9, 170.4, 170.2, 94.4 (d, J = 181.7 Hz, C15), 72.5, 71.5 (d, J = 1.5 Hz, C17), 54.8, 54.0 (d, J = 17.3 Hz, C14), 51.6, 51.5, 48.5, 45.9, 45.7, 43.5 (d, J = 5.9 Hz, C13), 38.1, 35.9 (d, J = 23.6 Hz, C16), 35.1, 34.8, 33.7, 27.1, 21.5, 21.4, 19.8, 13.6, 11.6; ^{19}F NMR (282 MHz, $CDCl_3$): -167.4 (1F, dm, J = 55.1 Hz); υ_{max} (ATR-IR): 1729, 1708 cm^{-1}; HRMS (ESI) m/z $C_{25}H_{37}FO_5Na^+$: calc 459.251724, observed 459.252169.

(*Minor Diastereomer*) White solid; m.p. 170-171 °C. ^{1}H NMR (400 MHz, $CDCl_3$): 5.56 (1H, dm, J = 55.9 Hz), 5.02-4.92 (1H, m), 4.75-4.64 (1H, m), 2.74 (1H, t, J = 11.6 Hz), 2.42 (1H, t, J = 12.6 Hz), 2.39-2.22 (1H, m), 2.12-2.07 (1H, m), 2.04 (3H, s), 2.03 (3H, s), 1.97-1.87 (1H, m), 1.85-1.77 (2H, m), 1.73-1.41 (9H, m), 1.36-1.27 (1H, m), 1.23-1.17 (1H, m), 1.19 (3H, d, J = 6.1 Hz), 1.13 (3H, s), 1.10-1.04 (1H, m), 0.85 (3H, d, J = 2.1 Hz); ^{13}C{^{1}H} NMR (100 MHz, $CDCl_3$): 210.6, 170.5, 170.2, 93.3 (d, J = 176.9, C15), 72.6, 72.0, 55.0, 54.0, 53.3 (d, J = 20.3 Hz, C14), 46.4, 45.5, 44.9 (d, J = 3.7 Hz, C13), 41.9 (d, J = 1.8 Hz, C8), 39.1, 36.0, 35.7, 35.6 (d, J = 21.0 Hz, C16), 33.8, 27.0, 21.6, 21.4, 21.3, 19.8, 14.4 (d, J = 4.4 Hz, C18), 11.6; ^{19}F NMR (282 MHz, $CDCl_3$): -179.1 (1F, dddd, J = 57.9, 38.4, 37.3, 20.7 Hz).

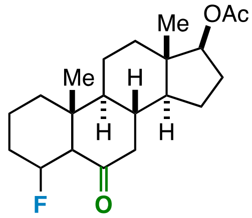

Table 3. Compound 13. The reaction was run according to the general procedure, and the major diastereomer was isolated. Regiochemical assignment was made on the basis of 1) chemical shift in the ^{19}F NMR spectrum that indicates a secondary fluoride on a cyclohexane ring and 2) identification of $^2J_{CF}$- and $^3J_{CF}$-coupling to distinguishable peaks in the ^{13}C NMR spectrum, i.e. C2, C3, C5, and C10 *vide infra*. Stereochemical assignment was made on the basis of 1) chemical shift and splitting in the ^{19}F NMR spectrum that indicates F_{eq} on a cyclohexane ring and 2) identification of the two distinct *trans* diaxial $^3J_{HH}$-coupling constants (t, 10.8 Hz) in the ^{1}H NMR spectrum.

White solid; m.p. 189-192 °C. ^{1}H NMR (400 MHz, $CDCl_3$): 4.94 (1H, dtd, J = 48.2, 10.8, 5.5 Hz), 4.64 (1H, dd, J = 9.2, 7.8 Hz), 2.38 (1H, t, J = 10.0 Hz), 2.34 (1H, dd, J = 12.3, 4.5 Hz), 2.23-2.13 (2H, m), 2.07 (1H, t, J = 12.2 Hz), 2.04 (3H, s), 1.90-1.58 (6H, m), 1.55-1.14 (9H, m), 0.78 (3H, s), 0.73 (3H, s); ^{13}C{^{1}H} NMR (100 MHz, $CDCl_3$): 208.4, 171.1, 86.8 (d, J = 170.6 Hz, C4), 82.2, 63.4 (d, J = 15.8 Hz, C5), 54.4, 51.1, 46.7, 44.7 (d, J = 7.0 Hz, C10), 43.1, 38.6, 36.9 (d, J = 2.2 Hz, C1), 36.4, 31.5 (d, J = 18.8 Hz, C3), 27.3, 23.2, 21.1, 20.8, 19.3 (d, J = 12.2 Hz, C2), 14.0, 12.0; ^{19}F NMR (282 MHz, $CDCl_3$): -176.8 (1F, dm, J = 47.6 Hz); υ_{max} (ATR-IR): 1736, 1716 cm^{-1}; HRMS (ESI) m/z $C_{21}H_{31}FO_3Na^+$: calc 373.214944, observed 373.215192.

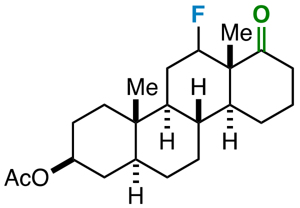

Table 3. Compound 14. The reaction was run according to the general procedure, and both diastereomers were isolated. Regiochemical assignment was made on the basis of 1) chemical shift in the ^{19}F NMR spectrum that indicates a secondary fluoride on a cyclohexane ring, 2) identification of $^4J_{HF}$-coupling to the distinguishable C18 Me hydrogen atoms in the ^{1}H NMR spectrum, and 3) identification of $^2J_{CF}$- and $^3J_{CF}$-coupling to distinguishable peaks in the ^{13}C NMR spectrum, i.e. C11, C13, C17a, and C18 *vide infra*. Stereochemical assignment for the major diastereomer was made on the basis of 1) chemical shift and splitting in the ^{19}F NMR spectrum that indicates F_{ax} on a cyclohexane ring and 2) identification of the distinct *trans* diaxial $^3J_{FH}$-coupling constant (46.5 Hz) in the ^{19}F NMR spectrum. Stereochemical assignment for the minor diastereomer was made on the basis of 1) chemical shift, splitting, and two "gauche" $^3J_{FH}$-coupling constants (t, 8.0 Hz) in the ^{19}F NMR spectrum that indicates F_{eq} on a cyclohexane ring and 2)

identification of the distinct *trans* diaxial ${}^3J_{HH}$-coupling constant (11.7 Hz) in the ^{1}H NMR spectrum.

(*Major Diastereomer*) White solid; m.p. 130-132 °C. ^{1}H NMR (400 MHz, $CDCl_3$): 5.08 (1H, dm, J = 47.1 Hz), 4.72-4.64 (1H, m), 2.50-2.41 (1H, m), 2.36-2.30 (1H, m), 2.01 (3H, s), 2.00-1.79 (6H, m), 1.73-1.61 (3H, m), 1.56-1.16 (9H, m), 1.10-1.02 (1H, m), 1.00 (3H, d, J = 1.1 Hz), 0.98-0.86 (1H, m), 0.81 (3H, s); $^{13}C\{^1H\}$ NMR (100 MHz, $CDCl_3$): 212.4, 170.6, 91.2 (d, J = 172.9 Hz, C12), 73.4, 51.4 (d, J = 18.4 Hz, C13), 46.5, 44.0, 42.1 (d, J = 1.5 Hz), 37.1, 36.1, 35.2, 34.5, 33.8, 30.7, 28.4, 27.2, 25.5 (d, J = 21.4 Hz, C11), 23.1, 21.8, 21.4, 16.4 (d, J = 7.0 Hz, C18), 11.9; ^{19}F NMR (282 MHz, $CDCl_3$): -184.7 (1F, td, J = 46.5, 10.3 Hz); υ_{max} (ATR-IR): 1732, 1712 cm^{-1}; HRMS (ESI) m/z $C_{22}H_{33}FO_3Na^+$: calc 387.230594, observed 387.230475.

(*Minor Diastereomer*) Viscous oil. ^{1}H NMR (400 MHz, $CDCl_3$): 4.91 (1H, ddd, J = 46.8, 11.7, 5.3 Hz), 4.71-4.63 (1H, m), 2.76-2.67 (1H, m), 2.22-2.18 (1H, m), 2.13-2.07 (1H, m), 2.05-1.96 (1H, m), 2.02 (3H, s), 1.87-1.79 (3H, m), 1.75 (1H, dt, J = 13.0, 3.5 Hz), 1.67-1.61 (1H, m), 1.58-1.22 (8H, m), 1.21 (3H, d, J = 1.3 Hz), 1.17-1.08 (2H, m), 1.06-0.98 (1H, m), 0.90-0.73 (2H, m), 0.83 (3H, s); $^{13}C\{^1H\}$ NMR (100 MHz, $CDCl_3$): 213.3 (d, J = 0.7 Hz, C17a), 170.6, 91.5 (d, J = 179.0 Hz, C12), 73.3, 53.1 (d, J = 15.5 Hz, C13), 50.8 (d, J = 4.1 Hz), 50.2 (d, J = 10.3 Hz), 43.9, 37.3, 36.4, 35.5 (d, J = 1.1 Hz), 34.3 (d, J = 1.5 Hz), 33.7, 30.9, 28.2, 27.3, 26.8, 26.7 (d, J = 18.4 Hz), 22.4 (d, J = 2.6 Hz), 21.4, 12.1, 11.1 (d, J = 5.2 Hz, C18); ^{19}F NMR (282 MHz, $CDCl_3$): -179.8 (1F, dt, J = 46.5, 8.0 Hz).

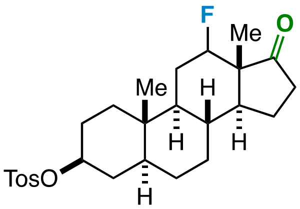

Table 3. Compound 15. The reaction was run according to the general procedure, and the major diastereomer was isolated. Regiochemical assignment was made on the basis of 1) chemical shift in the ^{19}F NMR spectrum that indicates a secondary fluoride on a cyclohexane ring and 2) identification of ${}^2J_{CF}$- and ${}^3J_{CF}$-coupling to distinguishable peaks in the ^{13}C NMR spectrum, i.e. C11, C13, and C18 *vide infra*. Stereochemical assignment was made on the basis of 1) chemical shift and splitting in the ^{19}F NMR spectrum that indicates F_{ax} on a cyclohexane ring and 2) identification of the distinct *trans* diaxial ${}^3J_{FH}$-coupling constant (47.6 Hz) in the ^{19}F NMR spectrum.

White solid; m.p. 146-147 °C. ^{1}H NMR (400 MHz, $CDCl_3$): 7.79 (2H, dm, J = 8.3 Hz), 7.33 (2H, dm, J = 8.6 Hz), 4.87 (1H, dm, J = 49.4 Hz), 4.39 (1H, m), 2.46-2.36 (1H, m), 2.44 (3H, s), 2.18-2.06 (1H, m), 2.02-0.83 (18H, m), 0.81-0.79 (6H, m); $^{13}C\{^1H\}$ NMR (100 MHz, $CDCl_3$): 216.4, 144.4, 134.6, 129.7, 127.6, 90.3 (d, J = 173.6 Hz, C12), 81.8, 51.4 (d, J = 20.3 Hz, C13), 48.3, 44.7, 43.8, 36.4, 36.3, 35.0, 34.8, 34.4, 30.4, 28.1, 28.0,

26.4 (d, J = 21.8 Hz, C11), 21.6, 21.0, 13.3 (d, J = 7.4 Hz, C18), 11.9; ^{19}F NMR (282 MHz, $CDCl_3$): -187.5 (1F, td, J = 47.6, 11.5 Hz); υ_{max} (ATR-IR): 1743 cm^{-1}; HRMS (ESI) m/z $C_{26}H_{35}FO_4SNa^+$: calc 485.213230, observed 485.212970.

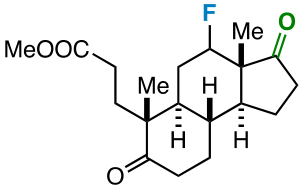

Table 3. Compound 16. The reaction was run according to the general procedure, and the major diastereomer was isolated. Regiochemical assignment was made on the basis of 1) chemical shift in the ^{19}F NMR spectrum that indicates a secondary fluoride on a cyclohexane ring and 2) identification of $^2J_{CF}$- and $^3J_{CF}$-coupling to distinguishable peaks in the ^{13}C NMR spectrum, i.e. C11, C13, and C18 *vide infra*. Stereochemical assignment was made on the basis of 1) chemical shift and splitting in the ^{19}F NMR spectrum that indicates F_{ax} on a cyclohexane ring and 2) identification of a *trans* diaxial $^3J_{FH}$-coupling constant in the ^{19}F NMR spectrum.

Viscous oil. ^{1}H NMR (400 MHz, $CDCl_3$): 4.94 (1H, dm, J = 48.1 Hz), 3.66 (3H, s), 2.62-2.44 (2H, m), 2.40-2.27 (2H, m), 2.25-1.98 (7H, m), 1.92-1.85 (1H, m), 1.80-1.53 (4H, m), 1.43-1.31 (1H, m), 1.13 (3H, s), 0.90 (3H, d, J = 0.9 Hz); ^{13}C{^{1}H} NMR (100 MHz, $CDCl_3$): 215.4 (d, J = 1.1 Hz), 212.9, 174.0, 89.6 (d, J = 175.1 Hz), 51.6, 51.3 (d, J = 19.9 Hz), 49.9, 43.3 (d, J = 1.5 Hz), 42.1, 37.6, 36.2, 34.0, 29.6, 29.3, 28.9, 26.6 (d, J = 22.1 Hz), 21.1, 20.3, 13.4 (d, J = 7.4 Hz); ^{19}F NMR (282 MHz, $CDCl_3$): -186.5 (1F, m); υ_{max} (ATR-IR): 1739, 1733, 1706 cm^{-1}; HRMS (ESI) m/z $C_{19}H_{27}FO_4Na^+$: calc 361.178559, observed 361.178146.

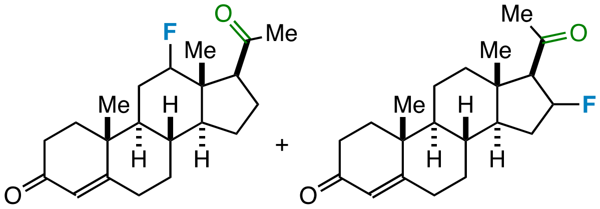

Scheme 1. Compound 17 and 18. The reaction was run according to the general procedure, and the product was isolated as a mixture of regioisomers. Regio- and stereochemical assignments were confirmed by X-ray crystallography.

^{1}H NMR (400 MHz, $CDCl_3$): 5.74 (1H, s), 5.66-4.81 (1H, m), 3.10-2.72 (1H, m), 2.47-2.24 (4H, m), 2.20-2.14 (3H, m), 2.10-1.52 (10H, m), 1.50-1.23 (2H, m), 1.17 (3H, s), 1.15-1.02 (1H, m), 0.71-0.64 (3H, m); ^{13}C{^{1}H} NMR (100 MHz, $CDCl_3$): 209.4, 206.8, 199.6, 199.5, 170.6, 170.3, 124.54, 124.47, 95.1 (d, J = 175.8 Hz), 92.8 (d, J = 176.2 Hz), 71.5, 71.3, 55.30, 55.26, 53.70, 53.69, 48.2, 47.8, 47.3, 47.1, 45.3, 45.2, 38.9, 38.8, 38.3,

35.9, 35.8, 35.5, 35.2, 34.2, 34.1, 33.7, 33.5, 32.94, 32.89, 32.0, 31.9, 31.8, 31.3, 26.9, 26.7, 23.8, 22.6, 21.0, 17.6, 17.5, 14.8, 13.22, 13.16; ^{19}F NMR (282 MHz, $CDCl_3$): -172.0 (1F, dm, J = 53.3 Hz), -192.2 (1F, ddd, J = 48.2, 47.0, 12.1 Hz).

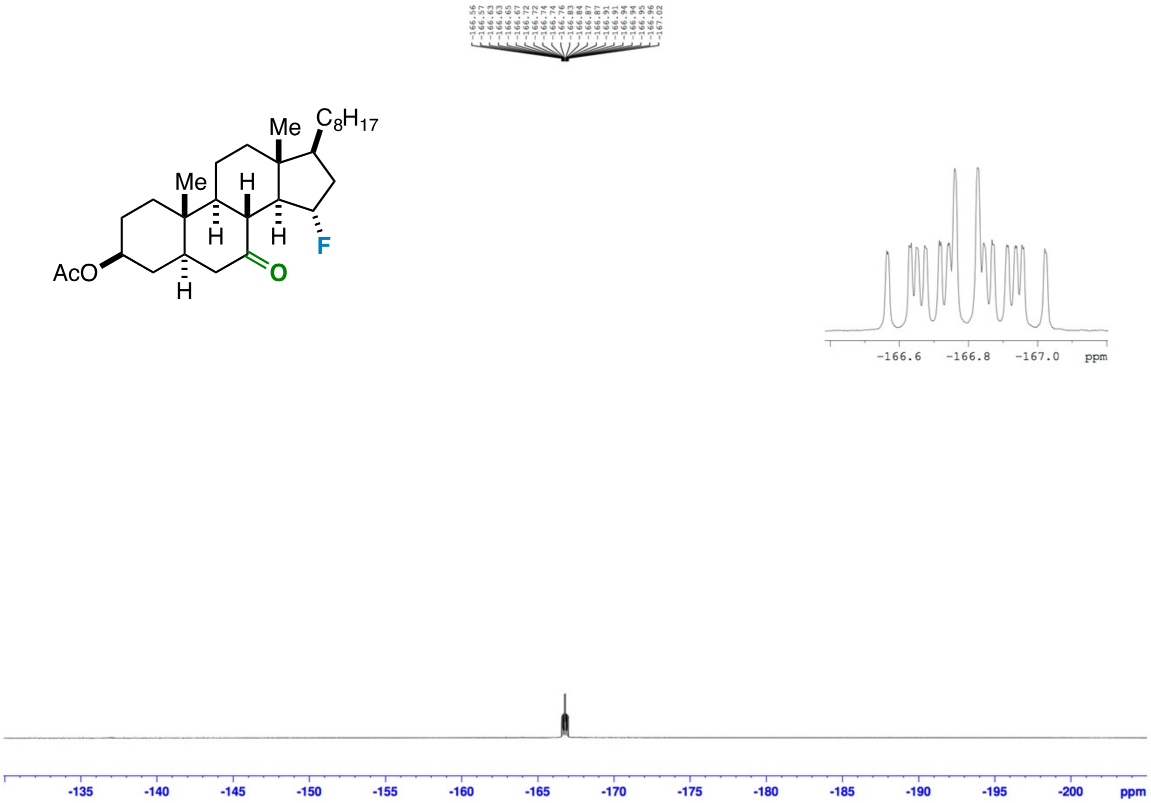

Fig. S1. ^{19}F NMR spectrum ($CDCl_3$, 282 MHz) of compound **2** (major diastereomer).

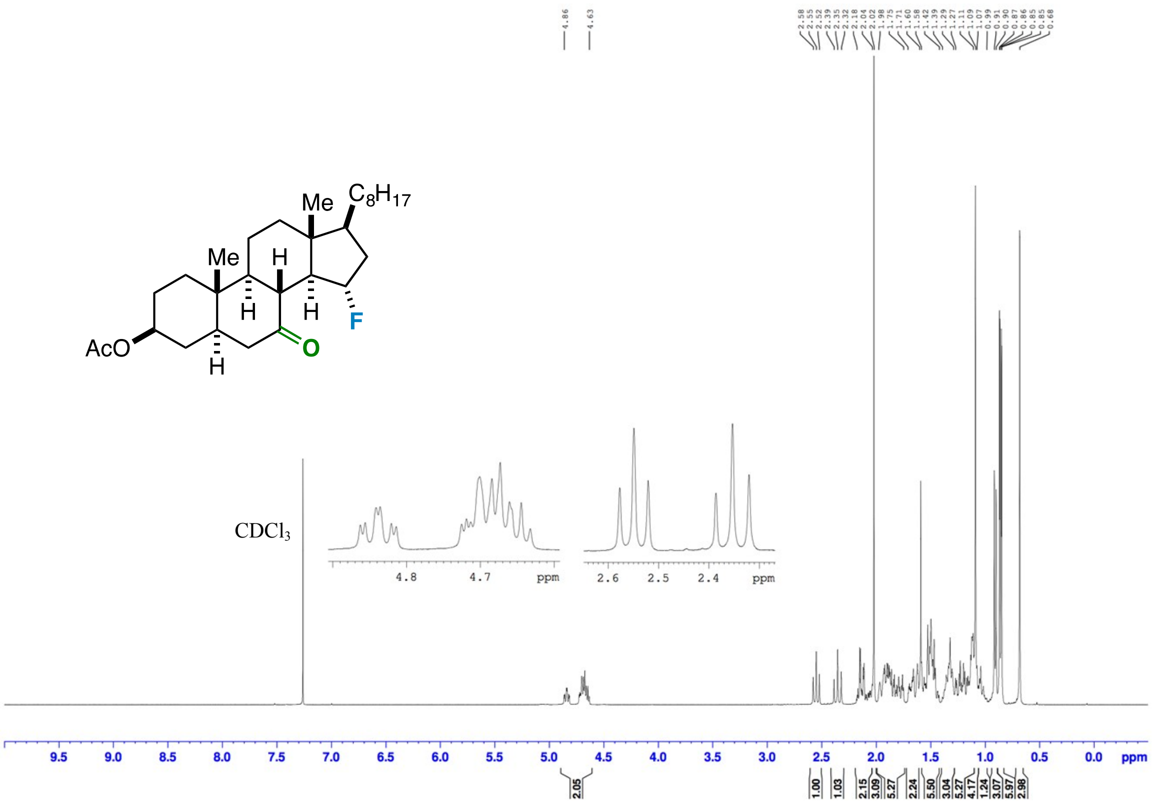

Fig. S2. ^{1}H NMR spectrum ($CDCl_3$, 400 MHz) of compound **2** (major diastereomer).

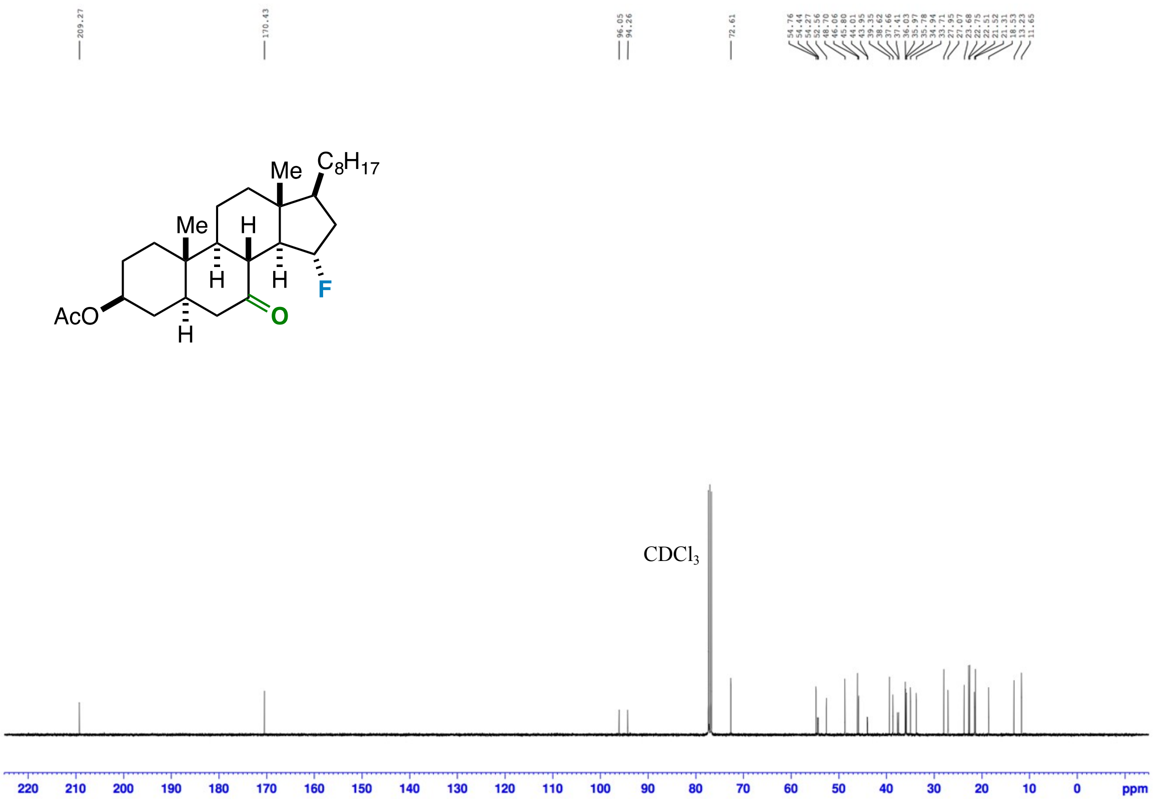

Fig. S3. ^{13}C NMR spectrum ($CDCl_3$, 100 MHz) of compound **2** (major diastereomer).

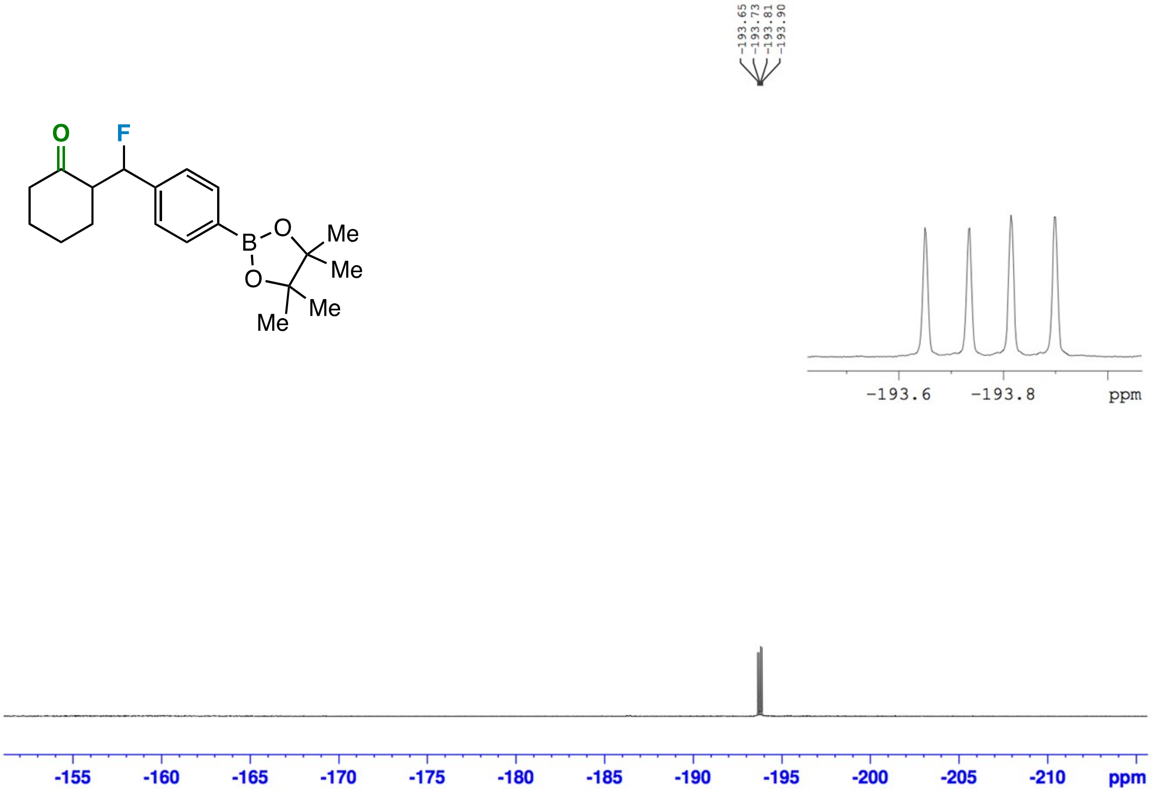

Fig. S4. ^{19}F NMR spectrum ($CDCl_3$, 282 MHz) of compound **4** (minor diastereomer).

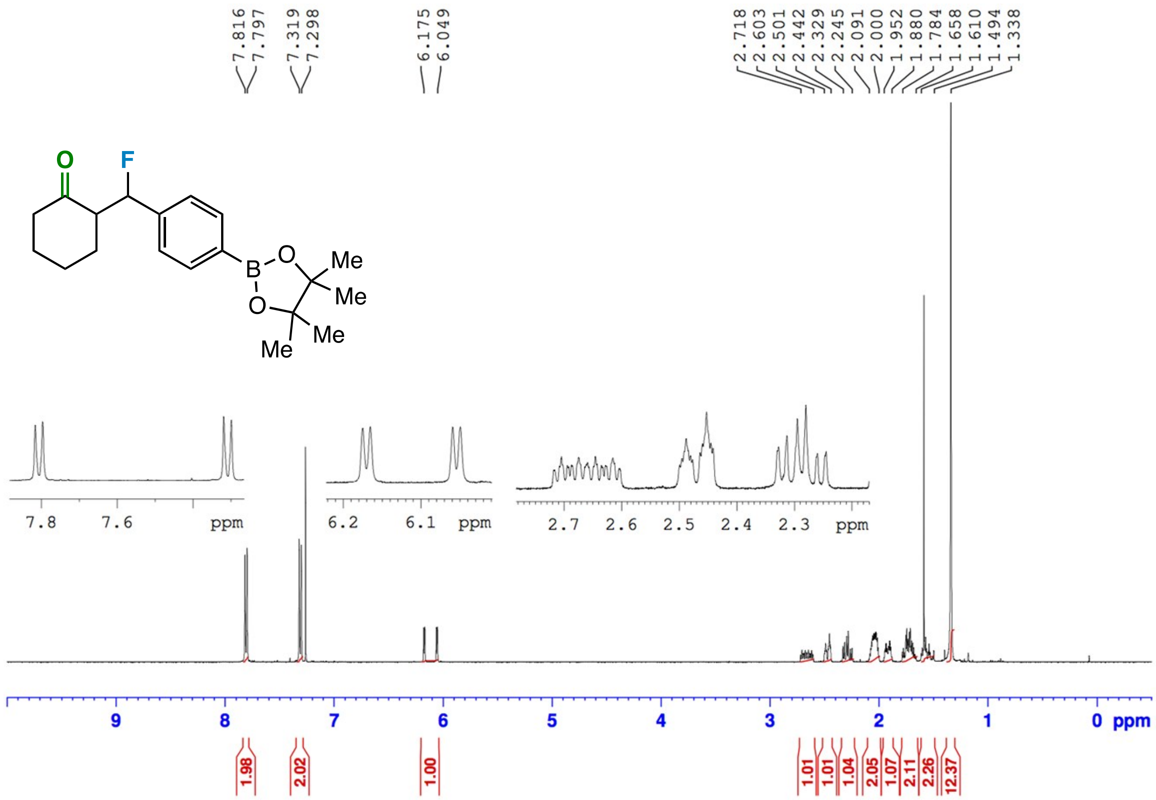

Fig. S5. ^{1}H NMR spectrum ($CDCl_3$, 400 MHz) of compound **4** (minor diastereomer).

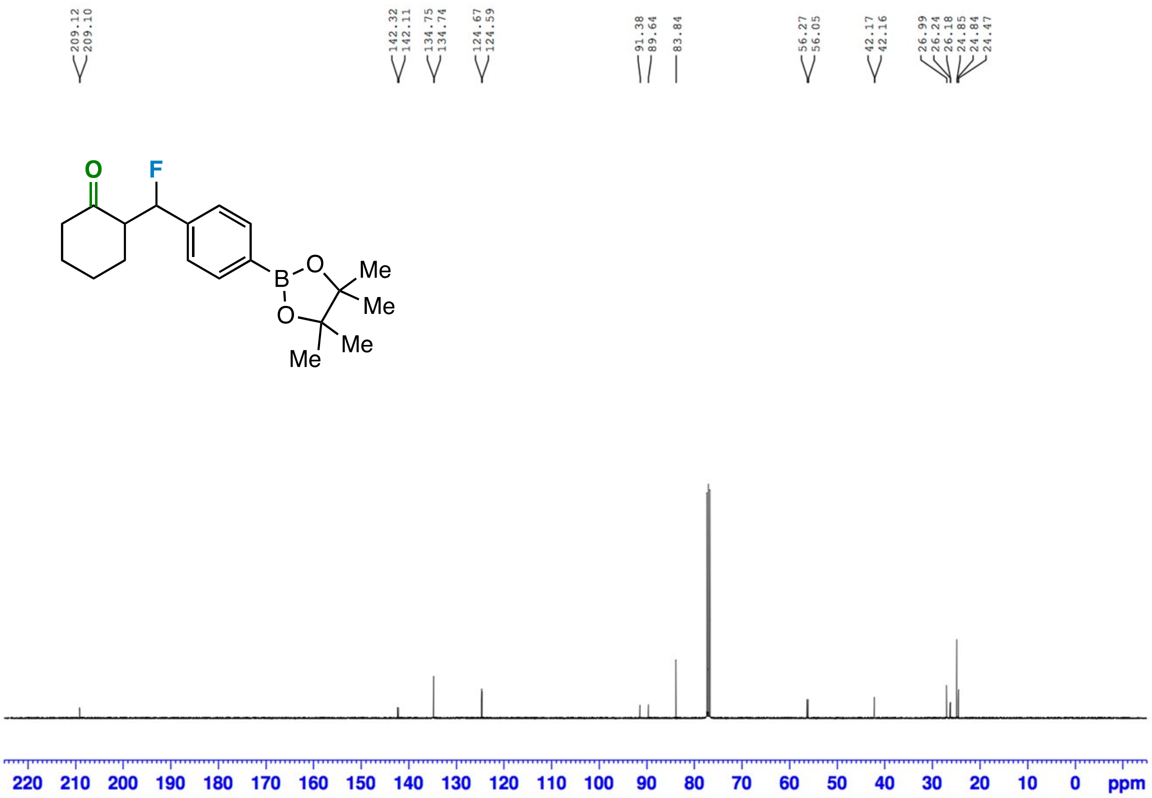

Fig. S6. ^{13}C NMR spectrum ($CDCl_3$, 100 MHz) of compound **4** (minor diastereomer).

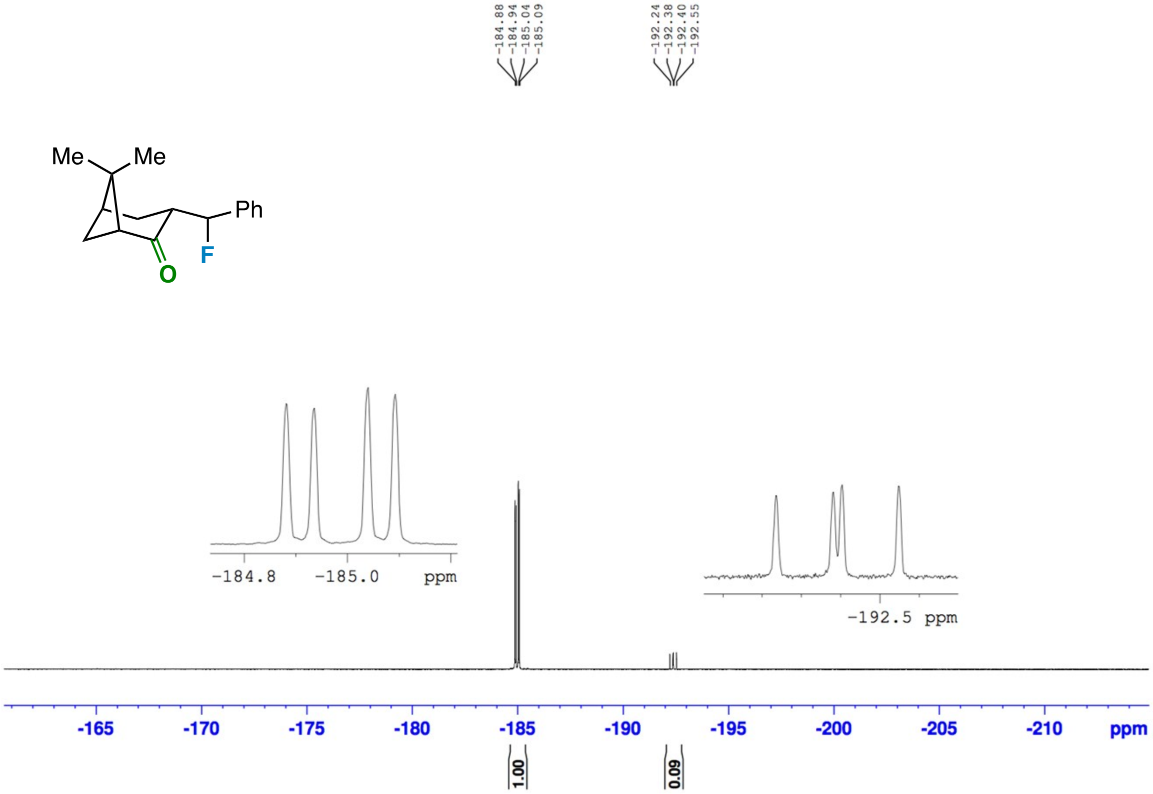

Fig. S7. ^{19}F NMR spectrum ($CDCl_3$, 282 MHz) of compound **5** (mixture of diastereomers).

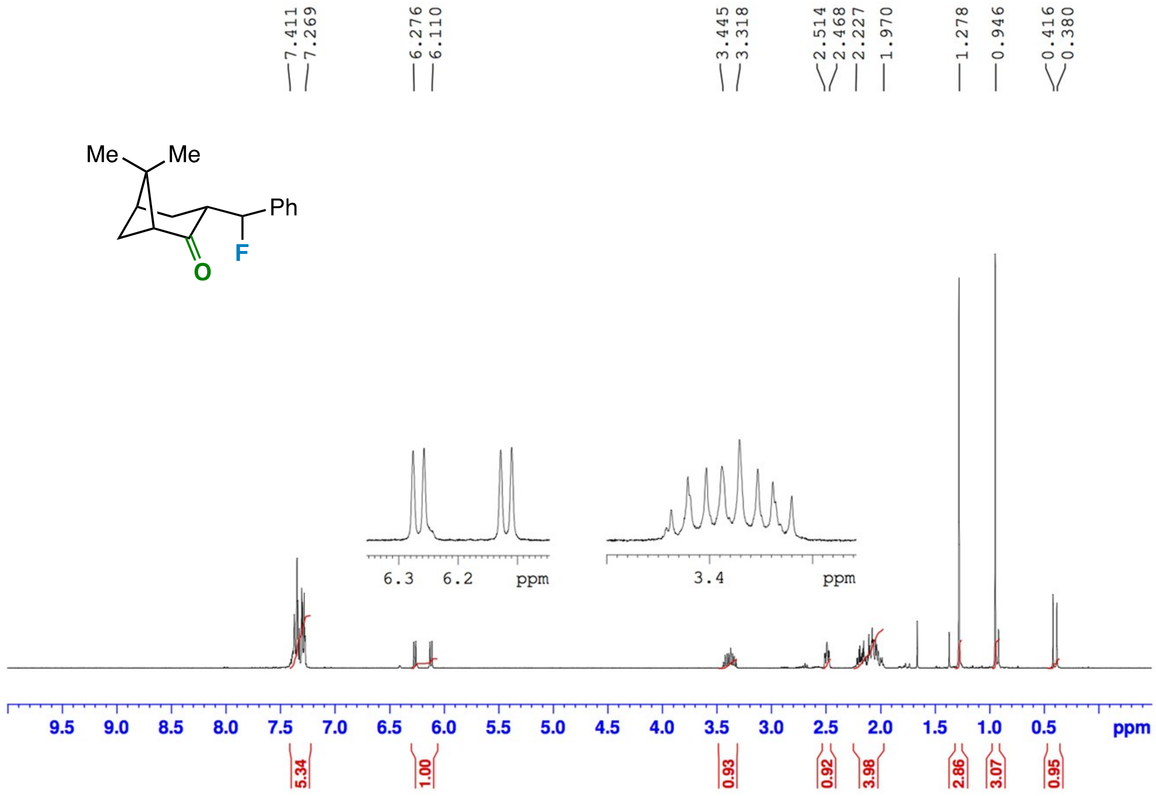

Fig. S8. ^{1}H NMR spectrum ($CDCl_3$, 400 MHz) of compound **5** (mixture of diastereomers).

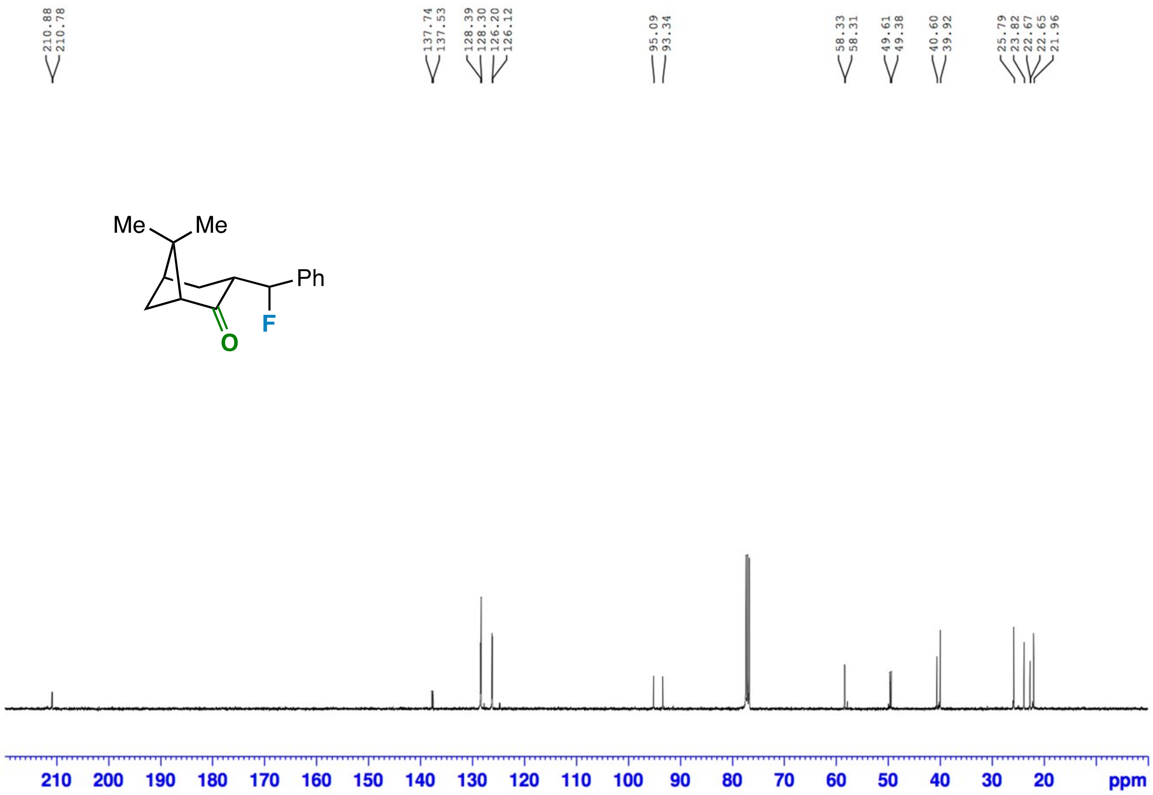

Fig. S9. ^{13}C NMR spectrum ($CDCl_3$, 100 MHz) of compound **5** (mixture of diastereomers).

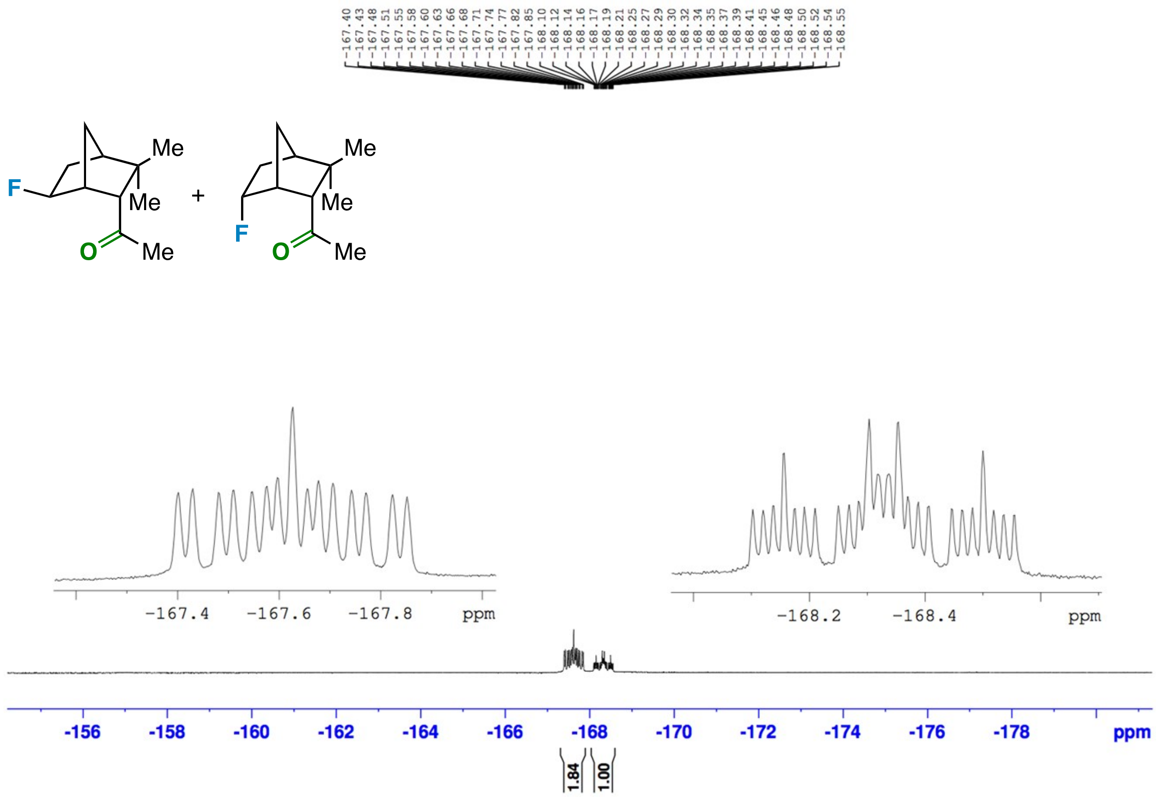

Fig. S10. ^{19}F NMR spectrum ($CDCl_3$, 282 MHz) of compound **6** (mixture of diastereomers).

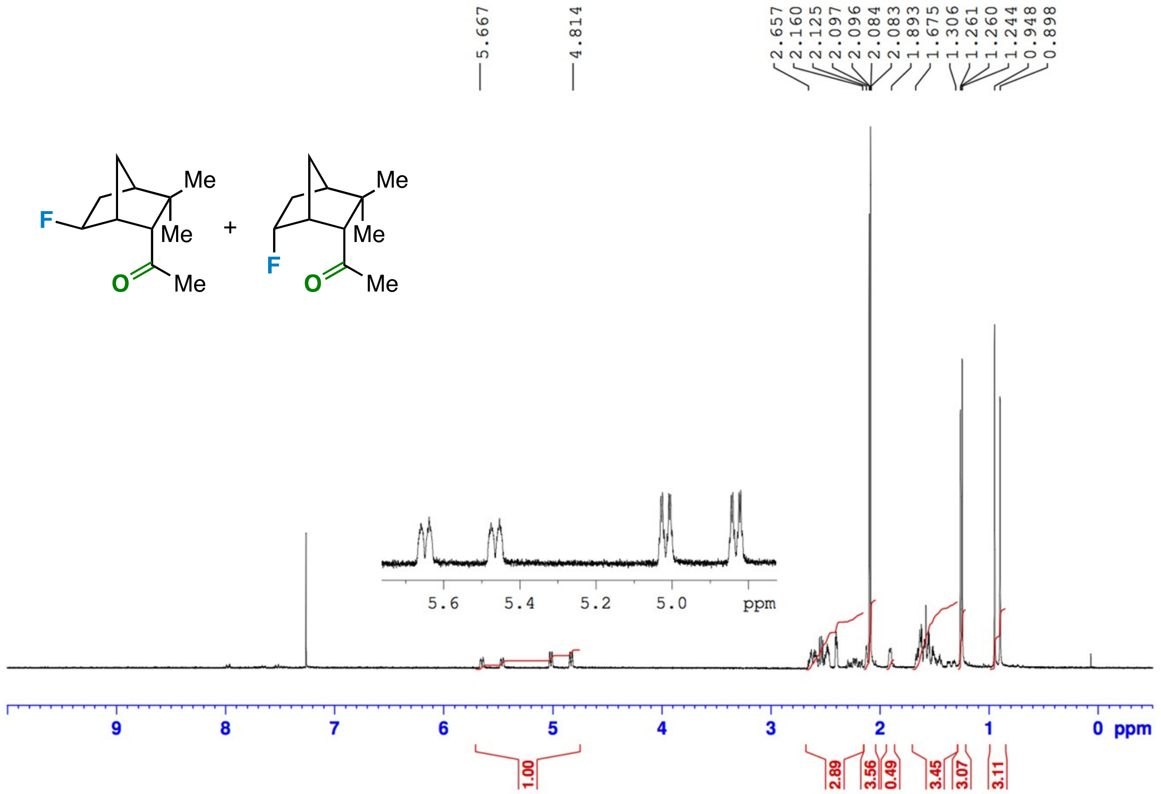

Fig. S11. ^{1}H NMR spectrum ($CDCl_3$, 400 MHz) of compound **6** (mixture of diastereomers).

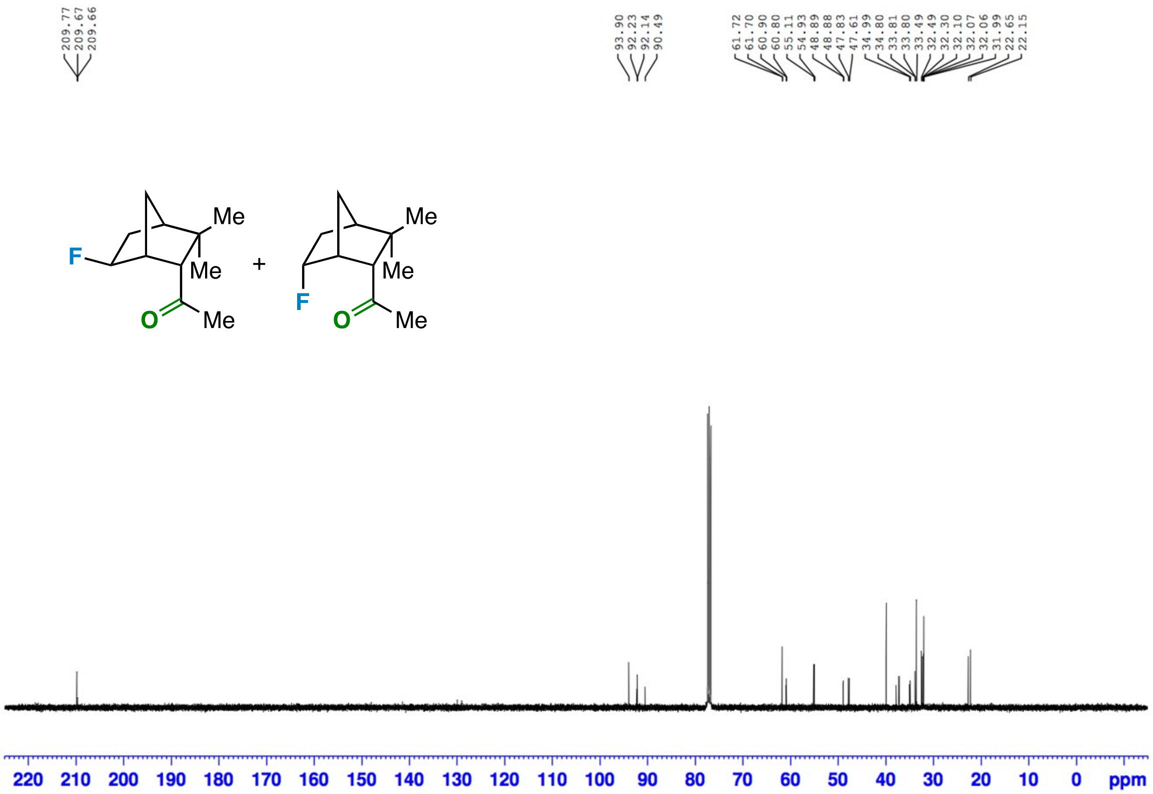

Fig. S12. ^{13}C NMR spectrum ($CDCl_3$, 100 MHz) of compound **6** (mixture of diastereomers).

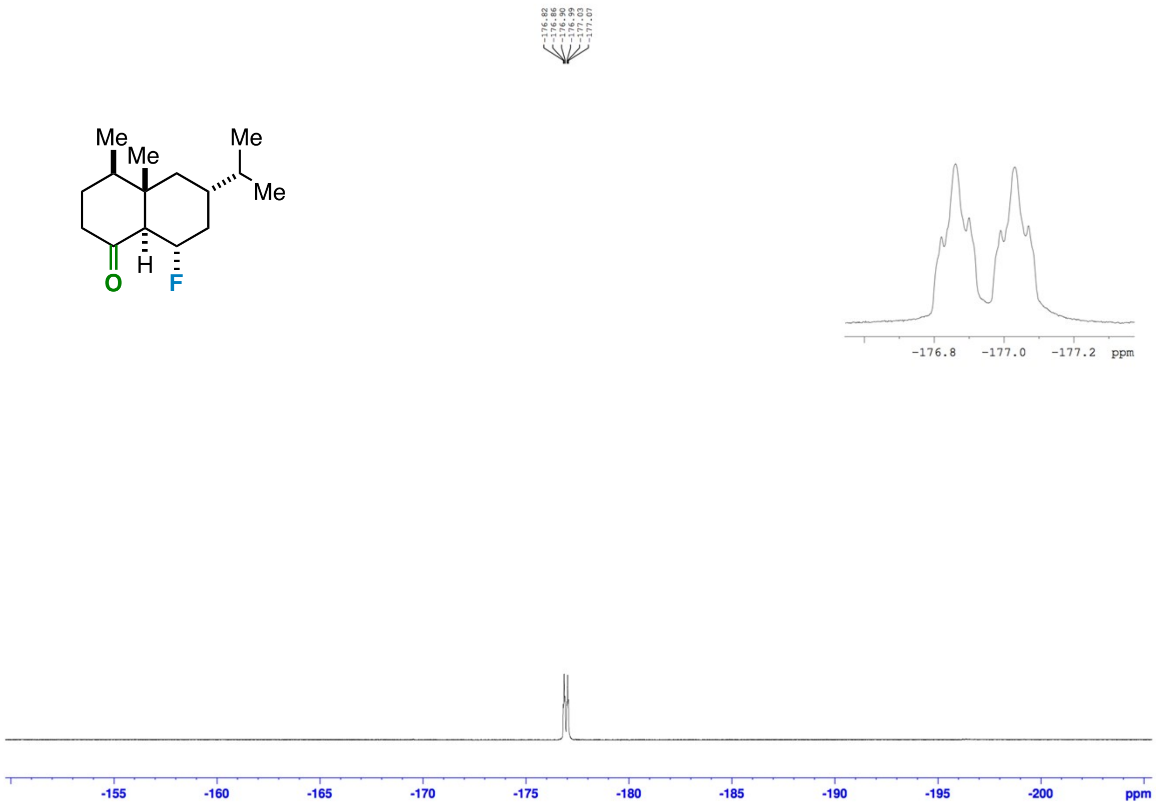

Fig. S13. ^{19}F NMR spectrum ($CDCl_3$, 282 MHz) of compound **7** (major diastereomer).

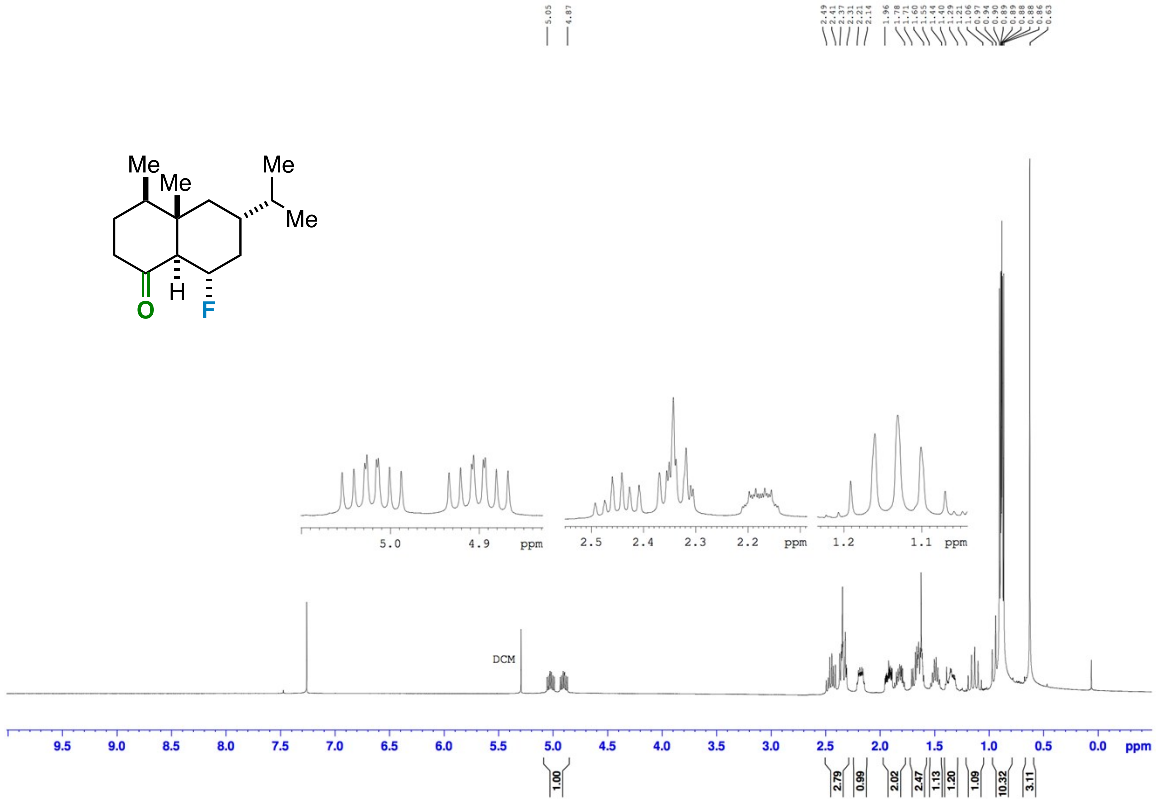

Fig. S14. ^{1}H NMR spectrum ($CDCl_3$, 400 MHz) of compound **7** (major diastereomer).

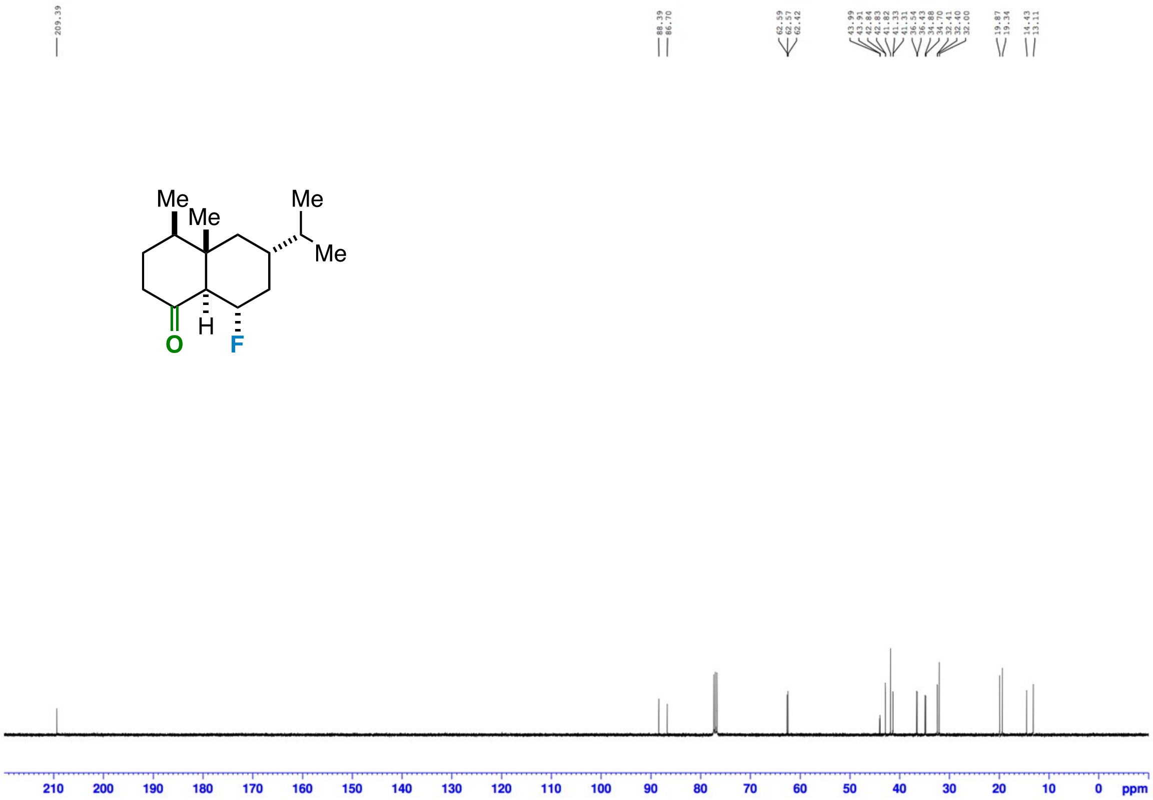

Fig. S15. ^{13}C NMR spectrum ($CDCl_3$, 100 MHz) of compound **7** (major diastereomer).

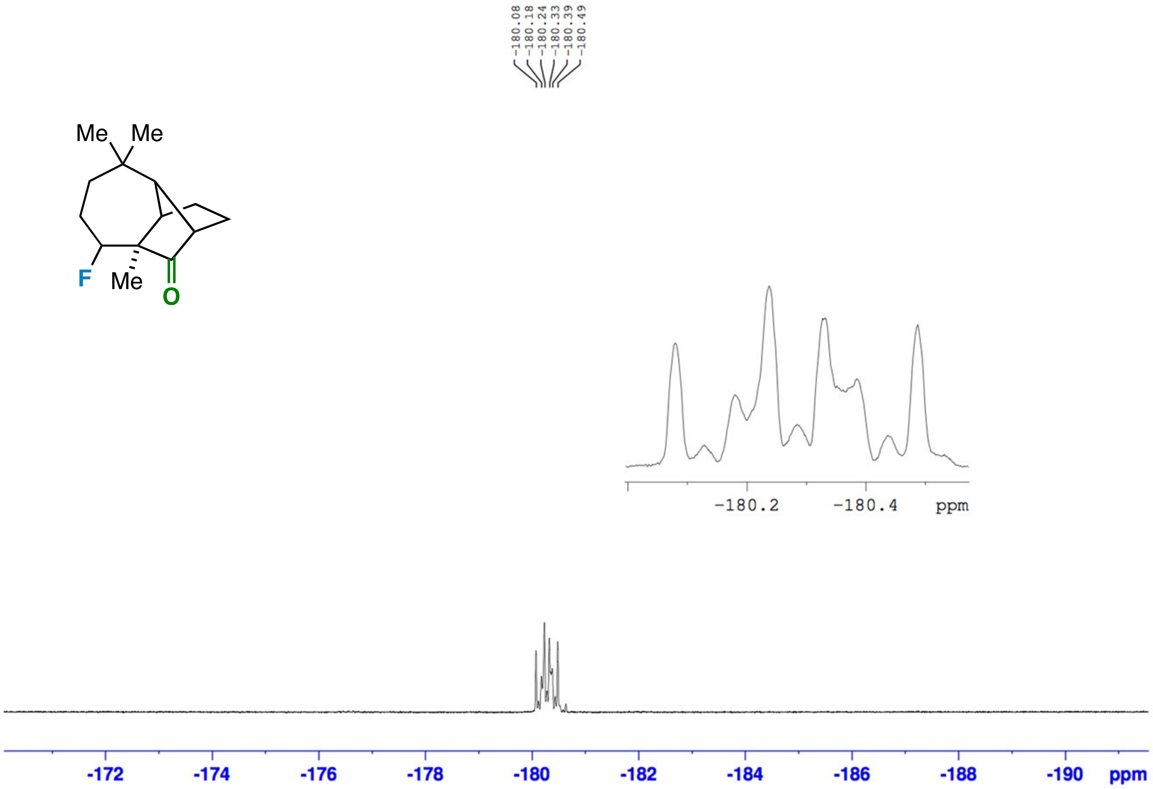

Fig. S16. ^{19}F NMR spectrum ($CDCl_3$, 282 MHz) of compound **8** (major diastereomer).

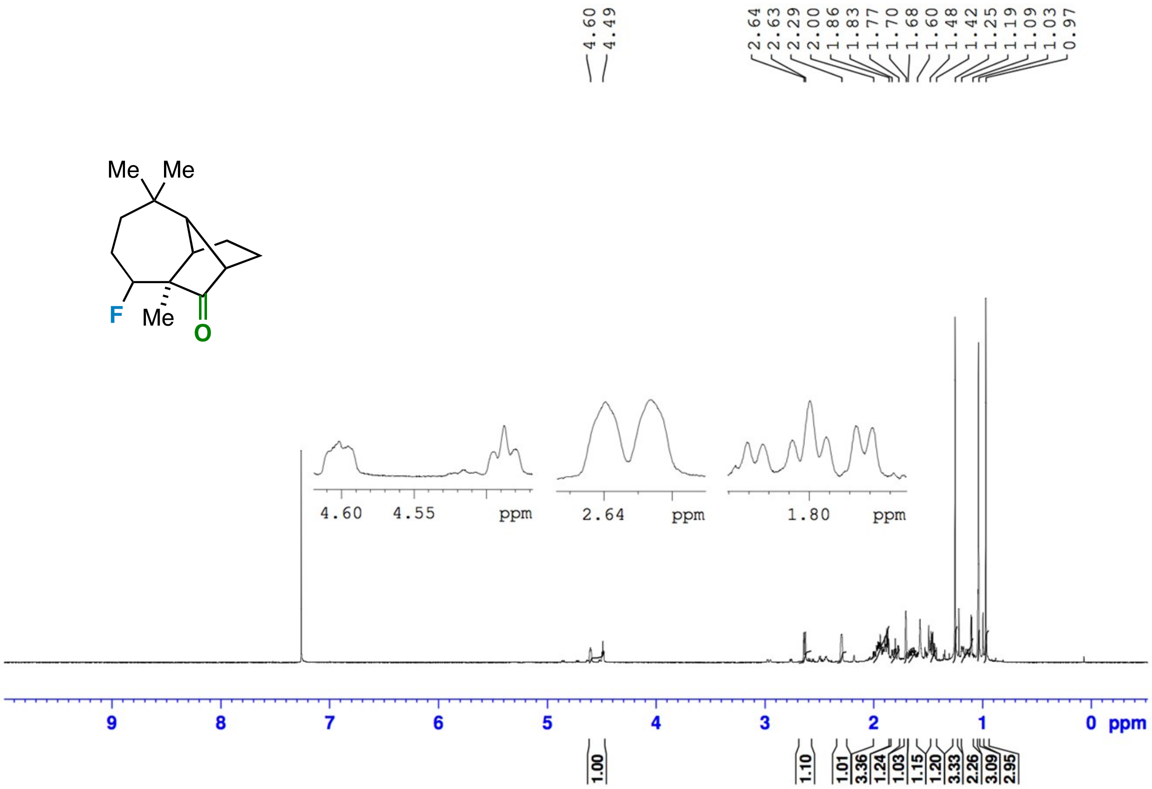

Fig. S17. ^{1}H NMR spectrum ($CDCl_3$, 400 MHz) of compound **8** (major diastereomer).

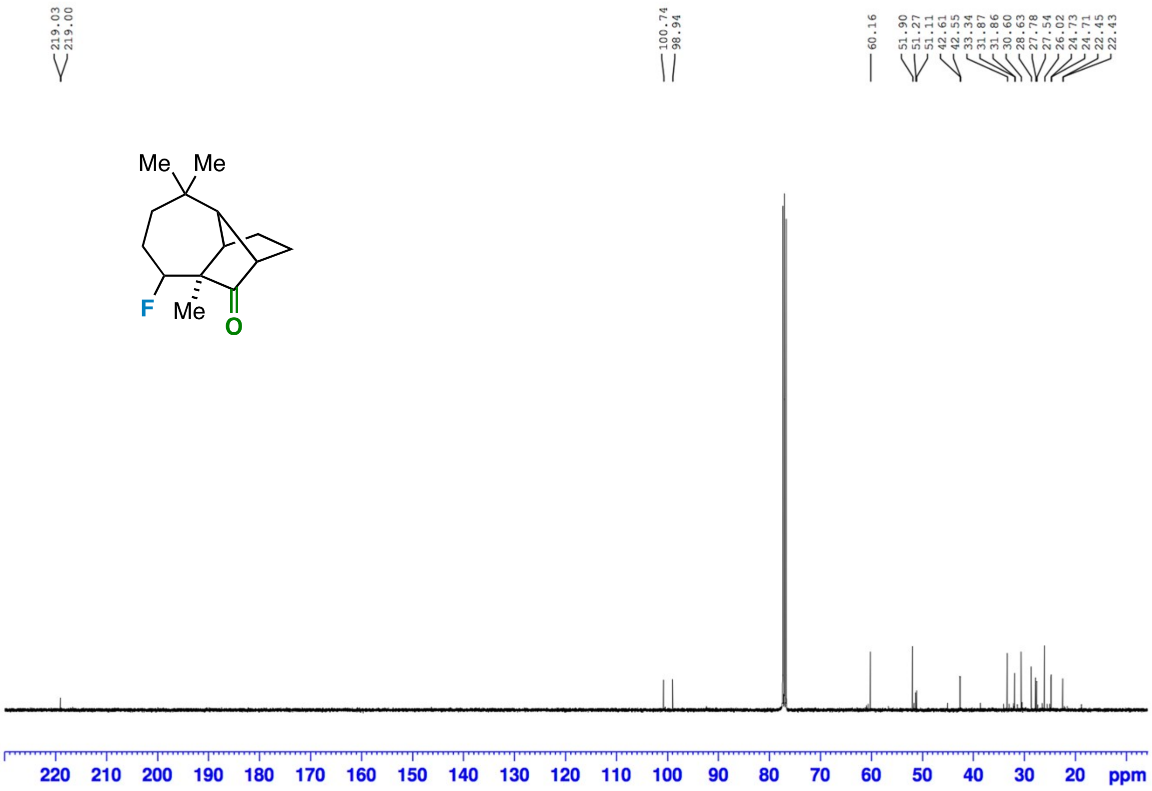

Fig. S18. ^{13}C NMR spectrum ($CDCl_3$, 100 MHz) of compound **8** (major diastereomer).

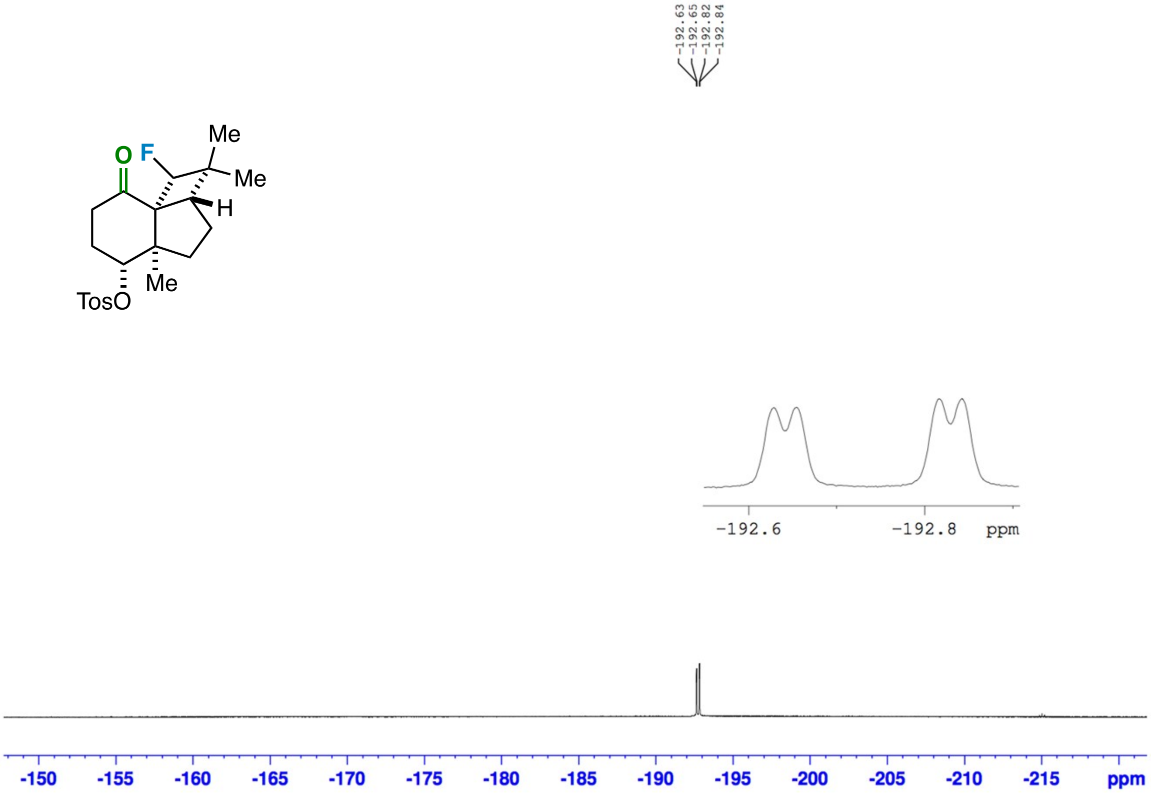

Fig. S19. ^{19}F NMR spectrum ($CDCl_3$, 282 MHz) of compound **9** (major diastereomer).

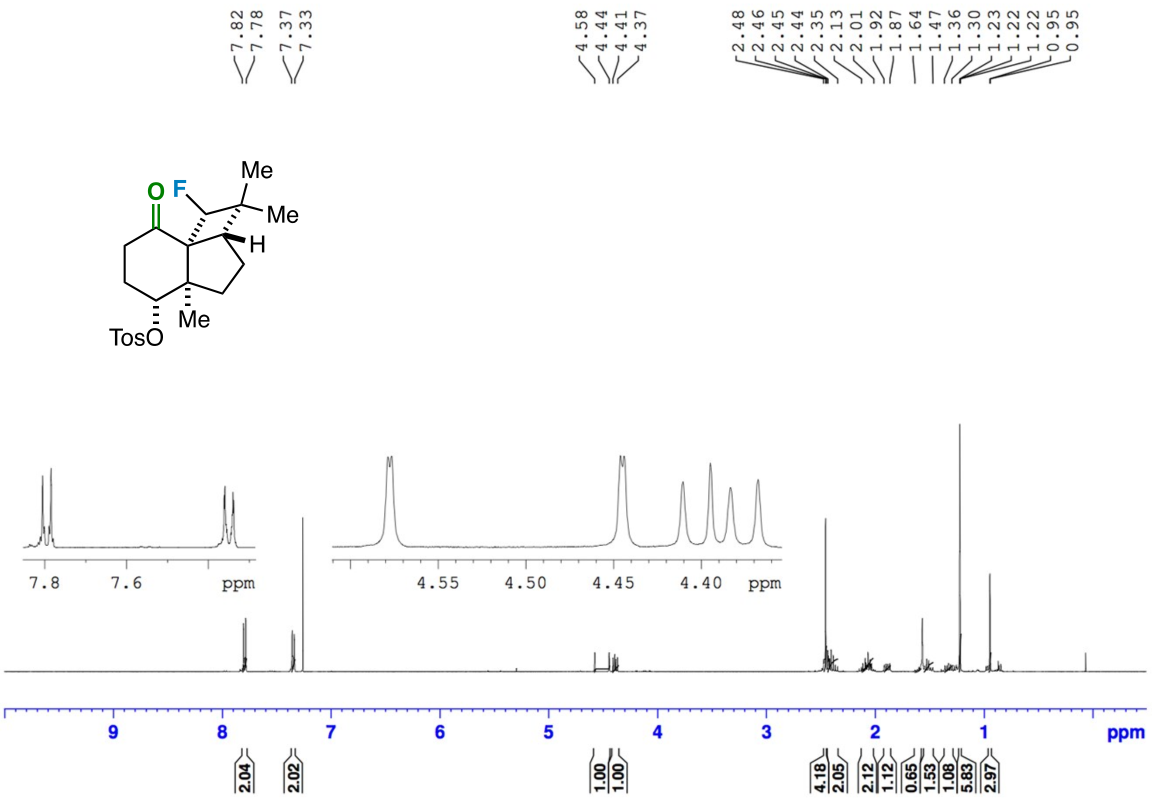

Fig. S20. ^{1}H NMR spectrum ($CDCl_3$, 400 MHz) of compound **9** (major diastereomer).

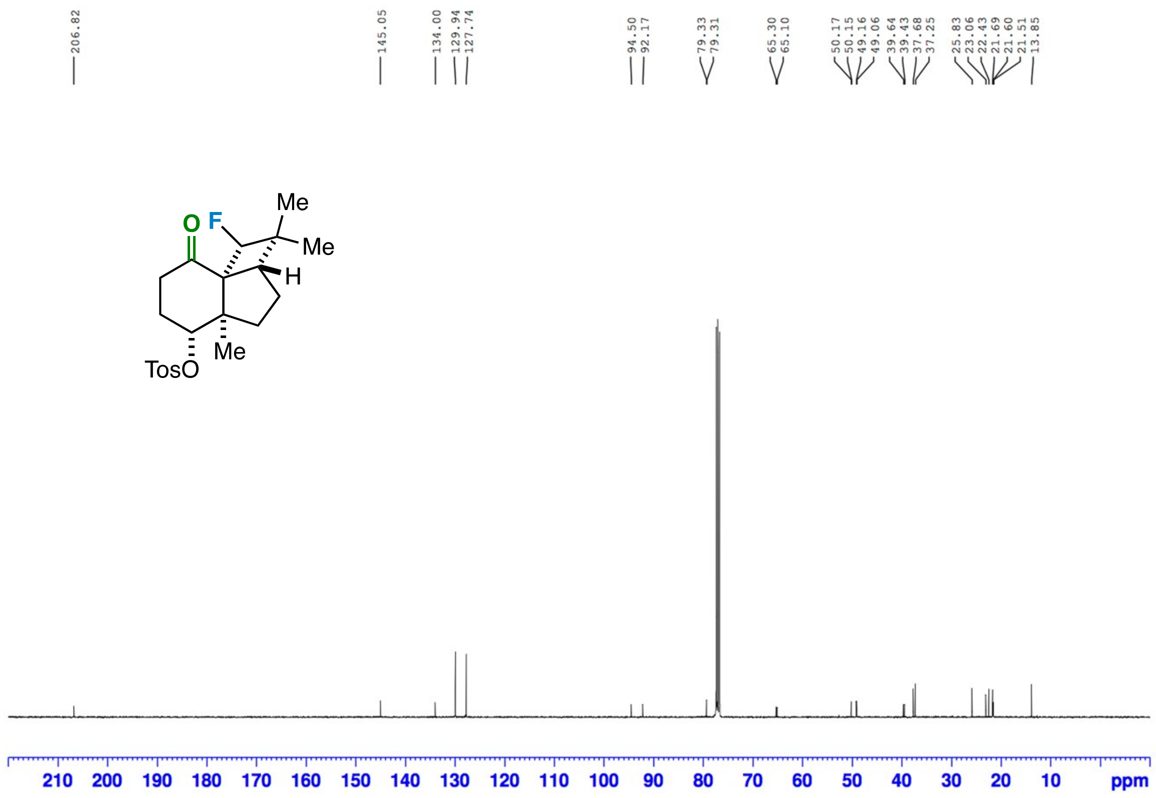

Fig. S21. ^{13}C NMR spectrum ($CDCl_3$, 100 MHz) of compound **9** (major diastereomer).

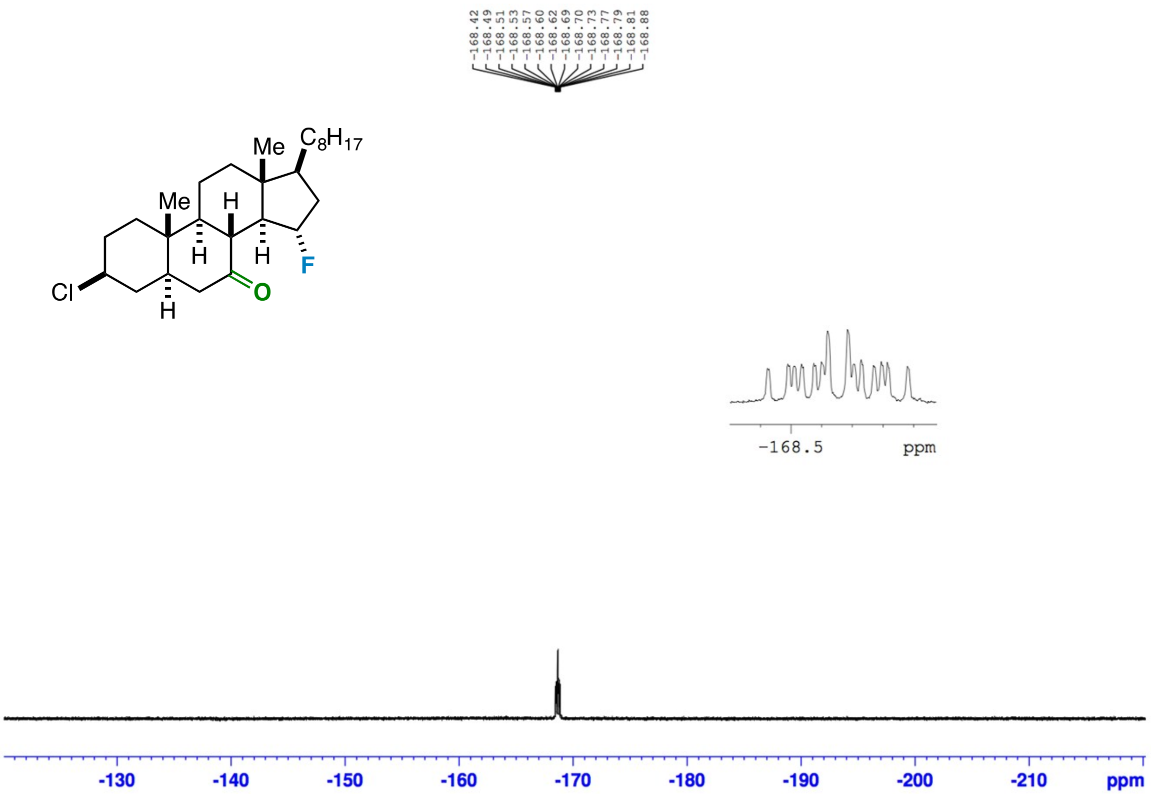

Fig. S22. ^{19}F NMR spectrum ($CDCl_3$, 282 MHz) of compound **10** (major diastereomer).

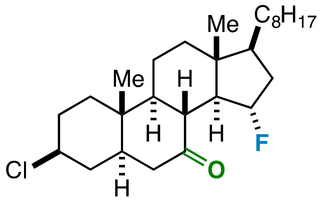

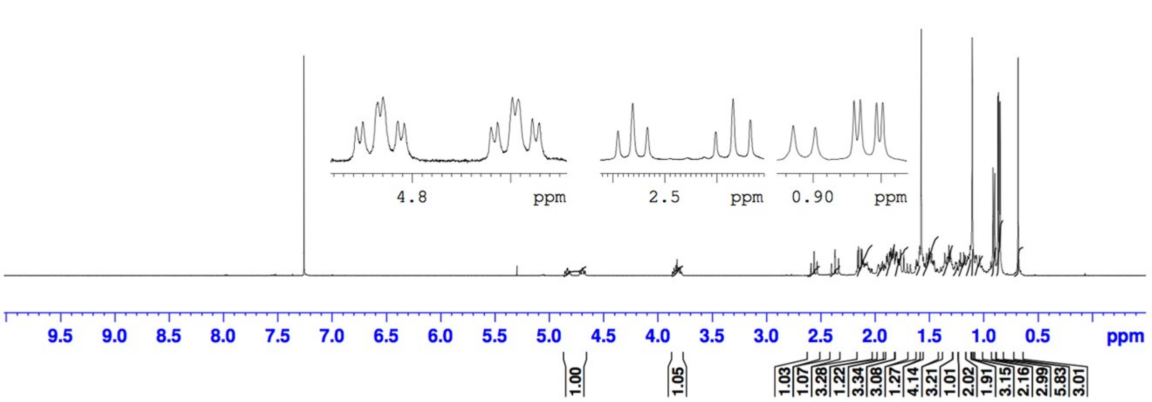

Fig. S23. ^{1}H NMR spectrum ($CDCl_3$, 400 MHz) of compound **10** (major diastereomer).

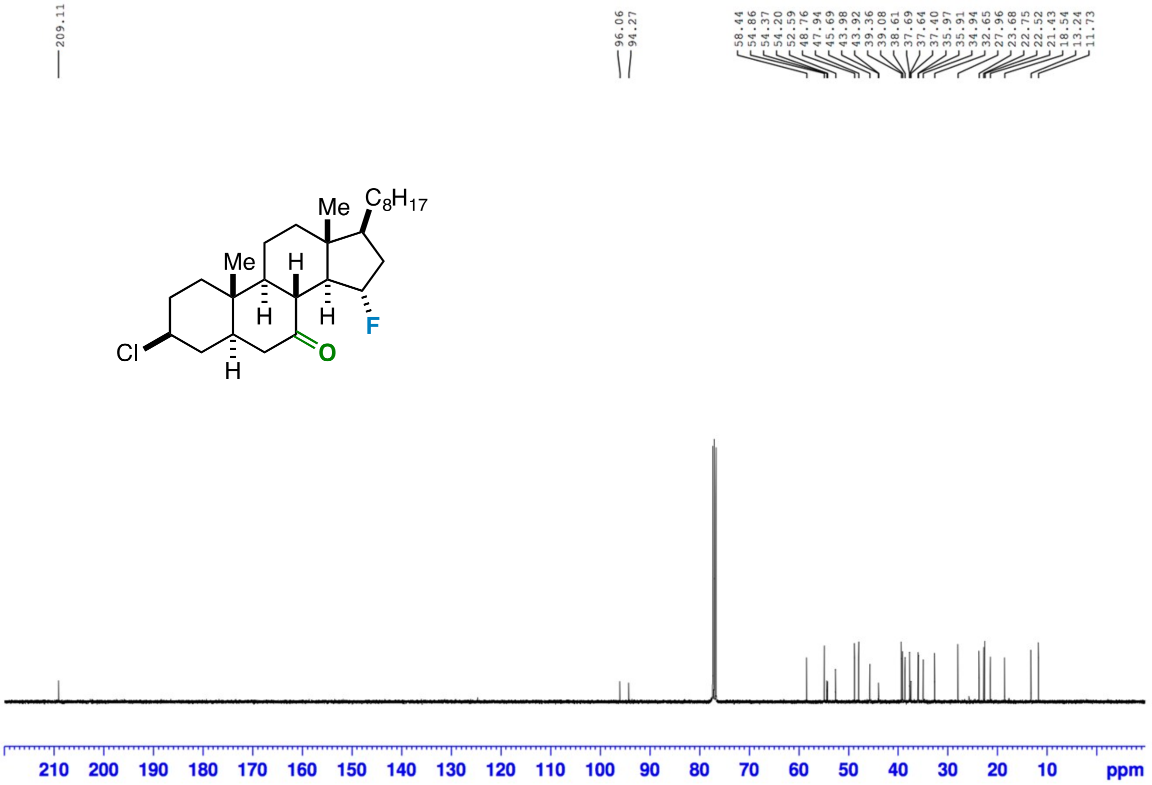

Fig. S24. ^{13}C NMR spectrum ($CDCl_3$, 100 MHz) of compound **10** (major diastereomer).

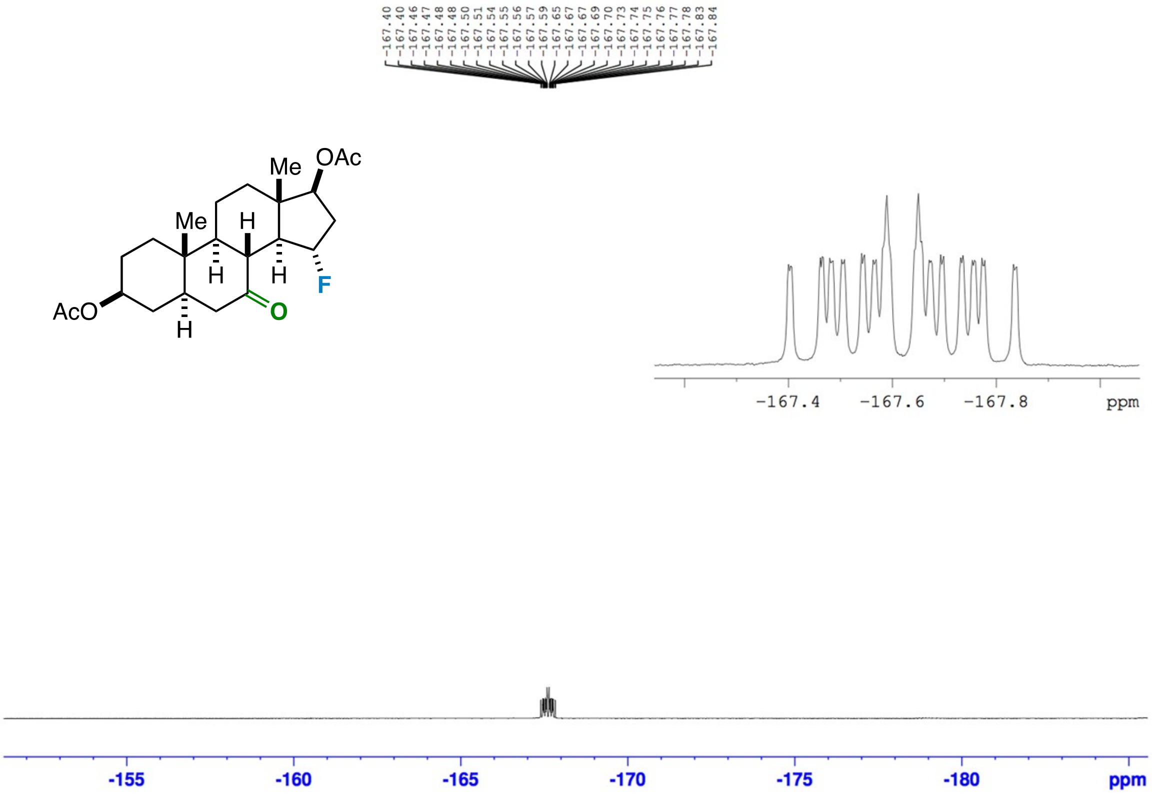

Fig. S25. ^{19}F NMR spectrum ($CDCl_3$, 282 MHz) of compound **11** (major diastereomer).

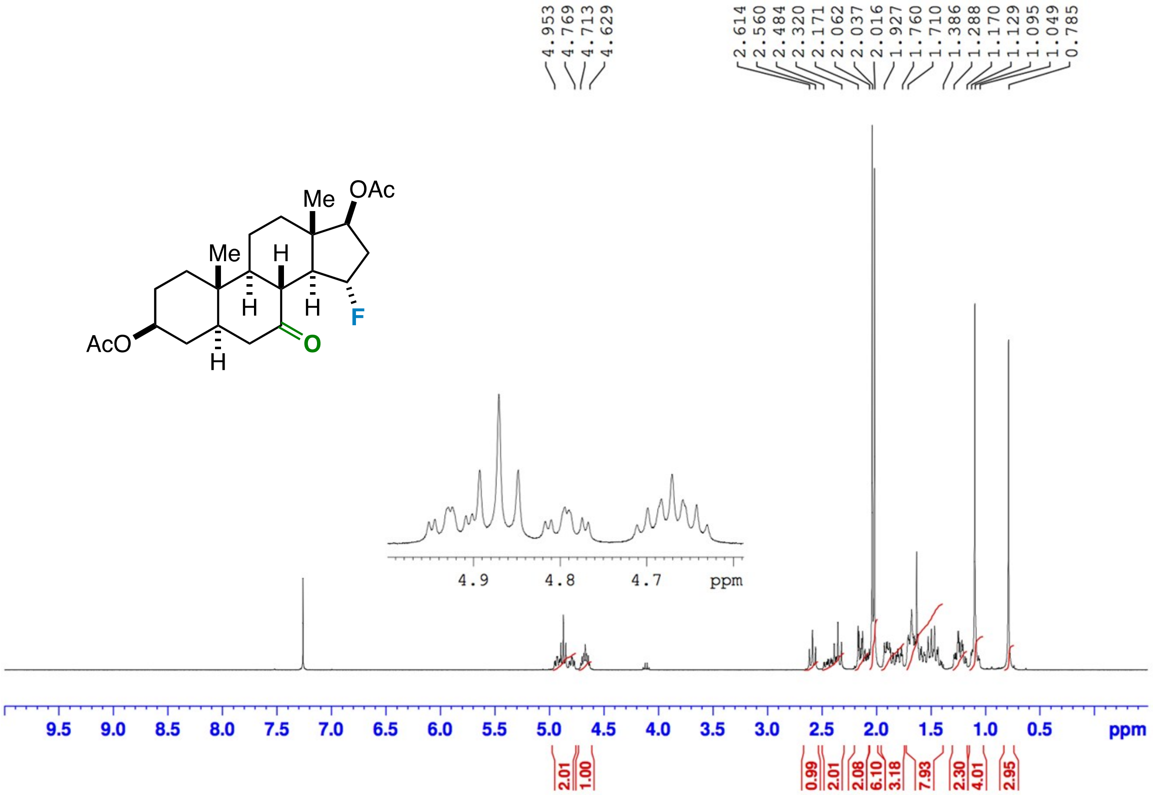

Fig. S26. ^{1}H NMR spectrum ($CDCl_3$, 400 MHz) of compound **11** (major diastereomer).

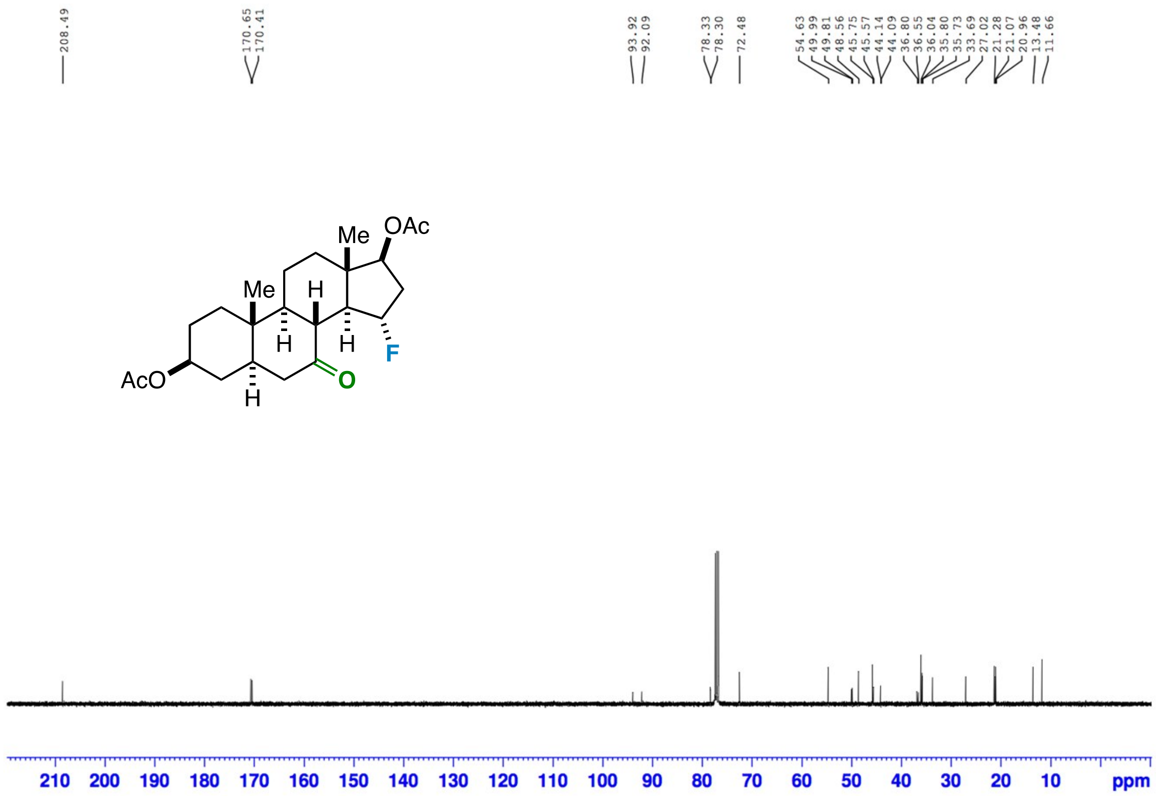

Fig. S27. ^{13}C NMR spectrum ($CDCl_3$, 100 MHz) of compound **11** (major diastereomer).

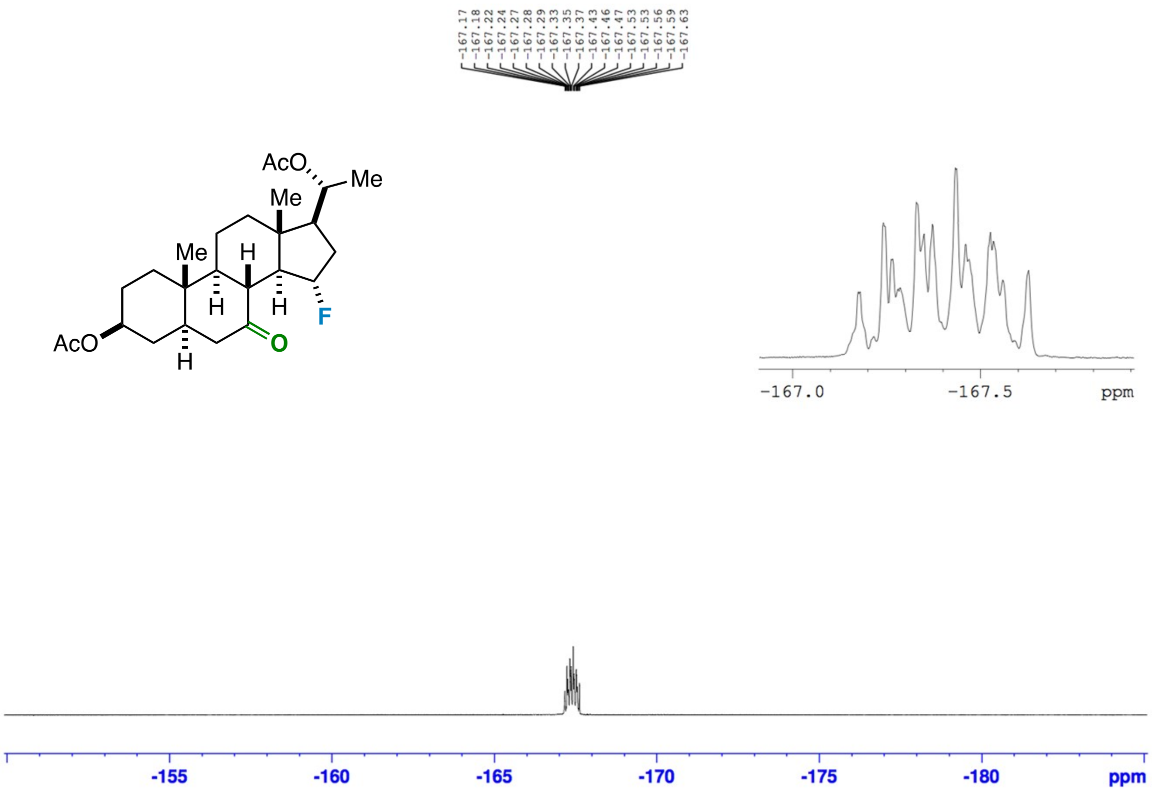

Fig. S28. ^{19}F NMR spectrum ($CDCl_3$, 282 MHz) of compound **12** (major diastereomer).

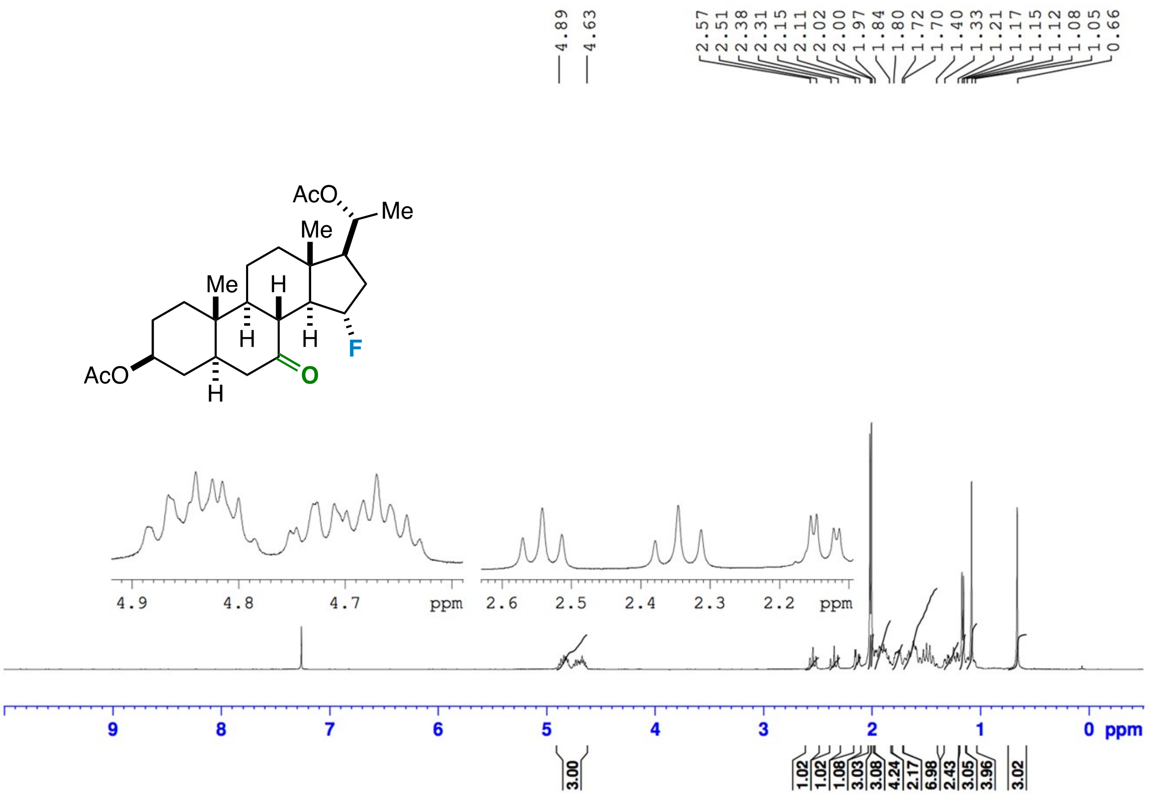

Fig. S29. ^{1}H NMR spectrum ($CDCl_3$, 400 MHz) of compound **12** (major diastereomer).

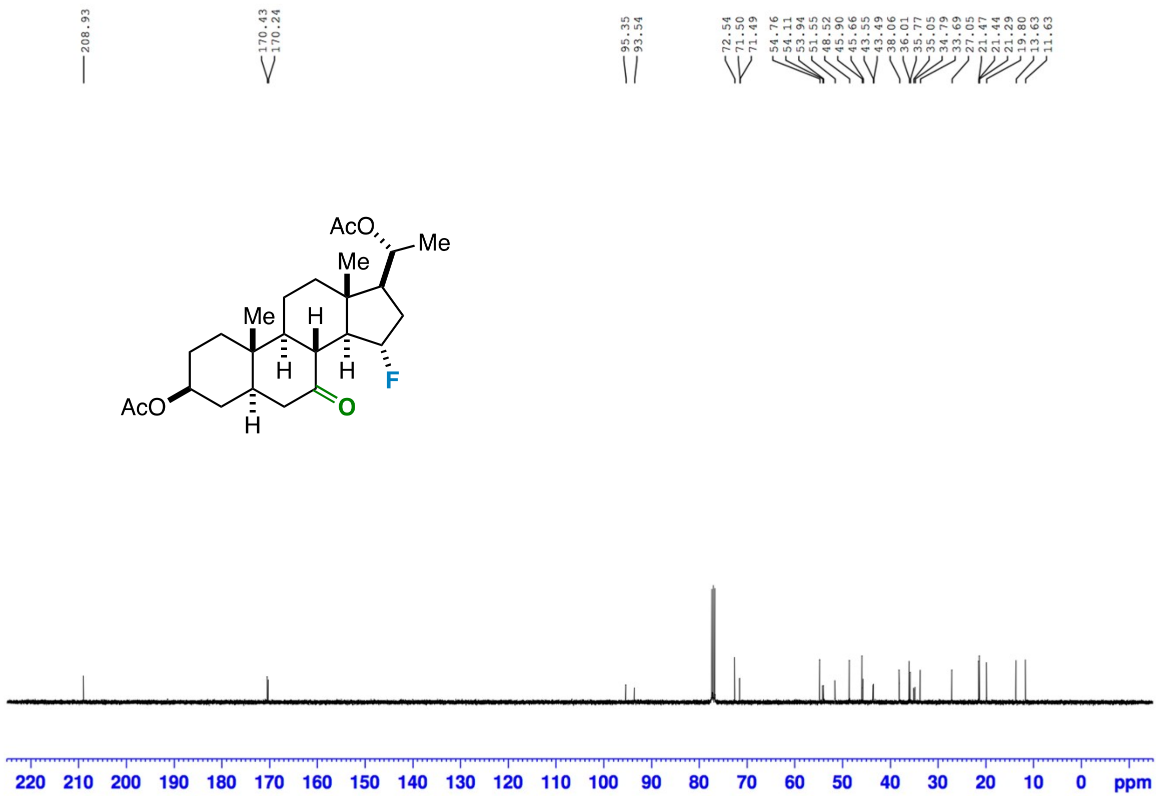

Fig. S30. ^{13}C NMR spectrum ($CDCl_3$, 100 MHz) of compound **12** (major diastereomer).

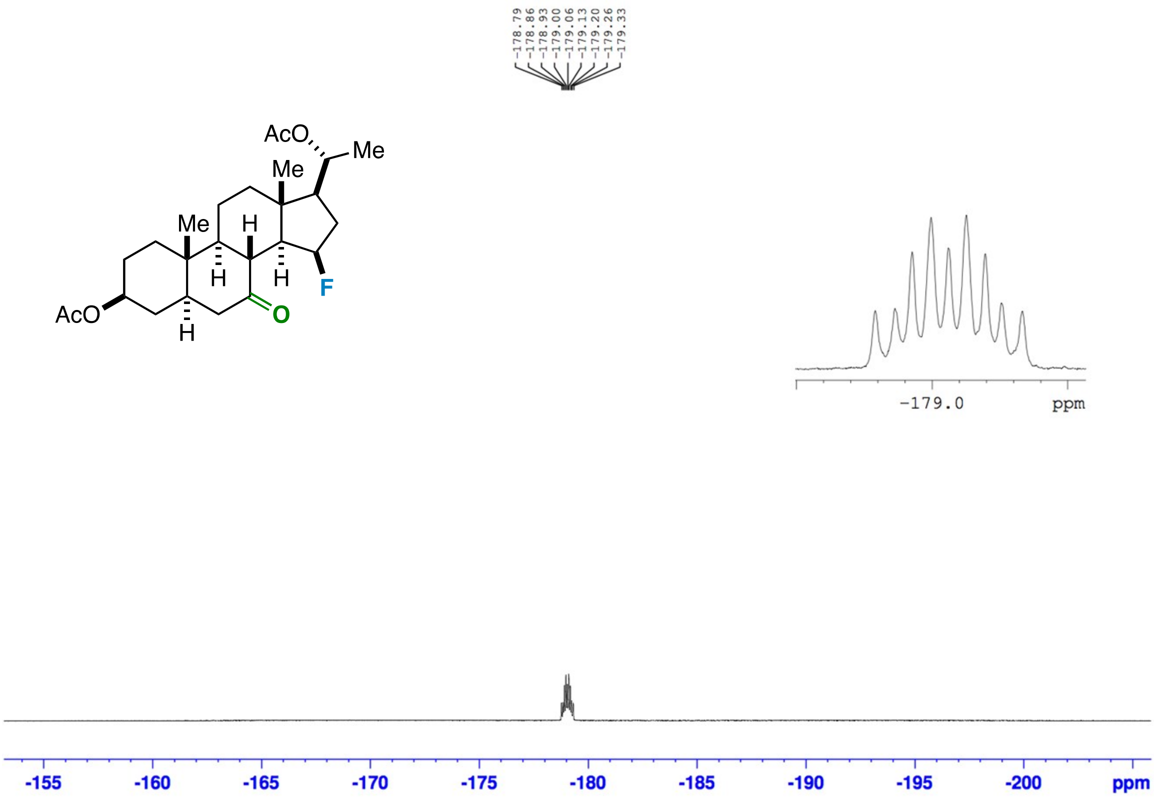

Fig. S31. ^{19}F NMR spectrum ($CDCl_3$, 282 MHz) of compound **12** (minor diastereomer).

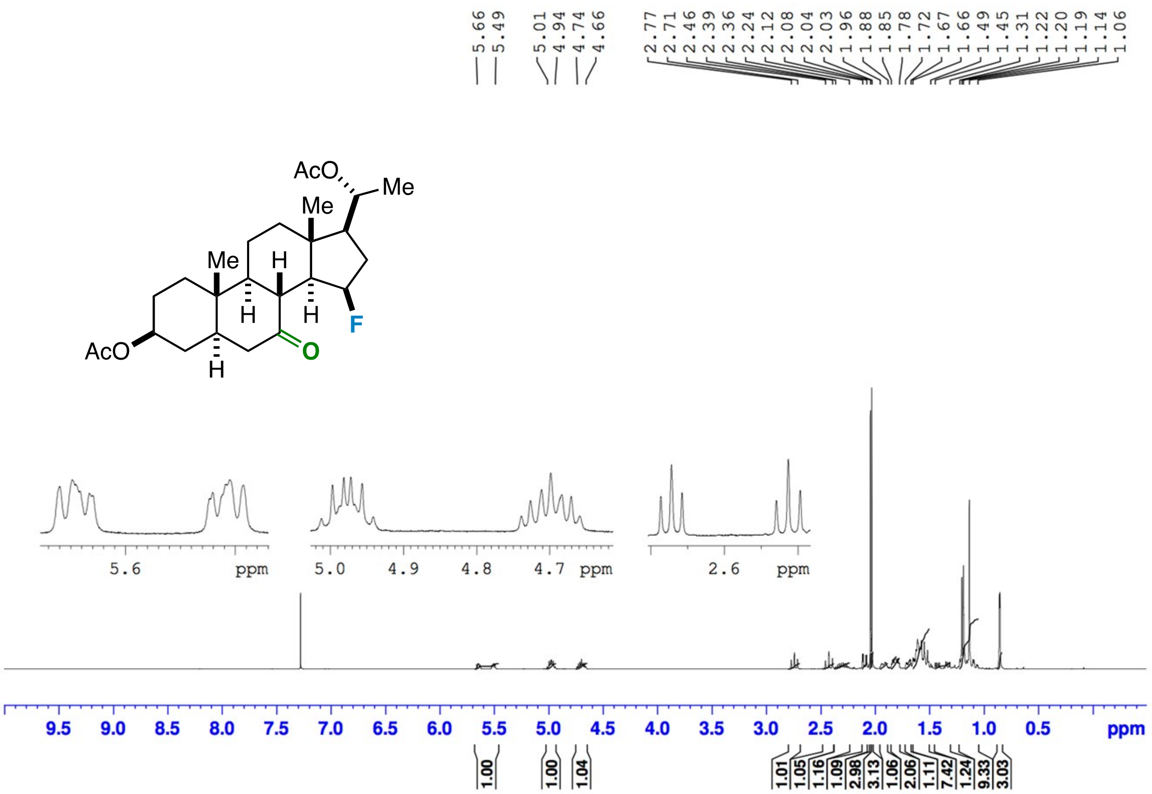

Fig. S32. ^{1}H NMR spectrum ($CDCl_3$, 400 MHz) of compound **12** (minor diastereomer).

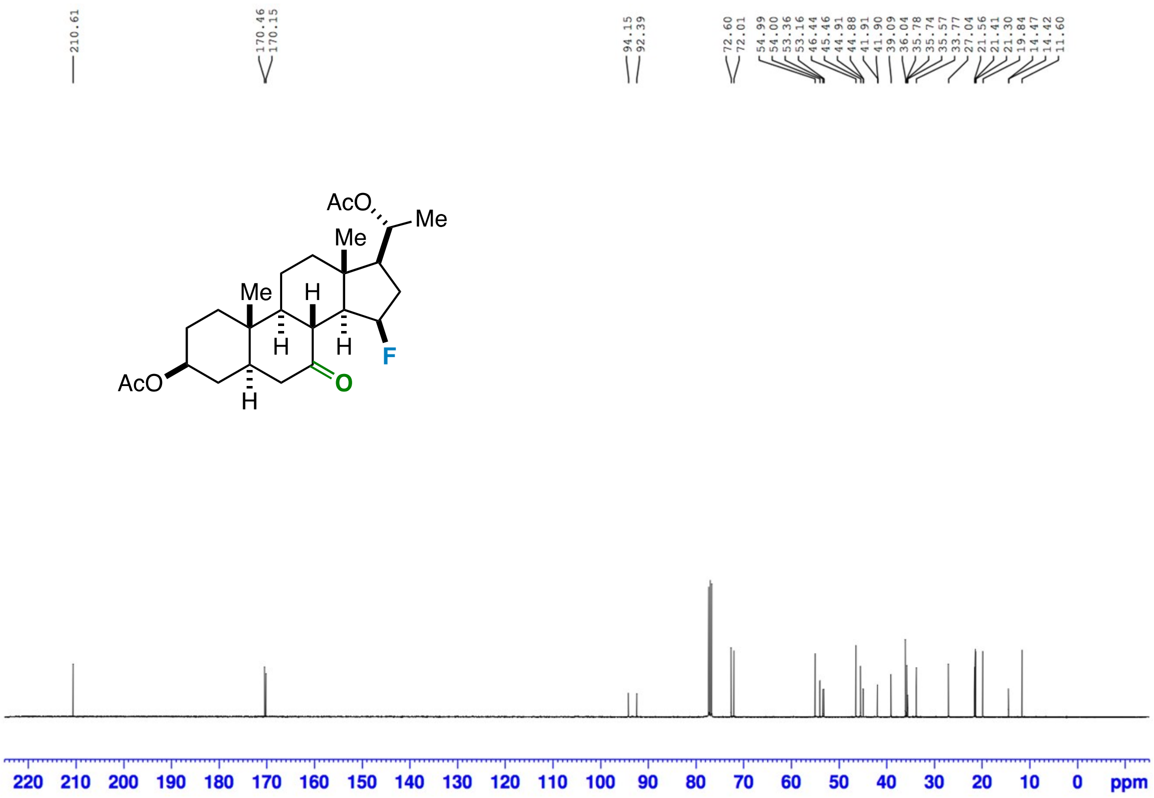

Fig. S33. ^{13}C NMR spectrum ($CDCl_3$, 100 MHz) of compound **12** (minor diastereomer).

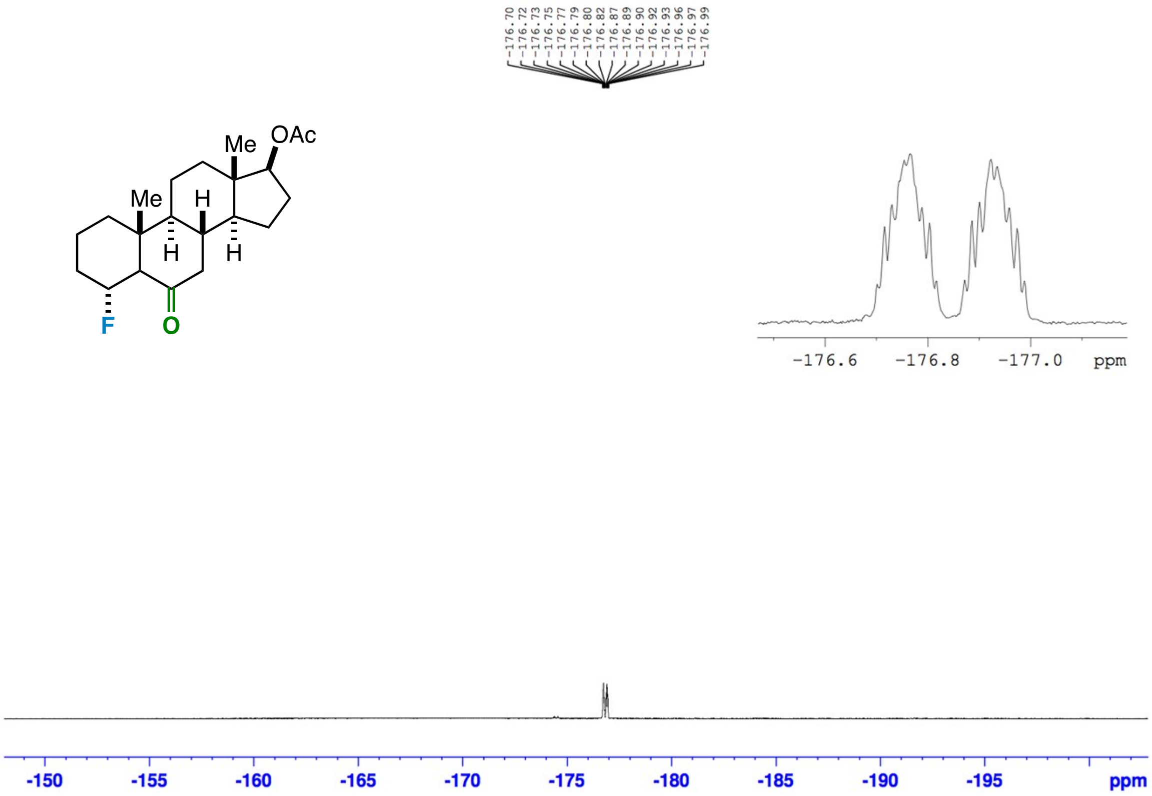

Fig. S34. ^{19}F NMR spectrum ($CDCl_3$, 282 MHz) of compound **13** (major diastereomer).

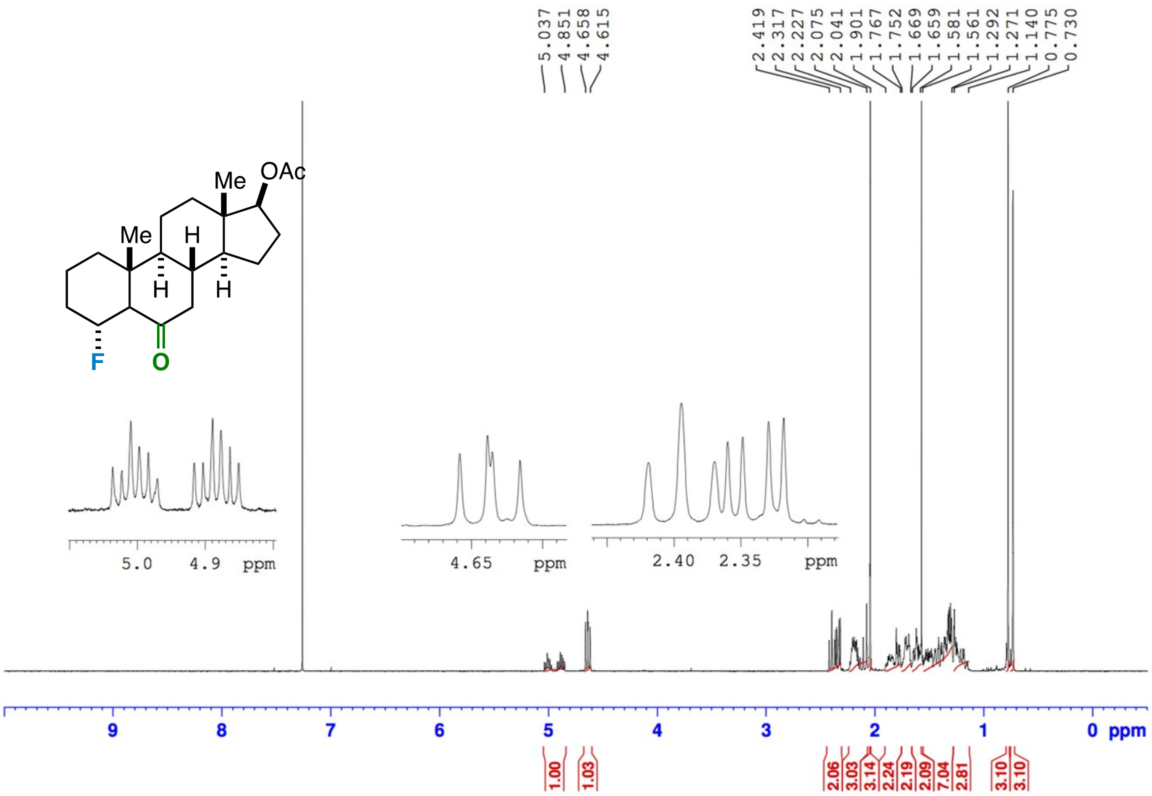

Fig. S35. ^{1}H NMR spectrum ($CDCl_3$, 400 MHz) of compound **13** (major diastereomer).

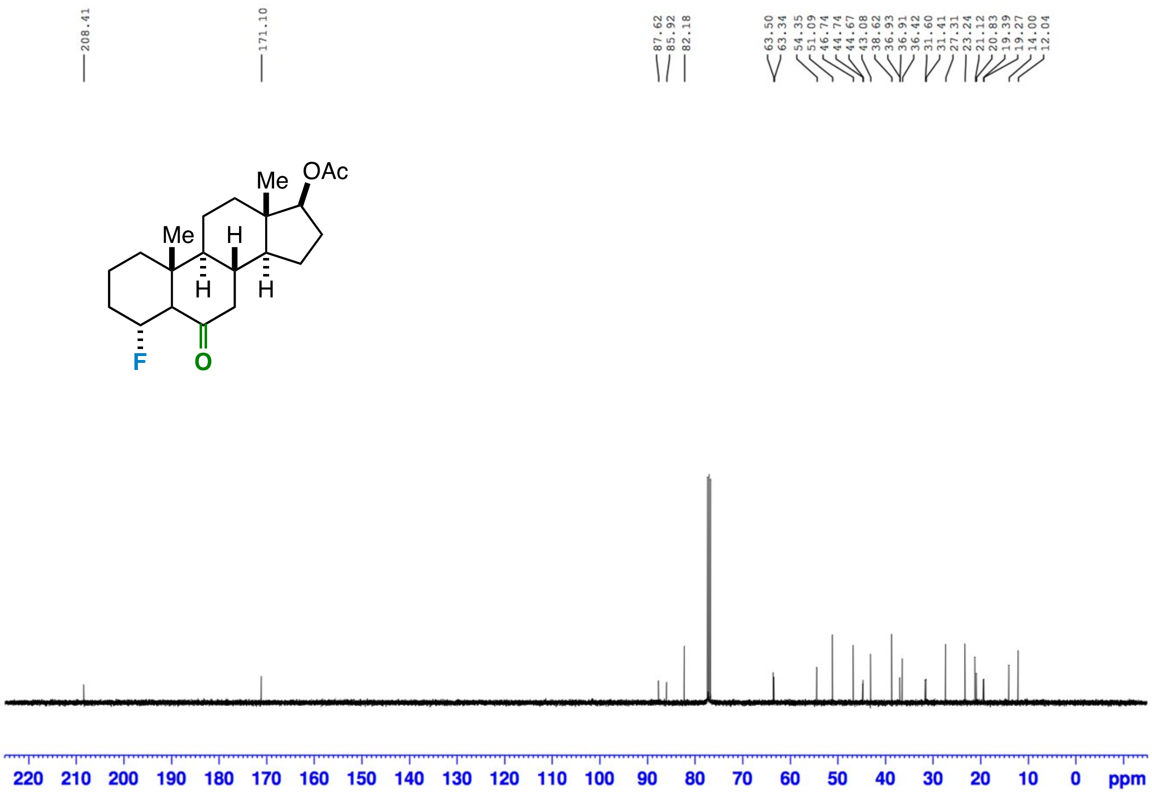

Fig. S36. ^{13}C NMR spectrum ($CDCl_3$, 100 MHz) of compound **13** (major diastereomer).

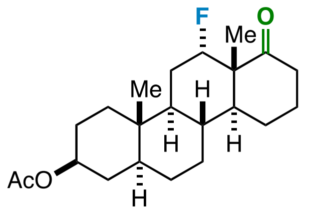

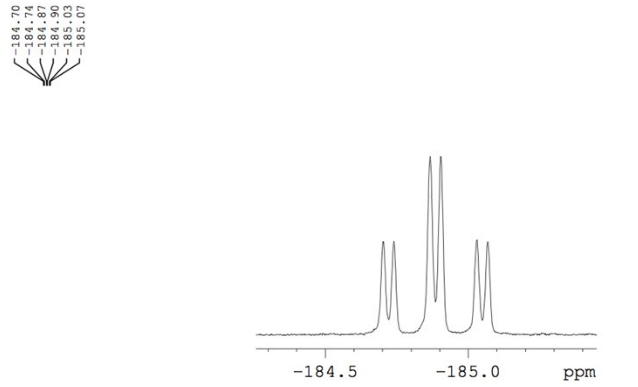

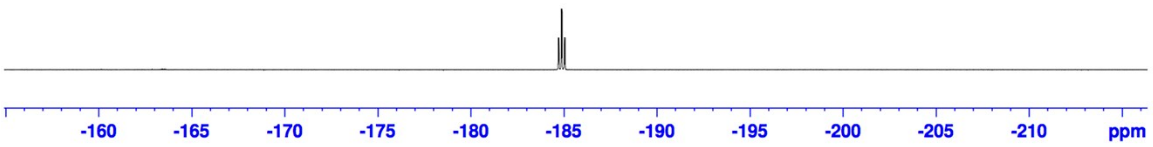

Fig. S37. ^{19}F NMR spectrum ($CDCl_3$, 282 MHz) of compound **14** (major diastereomer).

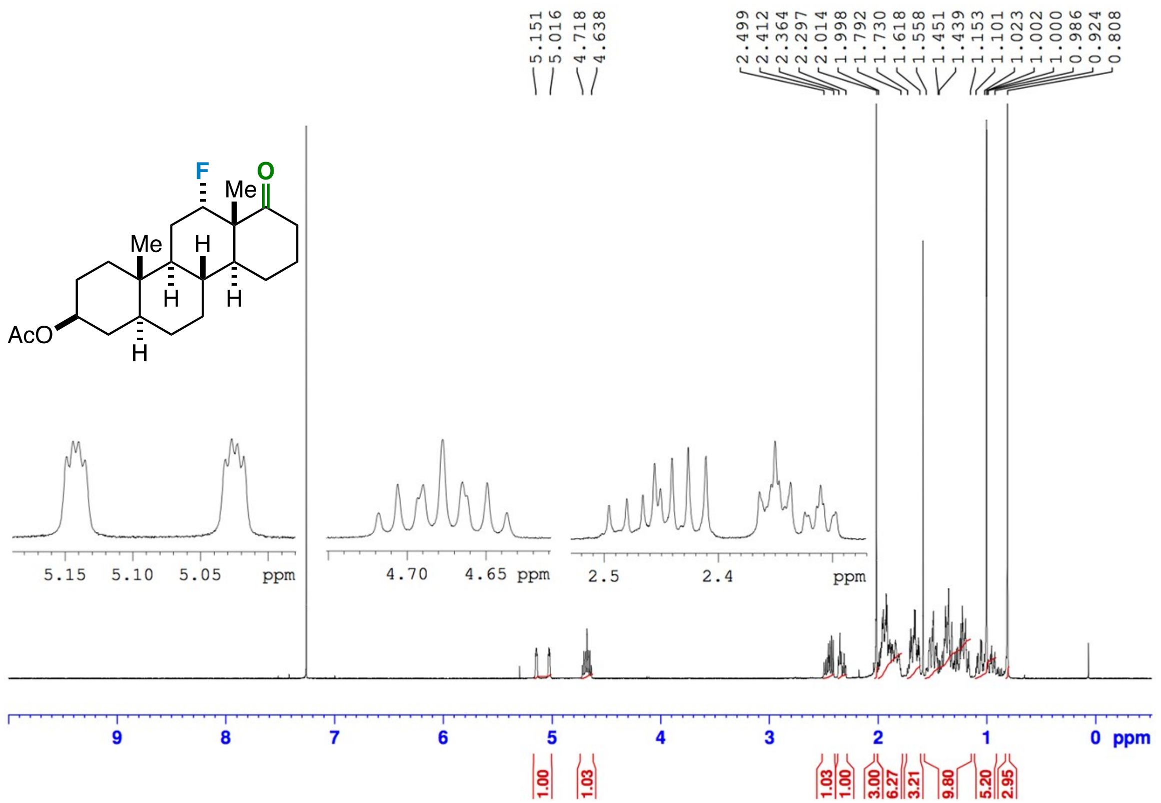

Fig. S38. ^{1}H NMR spectrum ($CDCl_3$, 400 MHz) of compound **14** (major diastereomer).

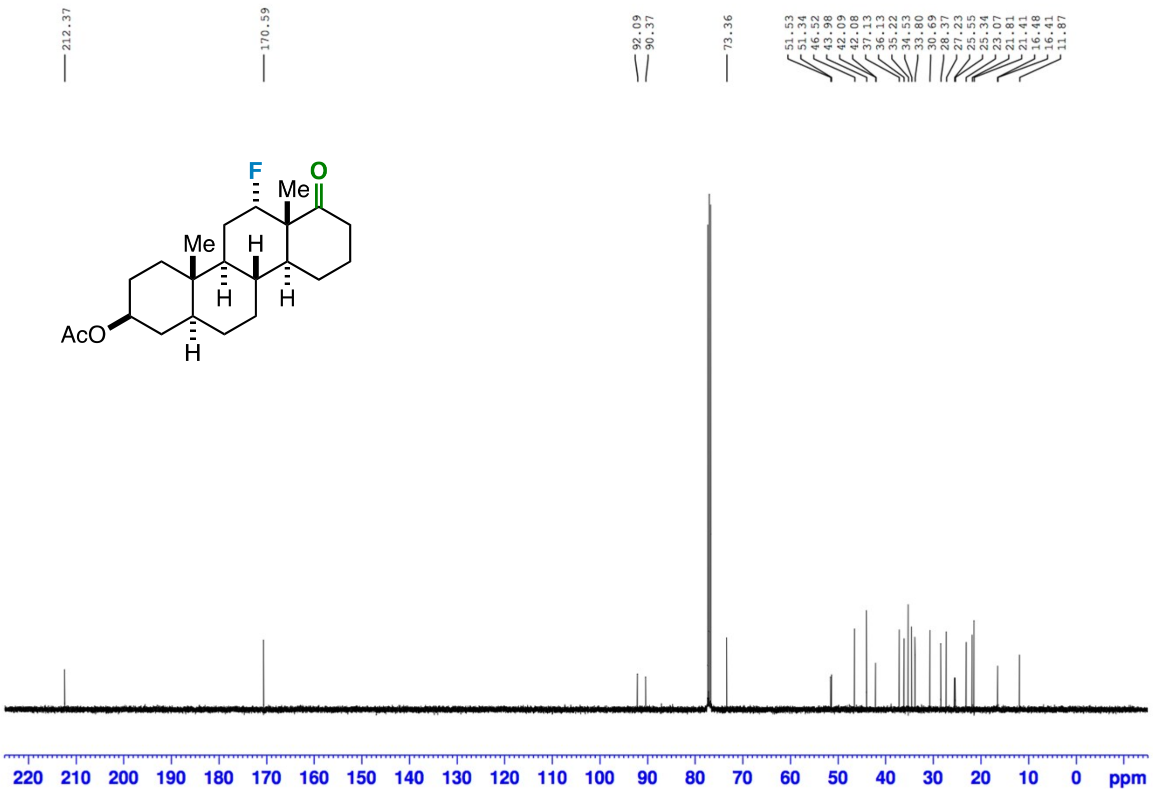

Fig. S39. ^{13}C NMR spectrum ($CDCl_3$, 100 MHz) of compound **14** (major diastereomer).

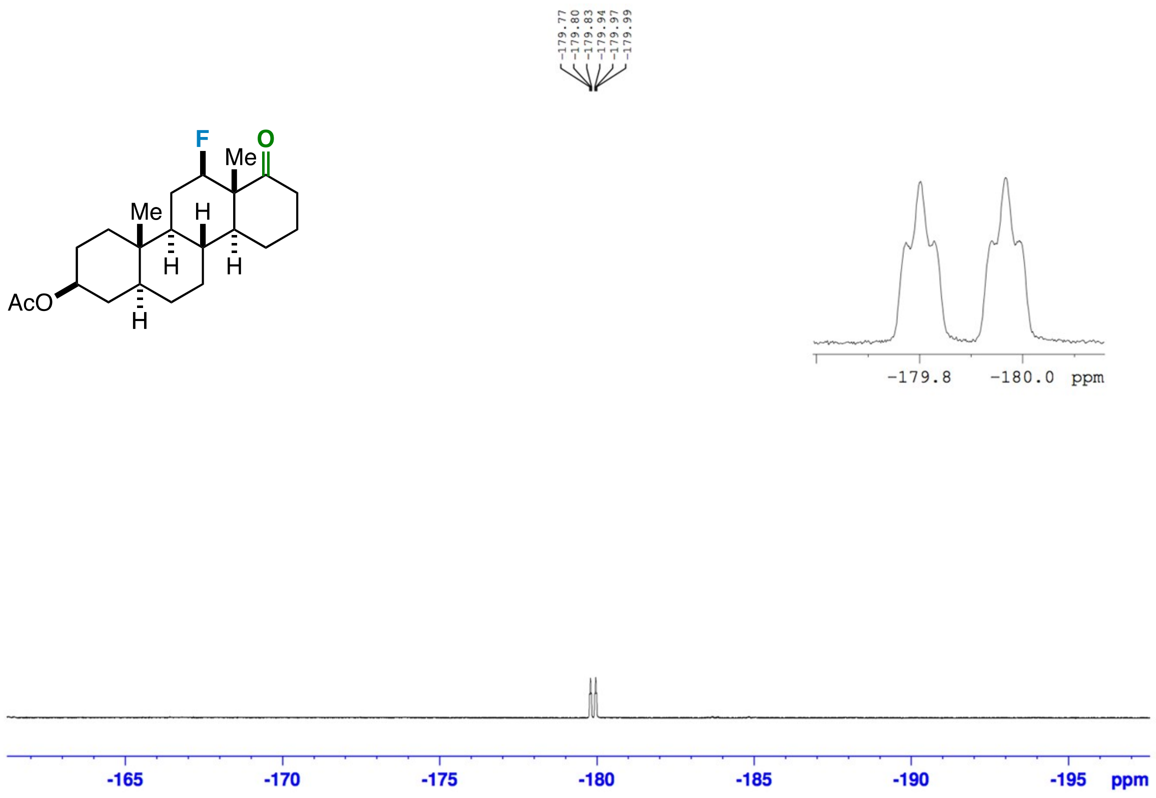

Fig. S40. ^{19}F NMR spectrum ($CDCl_3$, 282 MHz) of compound **14** (minor diastereomer).

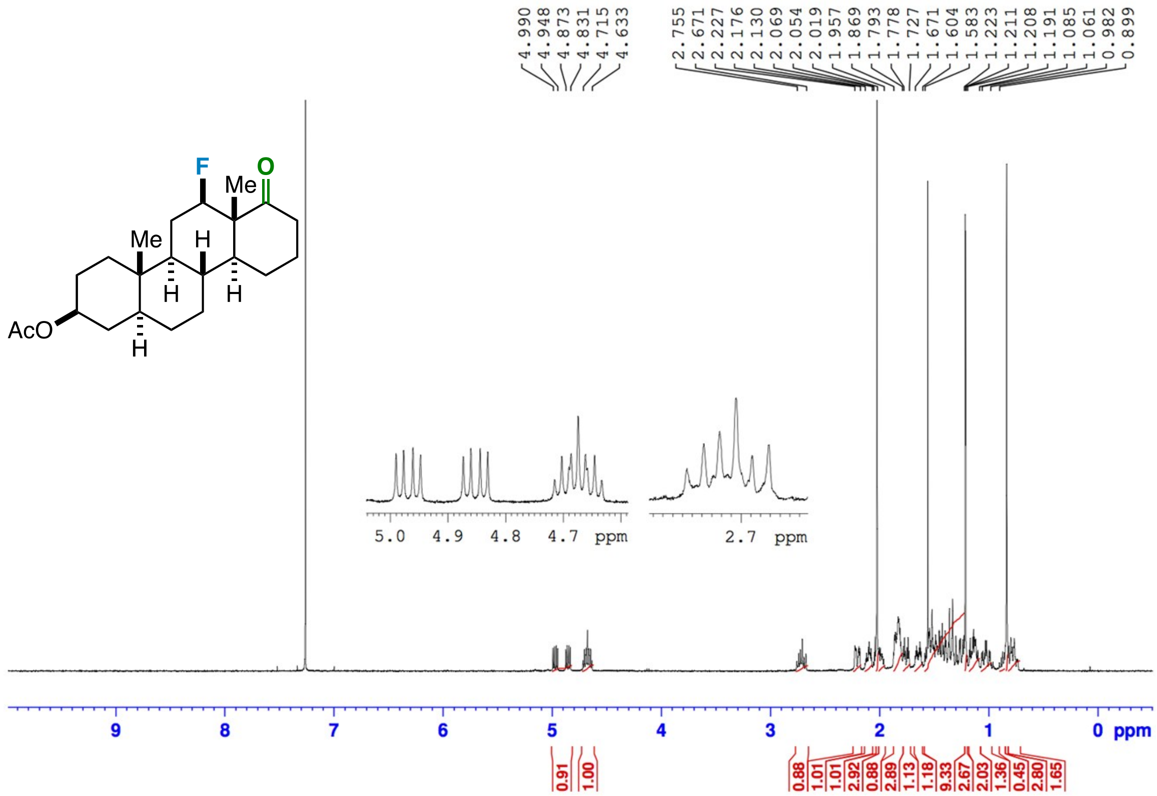

Fig. S41. ^{1}H NMR spectrum ($CDCl_3$, 400 MHz) of compound **14** (minor diastereomer).

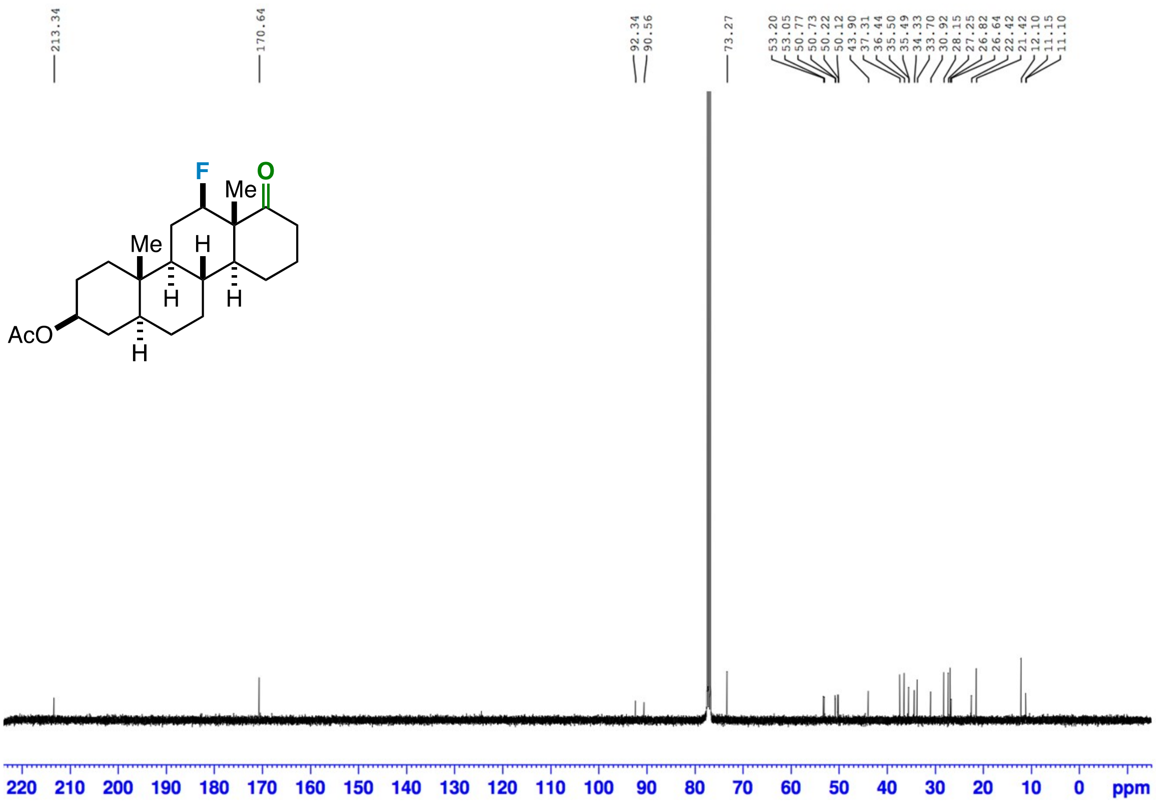

Fig. S42. ^{13}C NMR spectrum ($CDCl_3$, 100 MHz) of compound **14** (minor diastereomer).

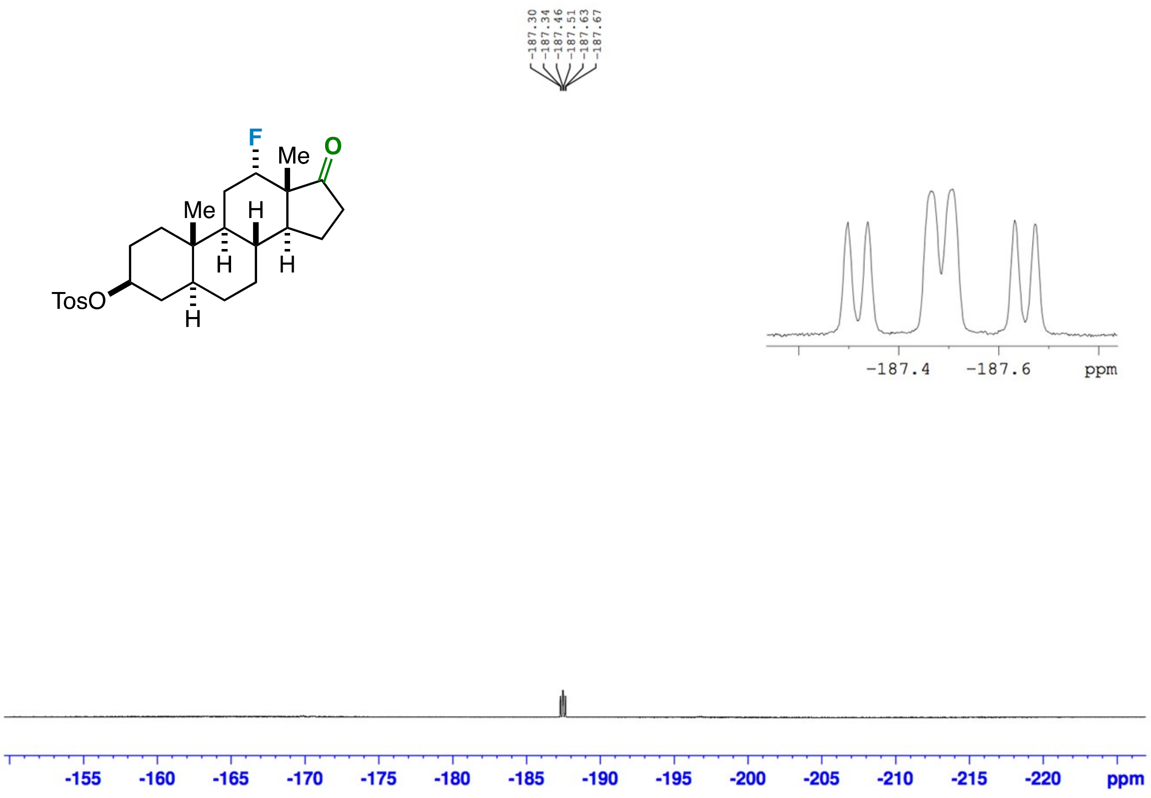

Fig. S43. ^{19}F NMR spectrum ($CDCl_3$, 282 MHz) of compound **15** (major diastereomer).

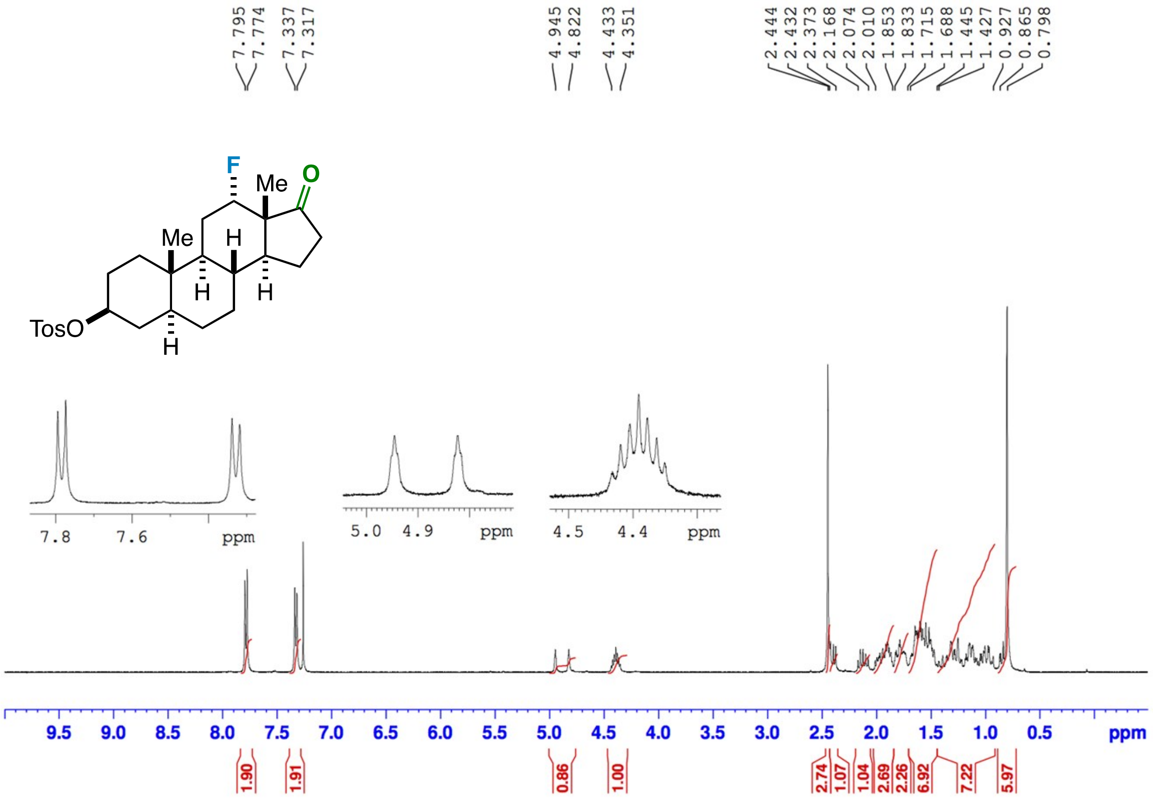

Fig. S44. ^{1}H NMR spectrum ($CDCl_3$, 400 MHz) of compound **15** (major diastereomer).

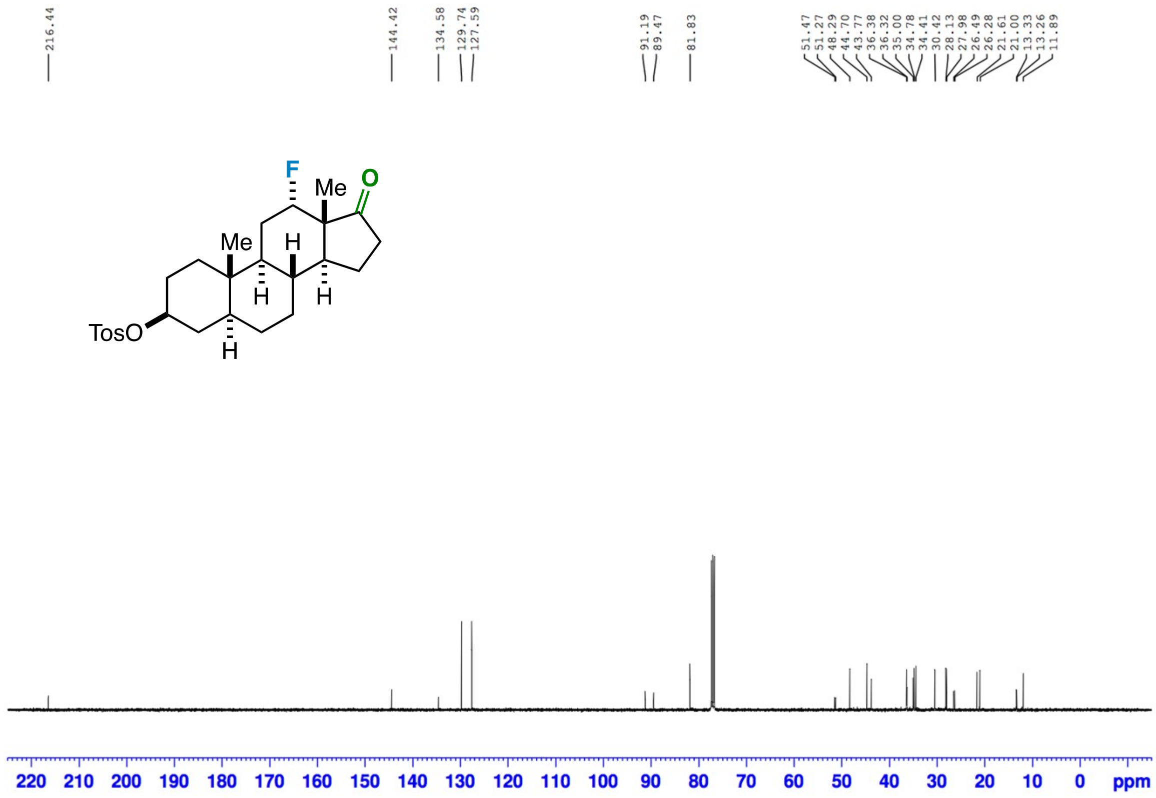

Fig. S45. ^{13}C NMR spectrum ($CDCl_3$, 100 MHz) of compound **15** (major diastereomer).

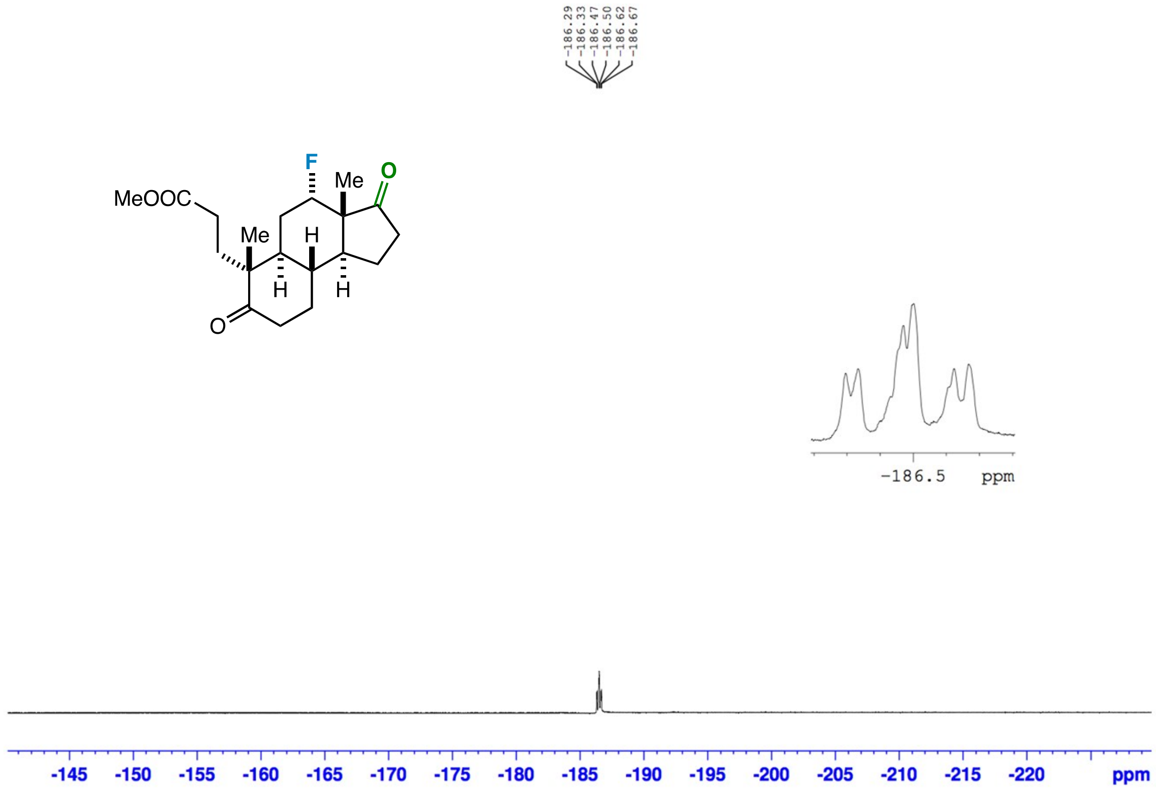

Fig. S46. ^{19}F NMR spectrum ($CDCl_3$, 282 MHz) of compound **16** (major diastereomer).

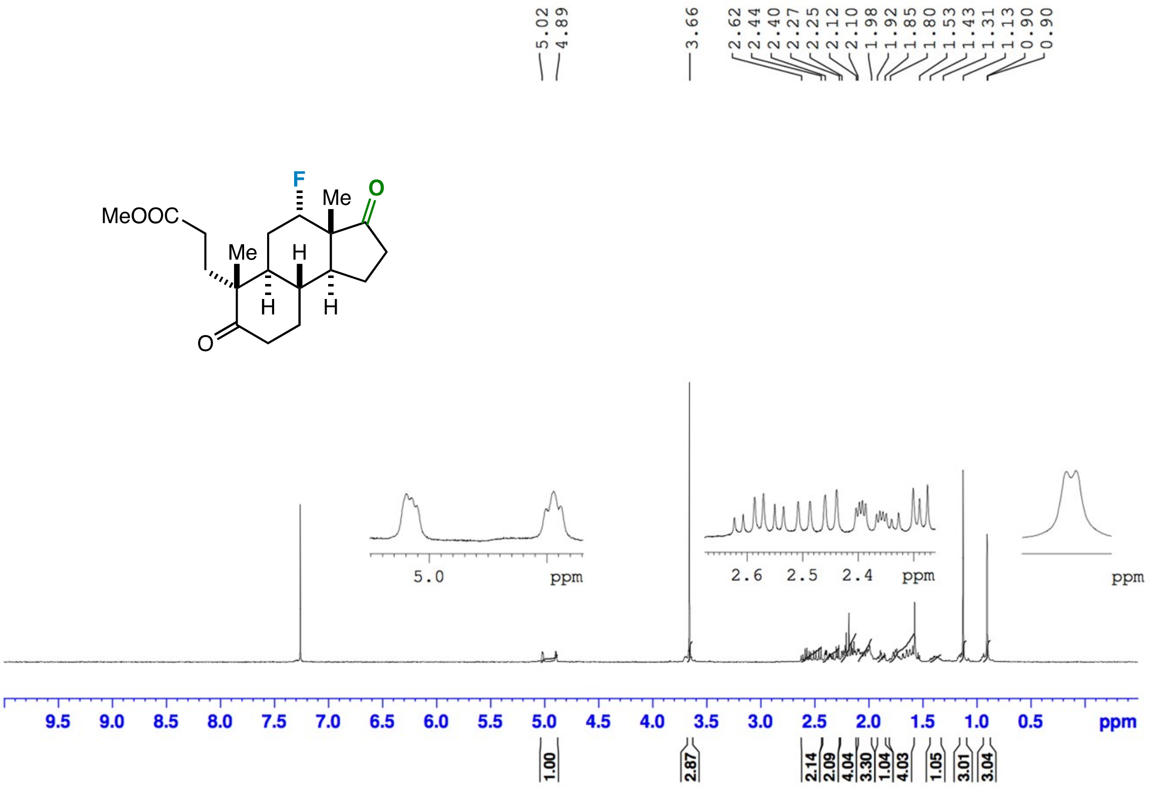

Fig. S47. ^{1}H NMR spectrum ($CDCl_3$, 400 MHz) of compound **16** (major diastereomer).

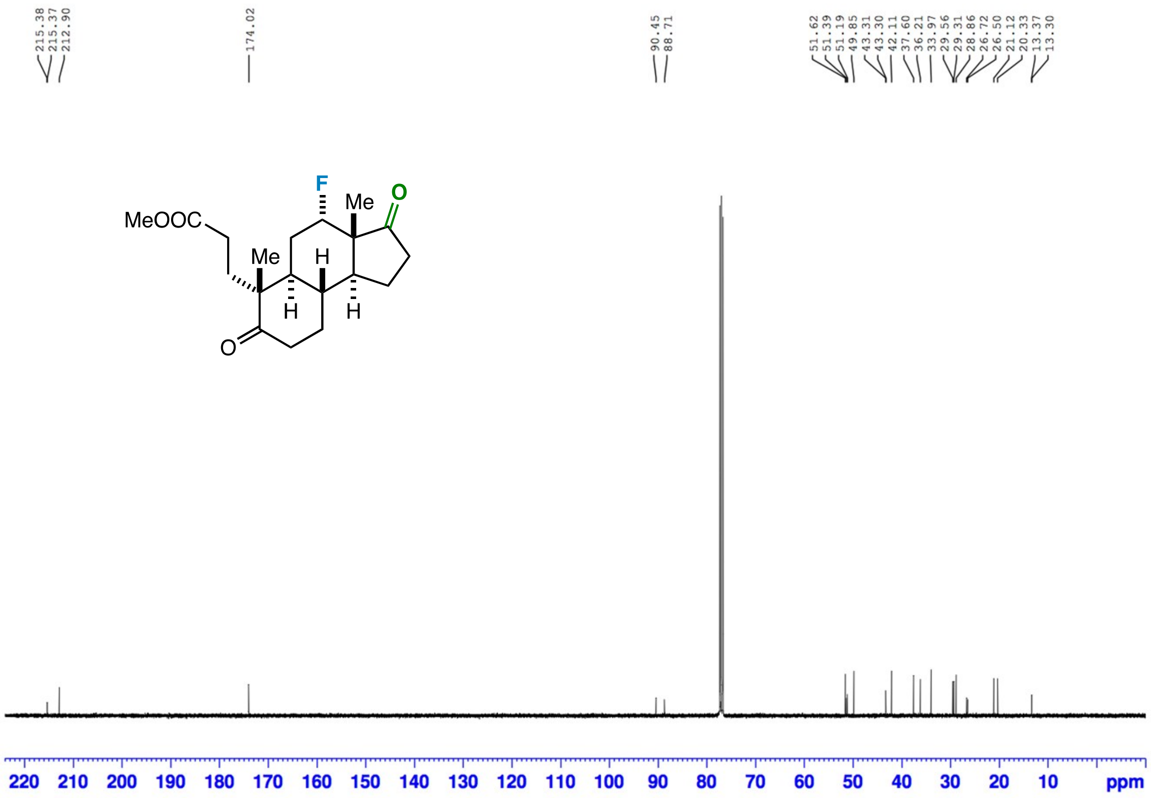

Fig. S48. ^{13}C NMR spectrum ($CDCl_3$, 100 MHz) of compound **16** (major diastereomer).

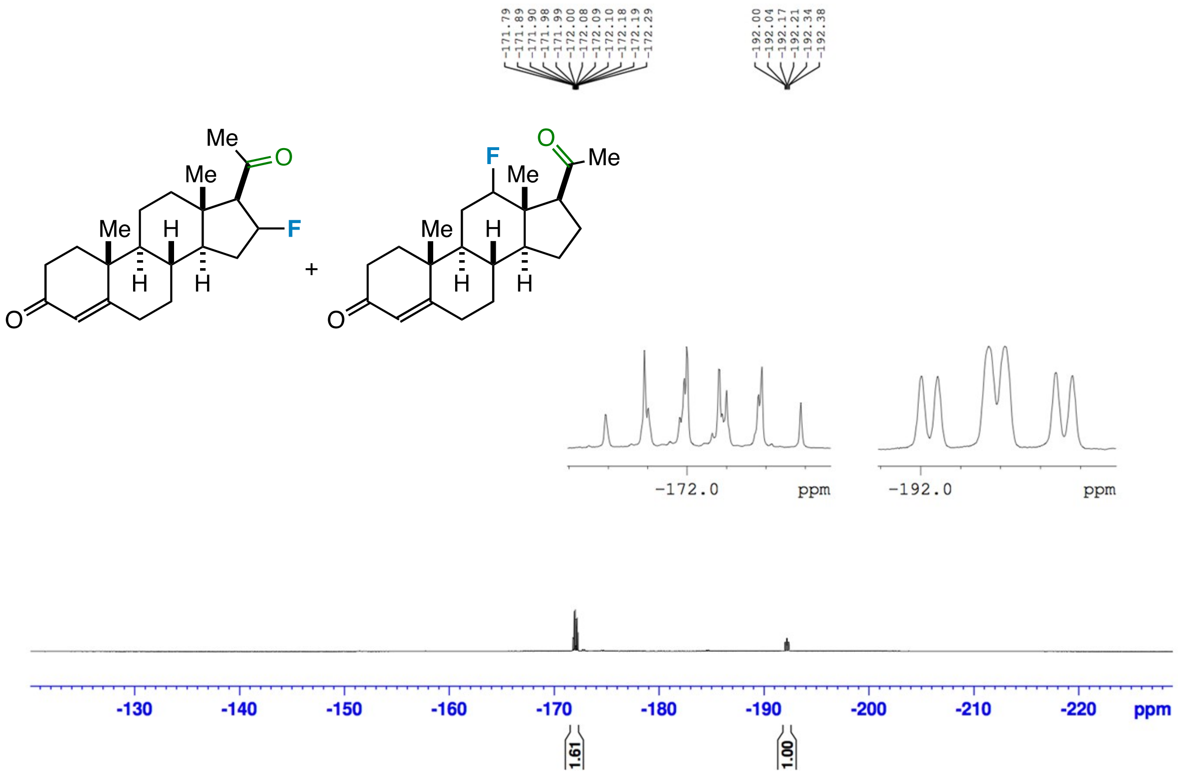

Fig. S49. ^{19}F NMR spectrum ($CDCl_3$, 282 MHz) of compound **17** and **18** (mixture of regioisomers).

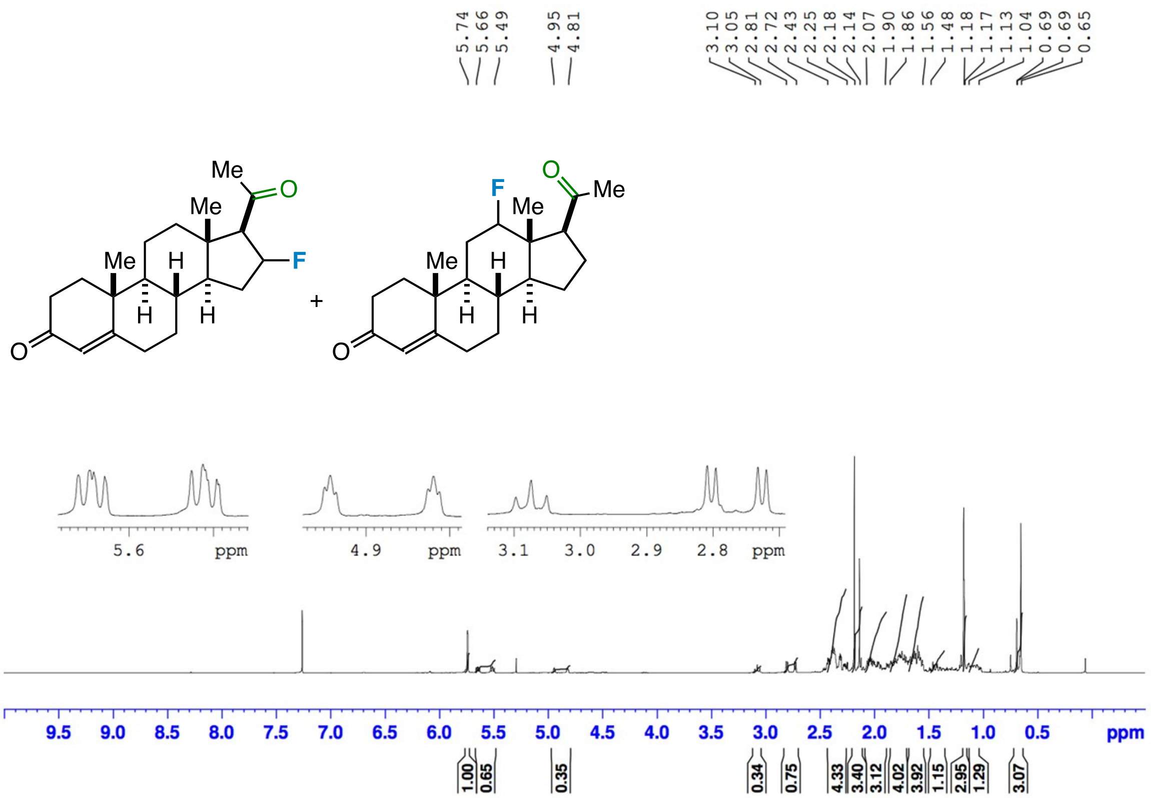

Fig. S50. ^{1}H NMR spectrum ($CDCl_3$, 400 MHz) of compound **17** and **18** (mixture of regioisomers).

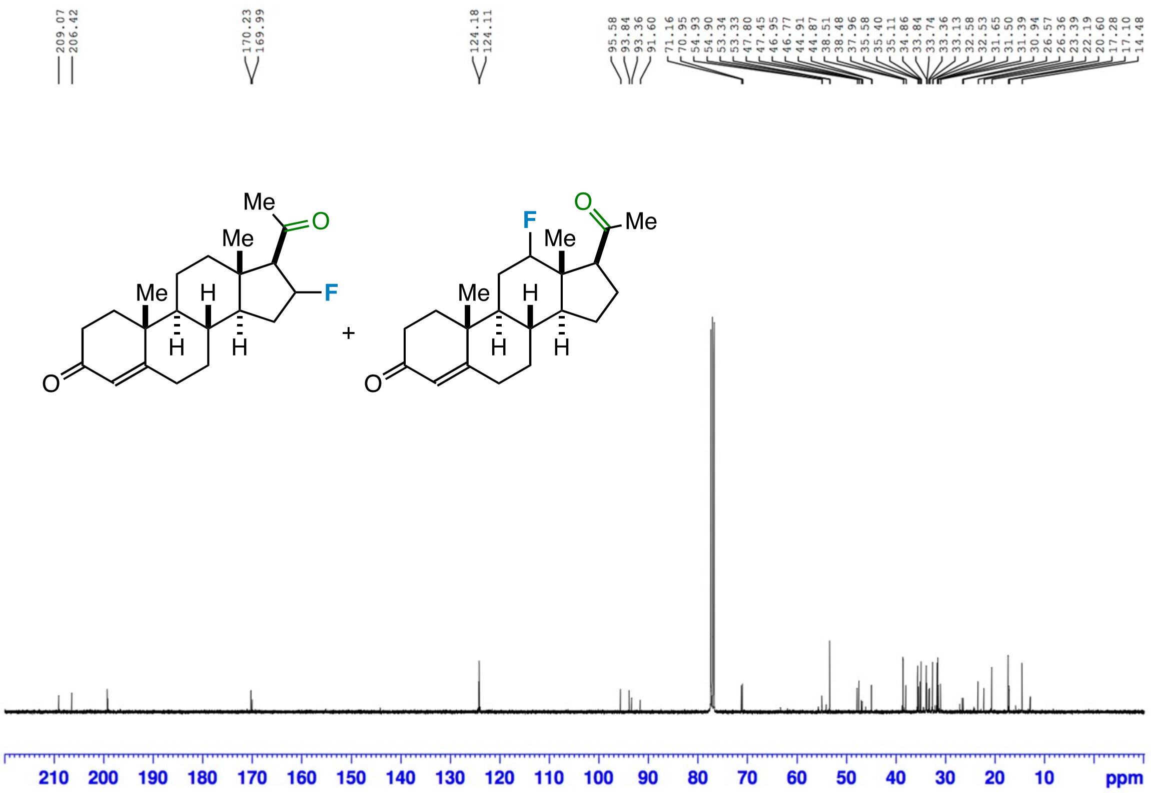

Fig. S51. ^{13}C NMR spectrum ($CDCl_3$, 100 MHz) of compound **17** and **18** (mixture of regioisomers).

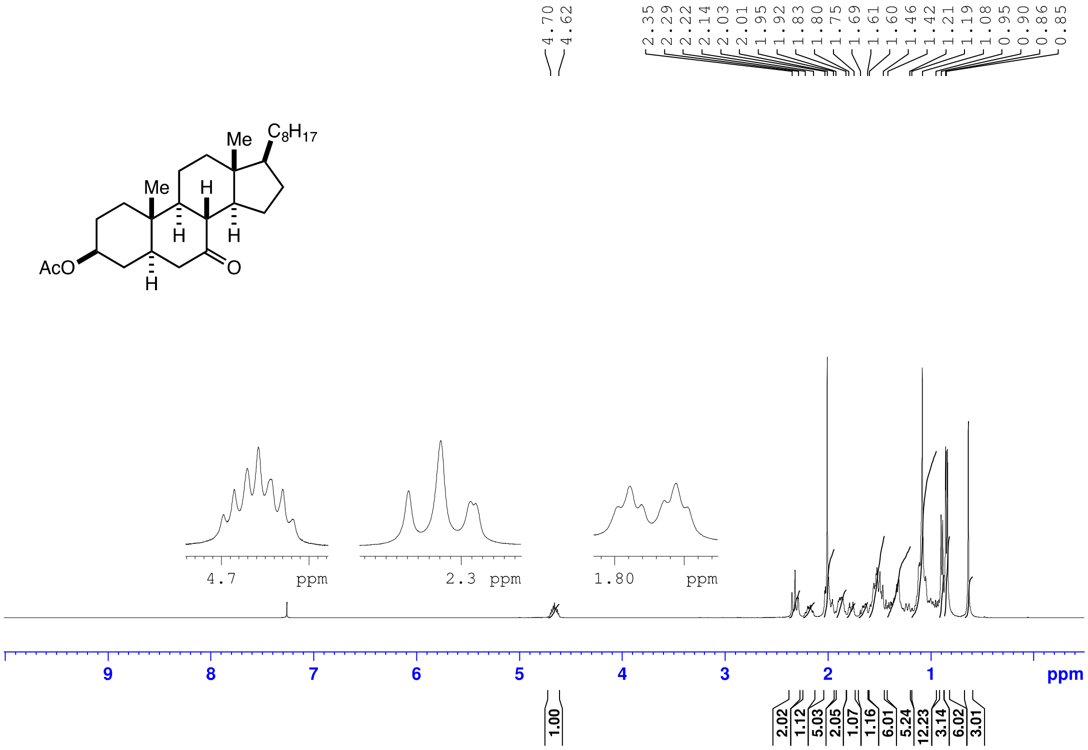

Fig. S52. ^{1}H NMR spectrum ($CDCl_3$, 400 MHz) of compound **1**.

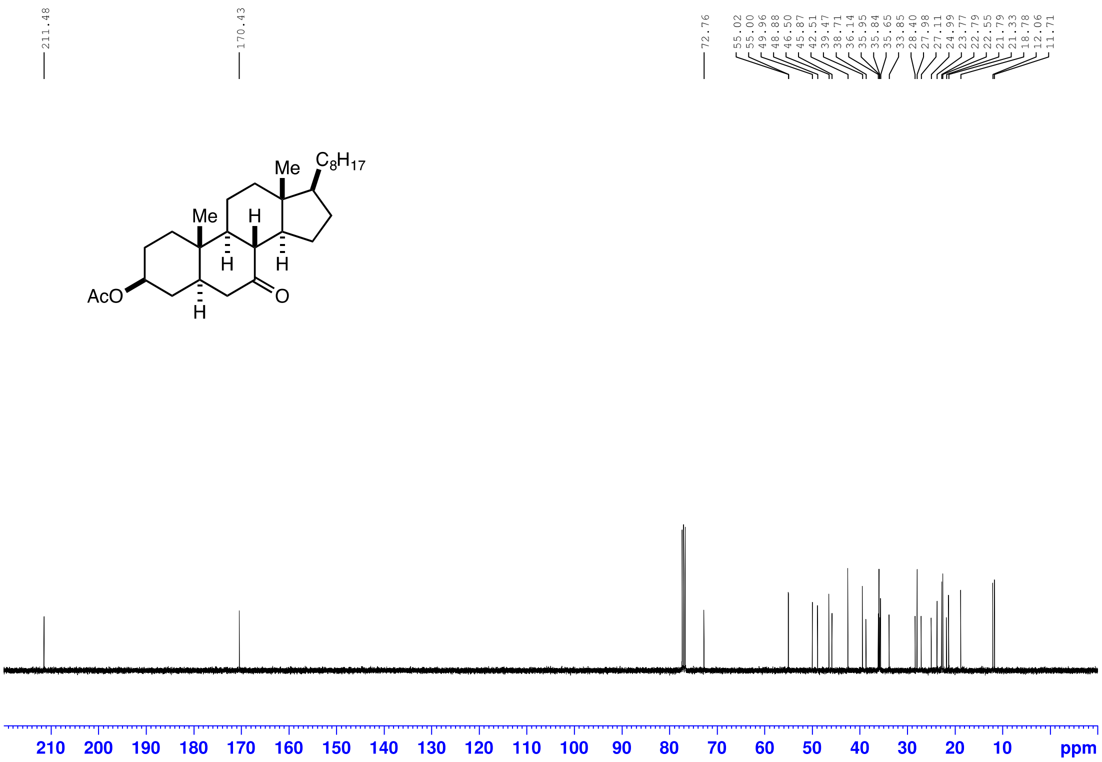

Fig. S53. ^{13}C NMR spectrum ($CDCl_3$, 100 MHz) of compound **1**.

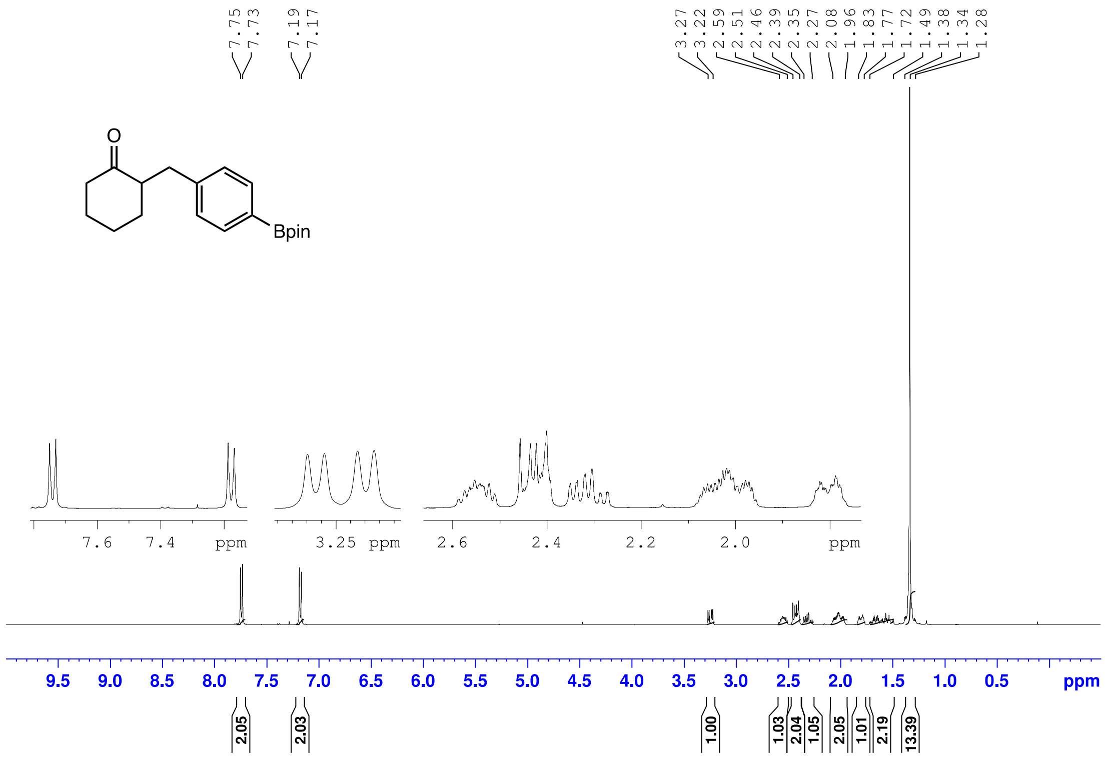

Fig. S54. ^{1}H NMR spectrum ($CDCl_3$, 400 MHz) of starting material for compound **4**.

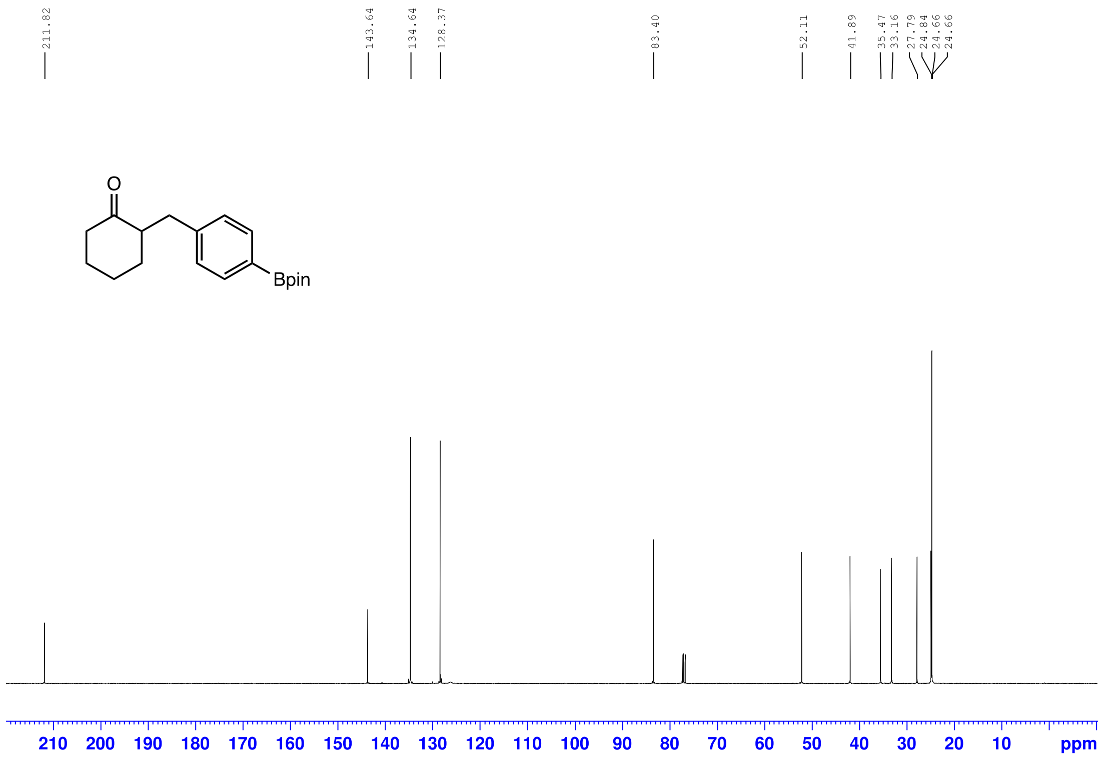

Fig. S55. ^{13}C NMR spectrum ($CDCl_3$, 100 MHz) of starting material for compound **4**.

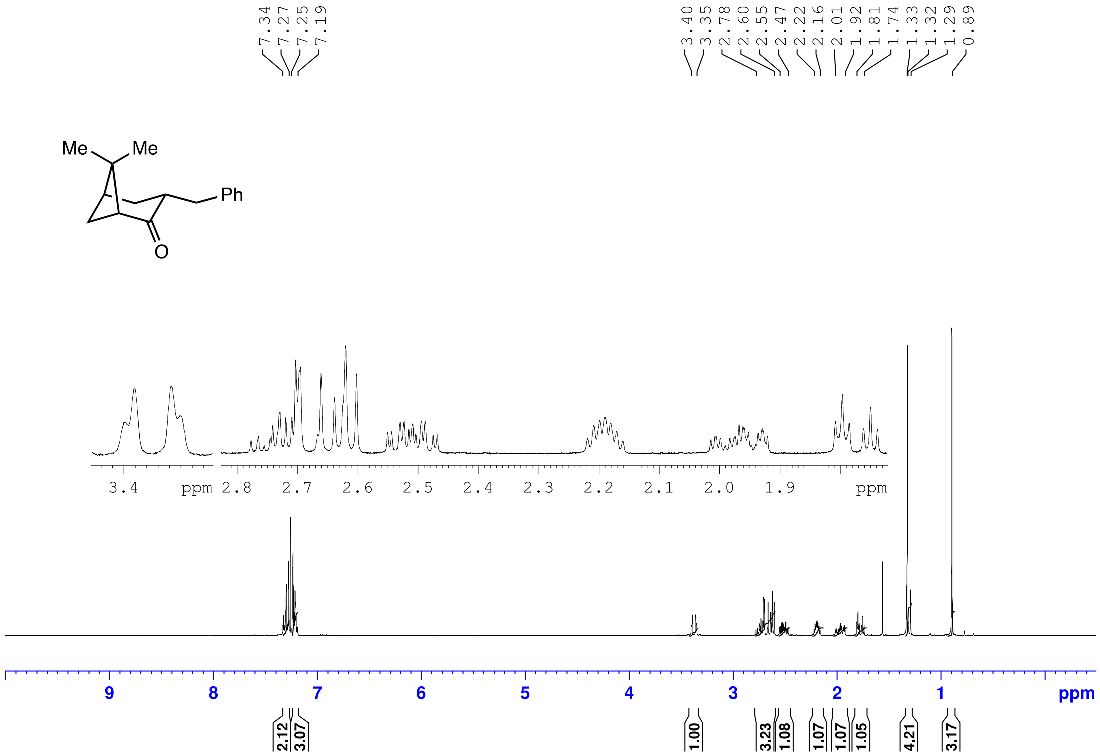

Fig. S56. ^{1}H NMR spectrum ($CDCl_3$, 400 MHz) of starting material for compound **5**.

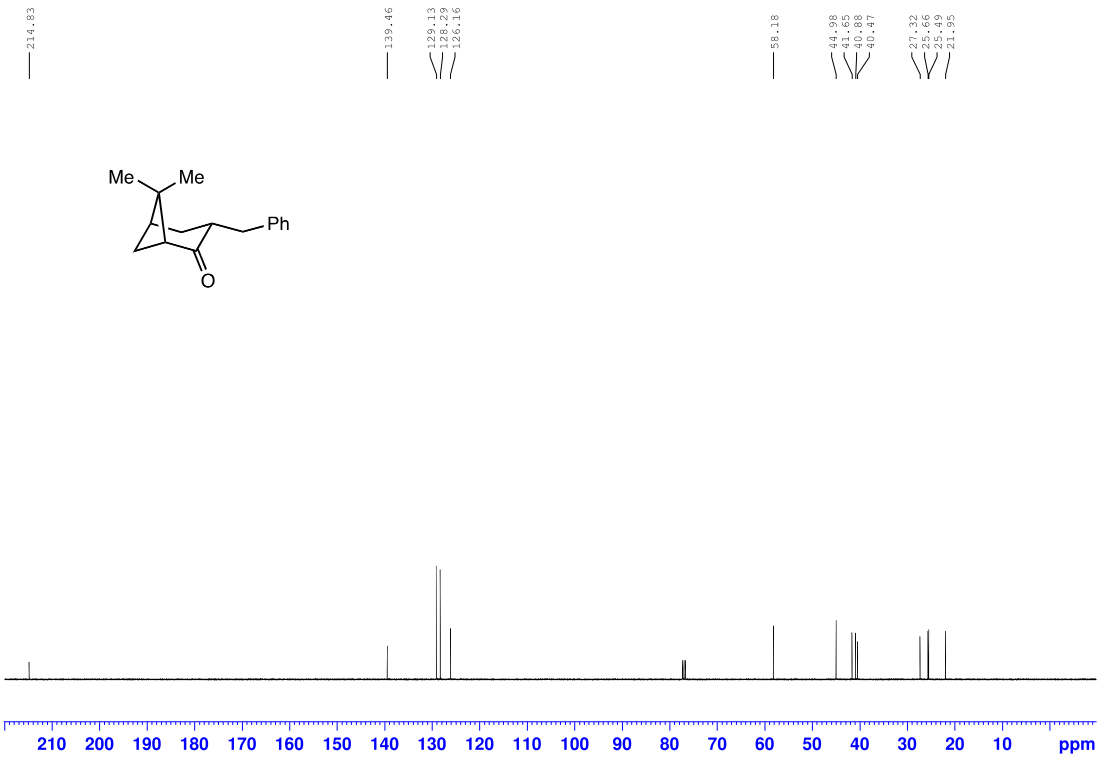

Fig. S57. ^{13}C NMR spectrum ($CDCl_3$, 100 MHz) of starting material for compound **5**.

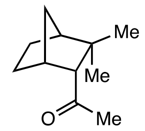

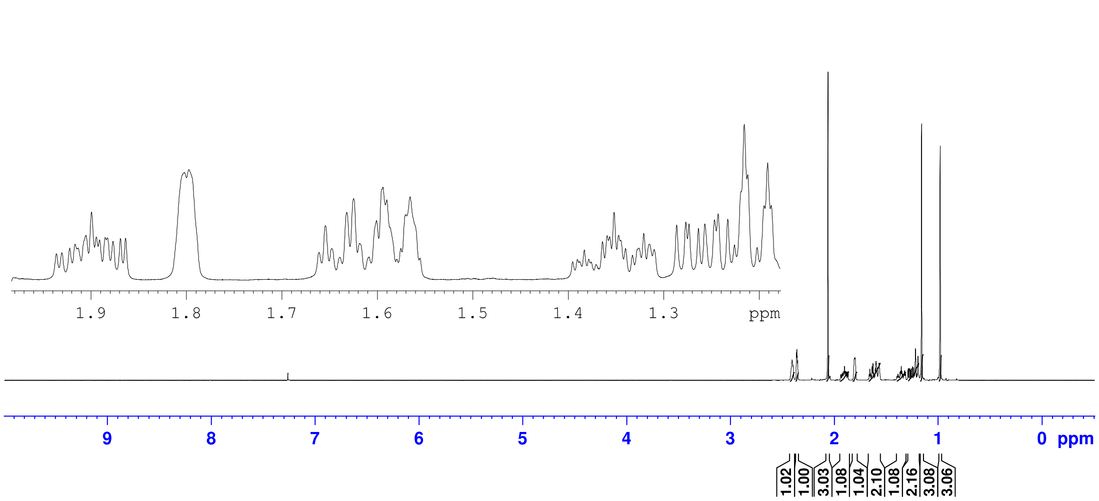

Fig. S58. ^{1}H NMR spectrum ($CDCl_3$, 400 MHz) of starting material for compound **6**.

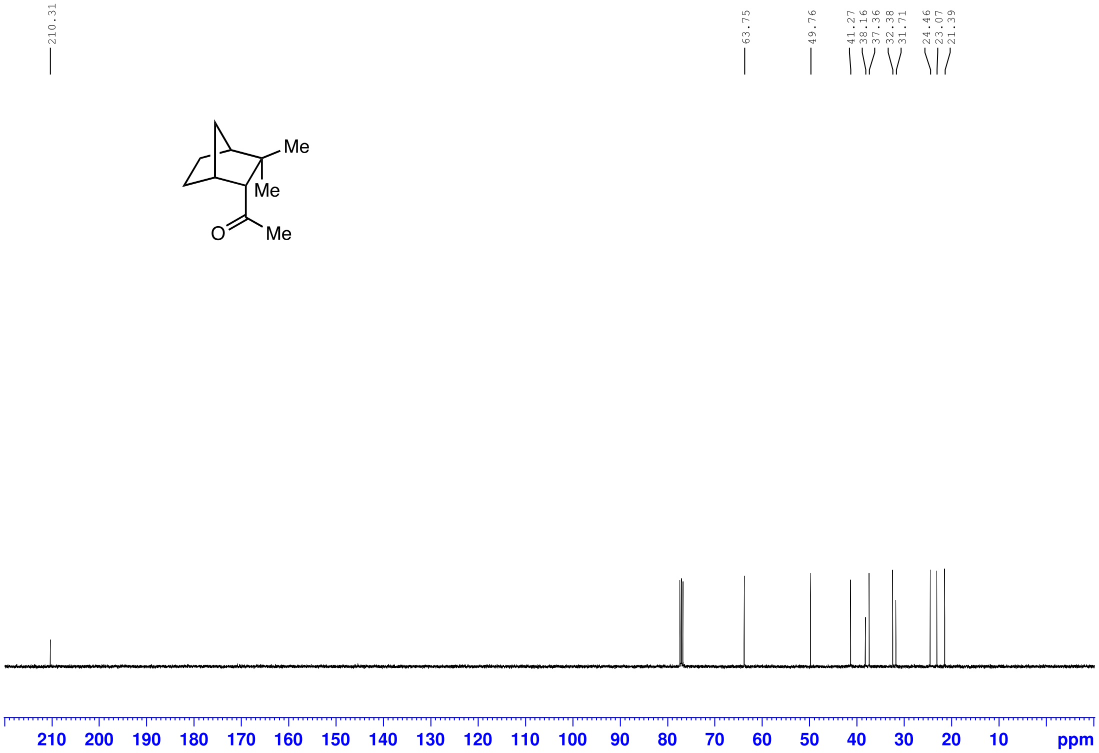

Fig. S59. ^{13}C NMR spectrum ($CDCl_3$, 100 MHz) of starting material for compound **6**.

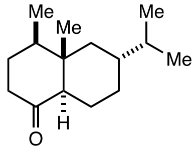

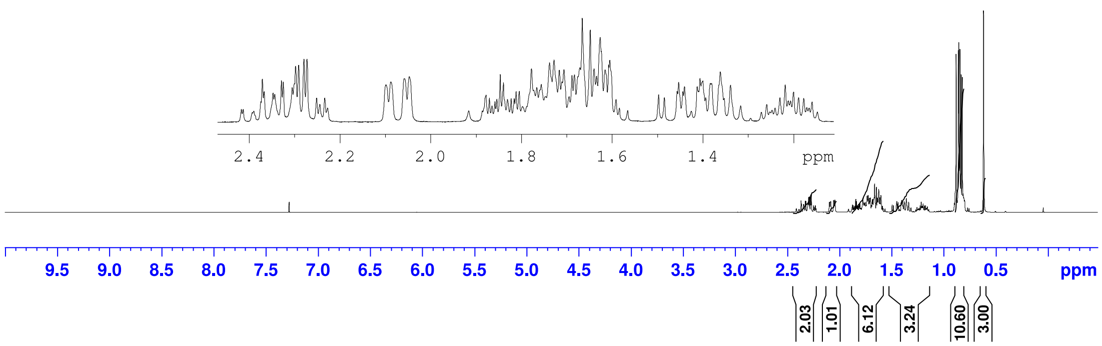

Fig. S60. ^{1}H NMR spectrum ($CDCl_3$, 400 MHz) of starting material for compound **7**.

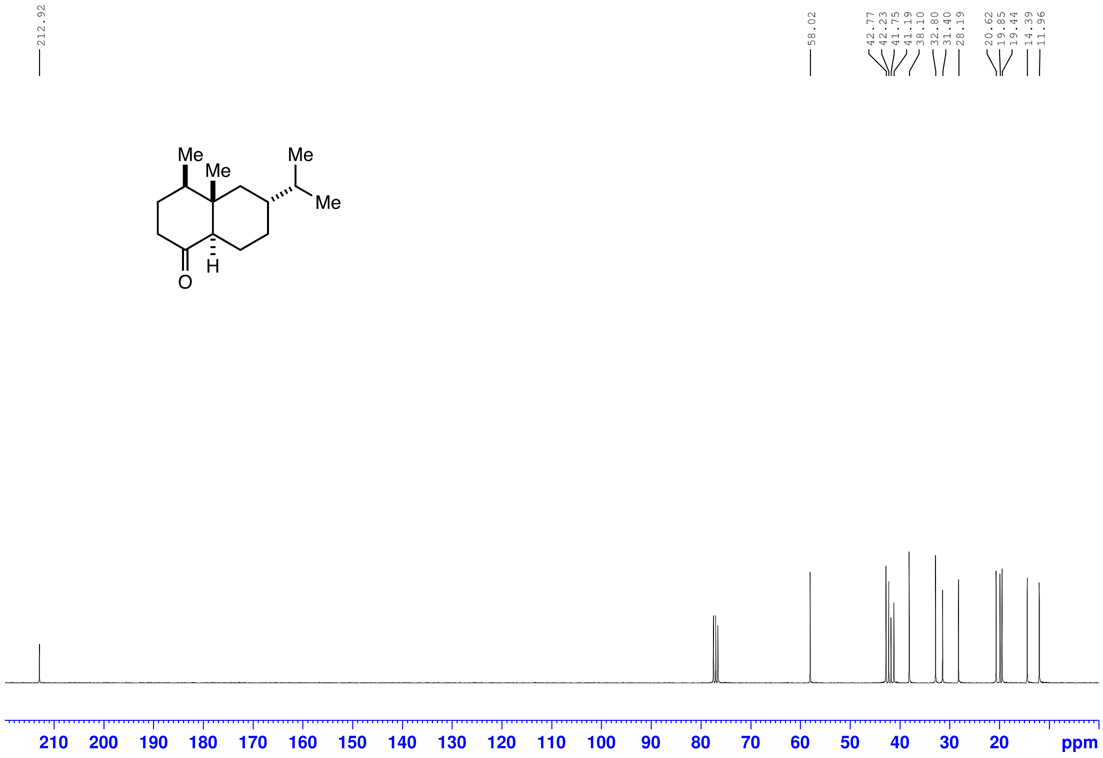

Fig. S61. ^{13}C NMR spectrum ($CDCl_3$, 100 MHz) of starting material for compound **7**.

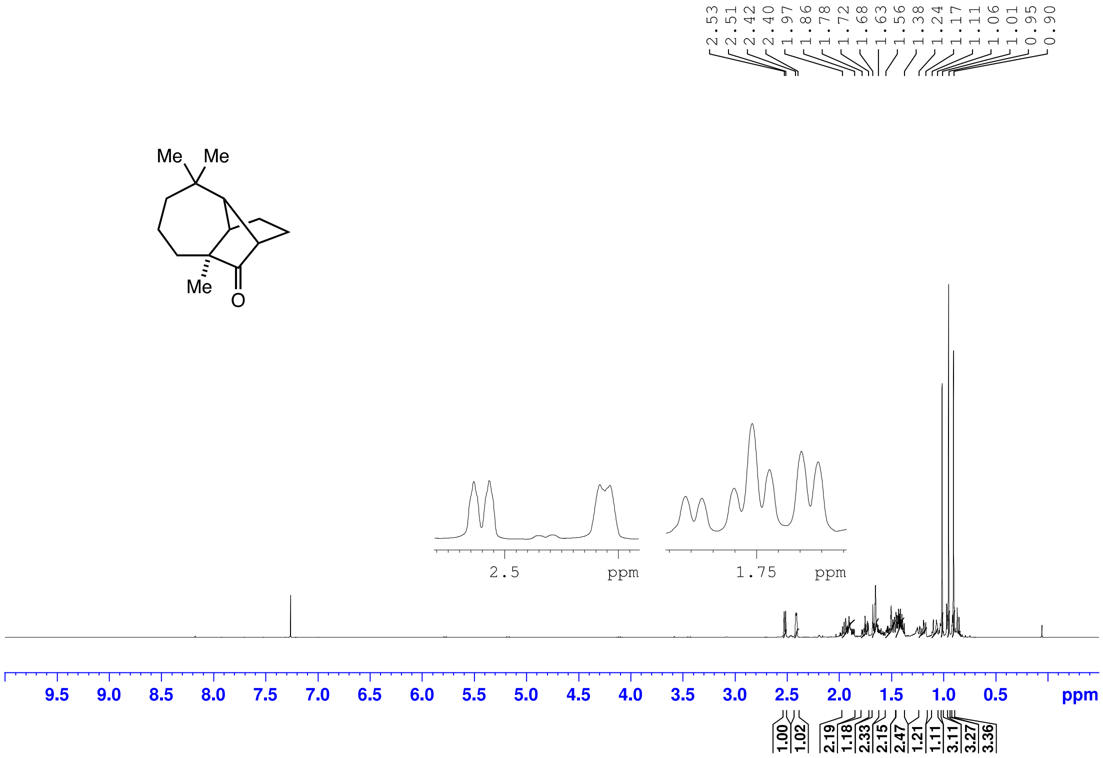

Fig. S62. ^{1}H NMR spectrum ($CDCl_3$, 400 MHz) of starting material for compound **8**.

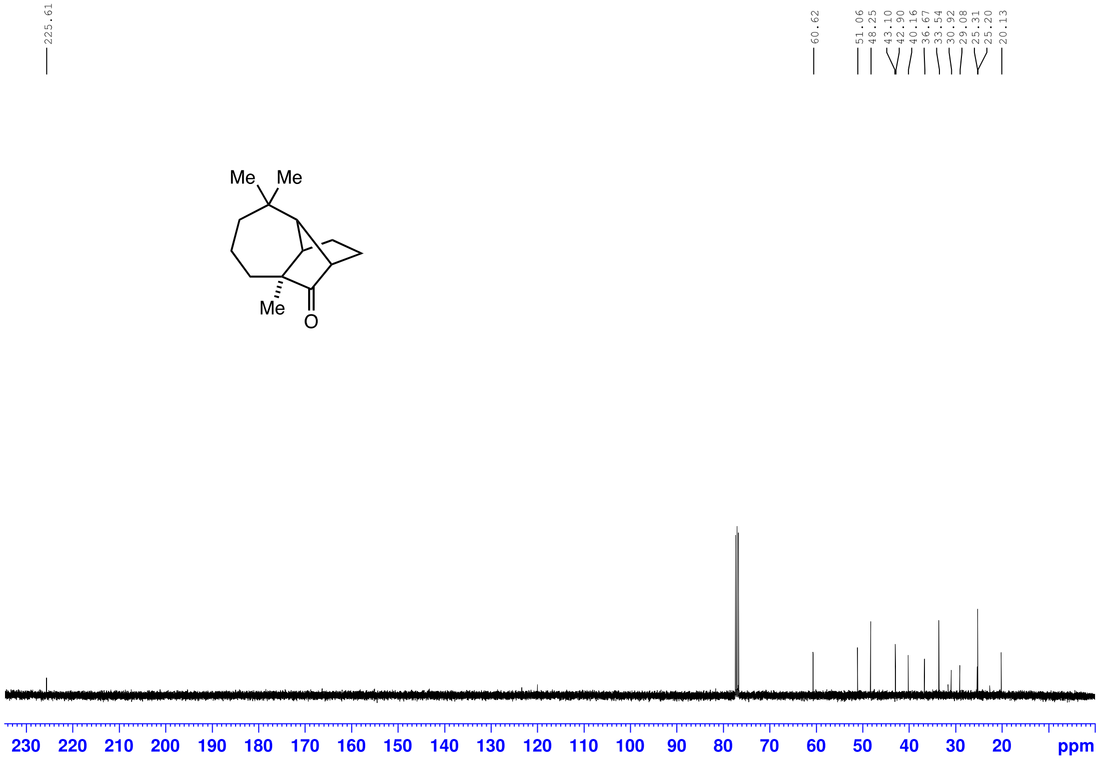

Fig. S63. ^{13}C NMR spectrum ($CDCl_3$, 100 MHz) of starting material for compound **8**.

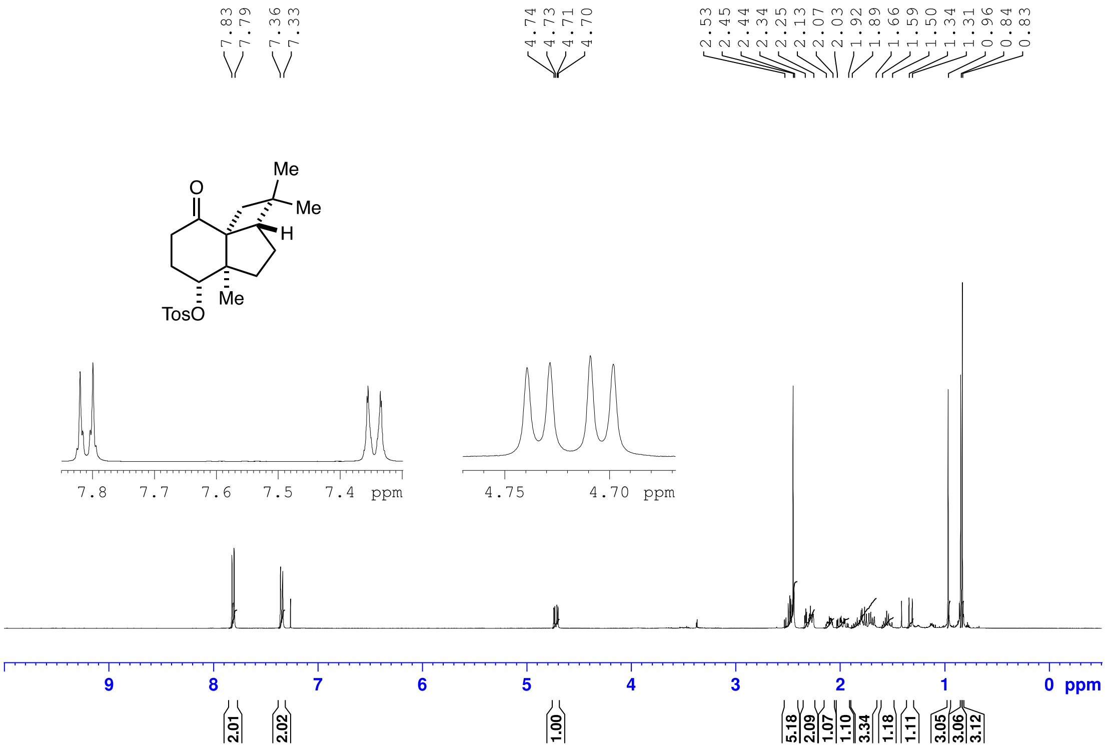

Fig. S64. ^{1}H NMR spectrum ($CDCl_3$, 400 MHz) of starting material for compound **9**.

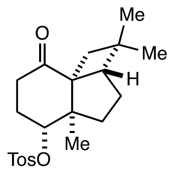

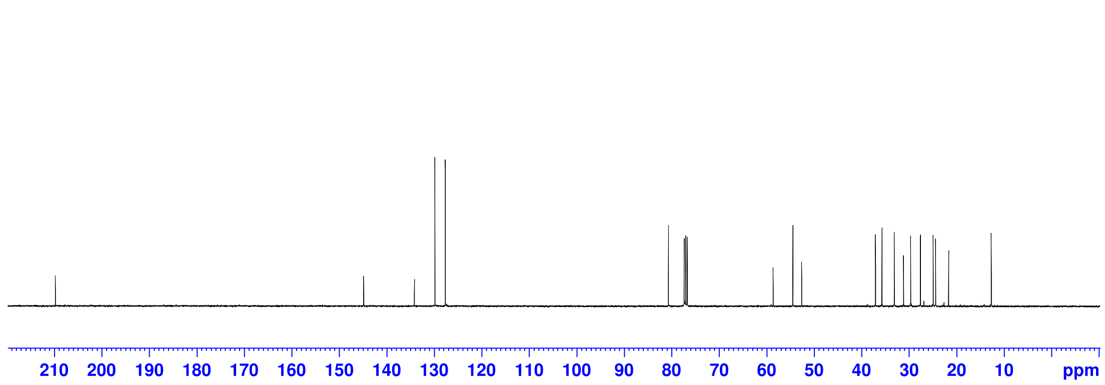

Fig. S65. ^{13}C NMR spectrum ($CDCl_3$, 100 MHz) of starting material for compound **9**.

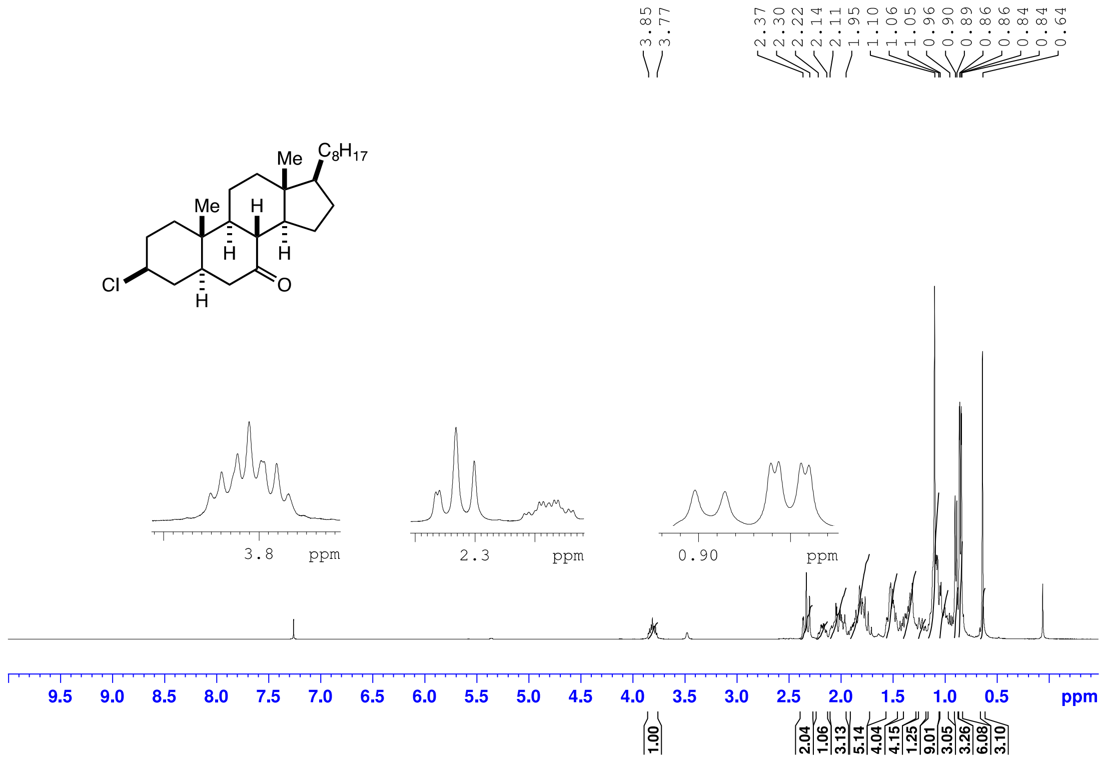

Fig. S66. ^{1}H NMR spectrum ($CDCl_3$, 400 MHz) of starting material for compound **10**.

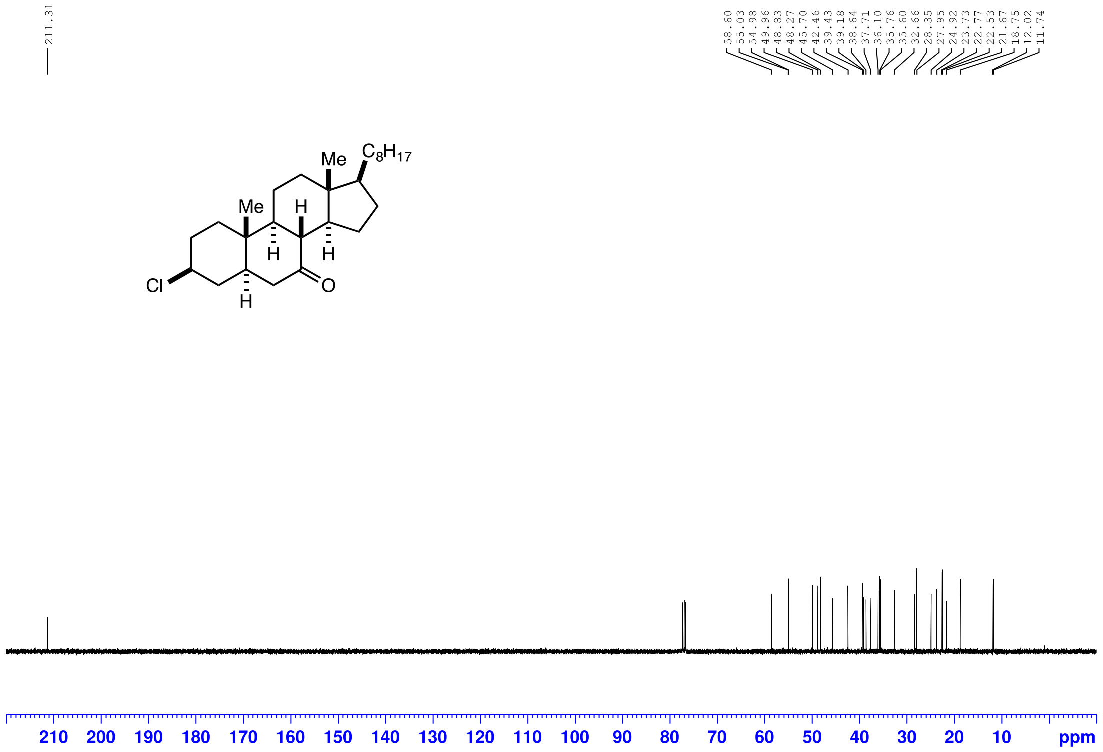

Fig. S67. ^{13}C NMR spectrum ($CDCl_3$, 100 MHz) of starting material for compound **10**.

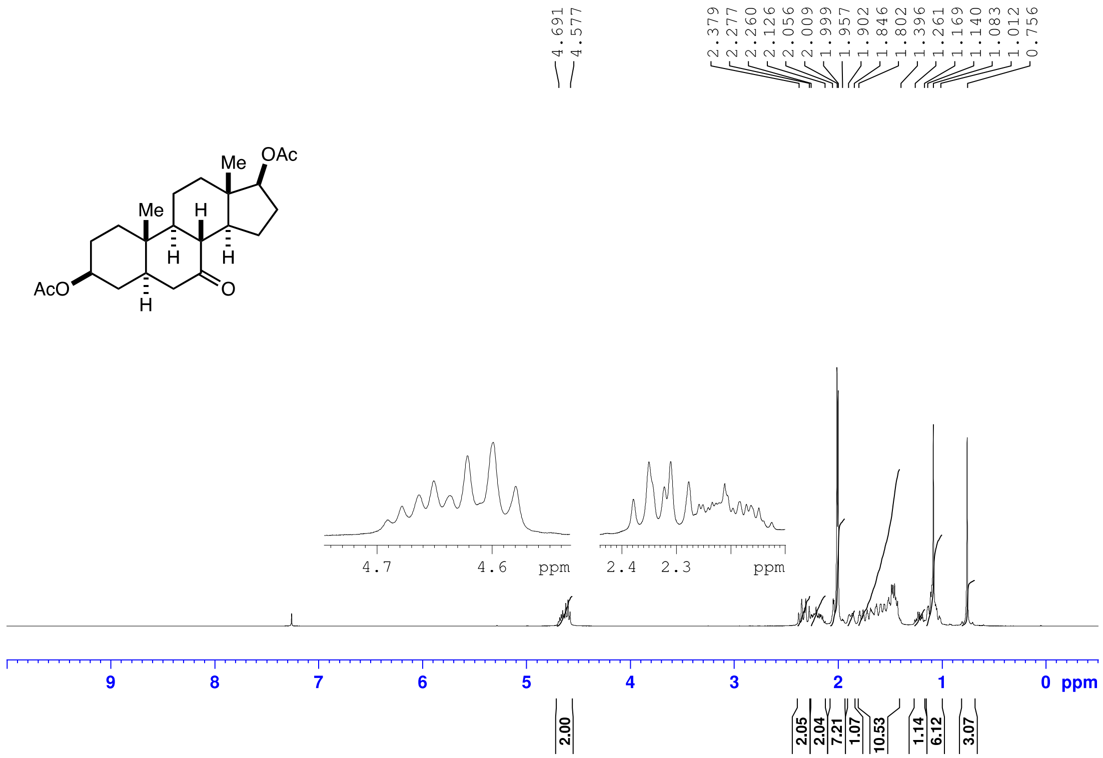

Fig. S68. ^{1}H NMR spectrum ($CDCl_3$, 400 MHz) of starting material for compound **11**.

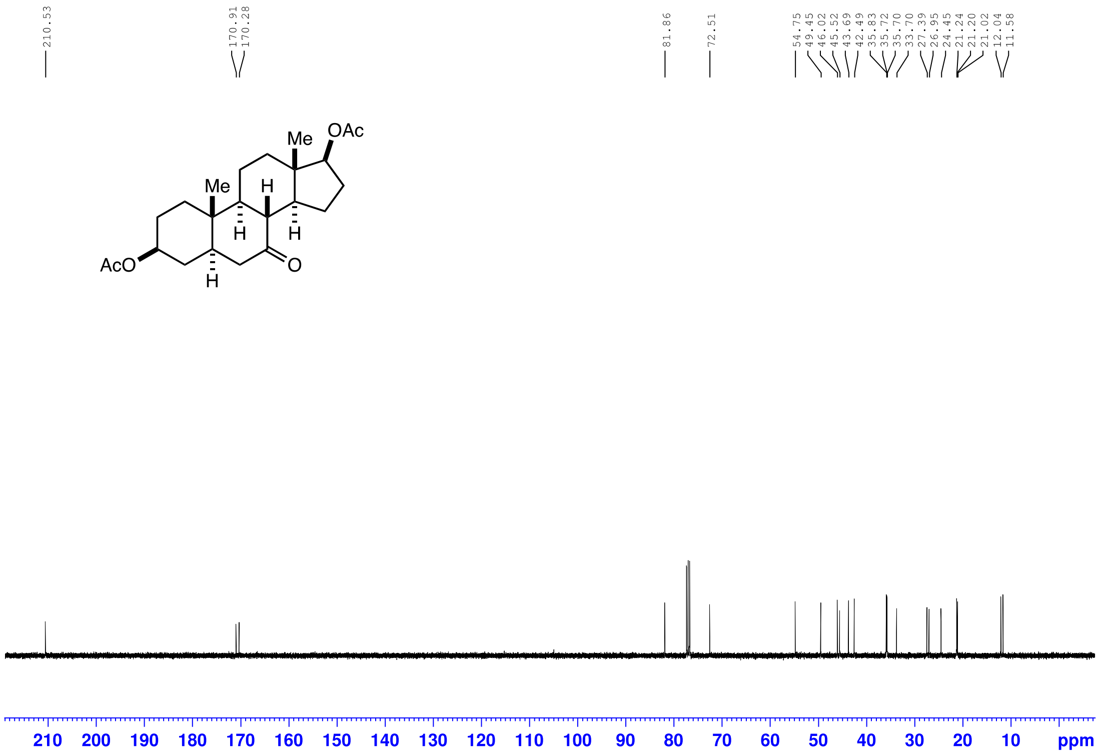

Fig. S69. ^{13}C NMR spectrum ($CDCl_3$, 100 MHz) of starting material for compound **11**.

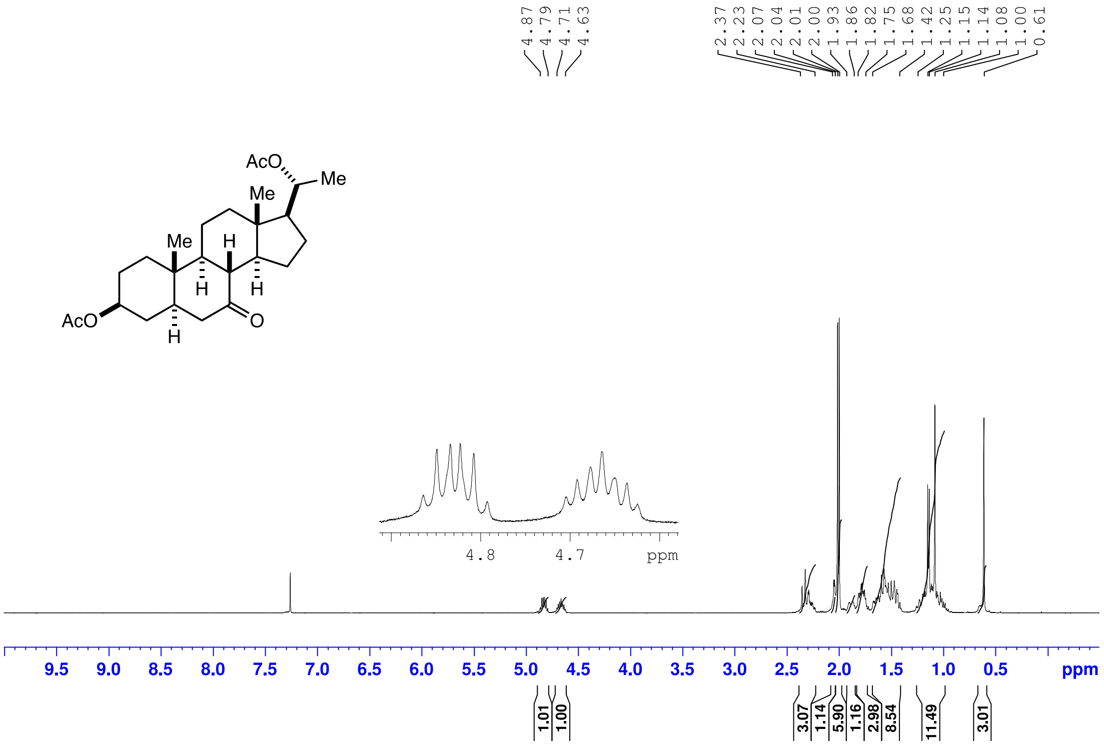

Fig. S70. ^{1}H NMR spectrum ($CDCl_3$, 400 MHz) of starting material for compound **12**.

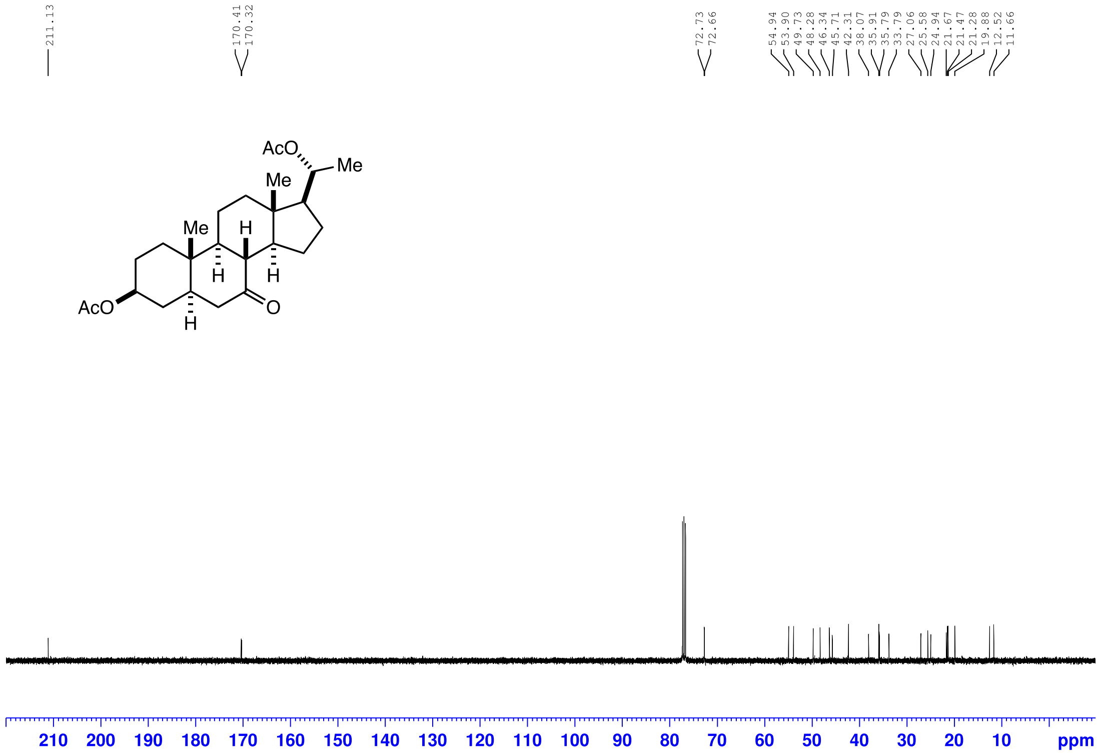

Fig. S71. ^{13}C NMR spectrum ($CDCl_3$, 100 MHz) of starting material for compound **12**.

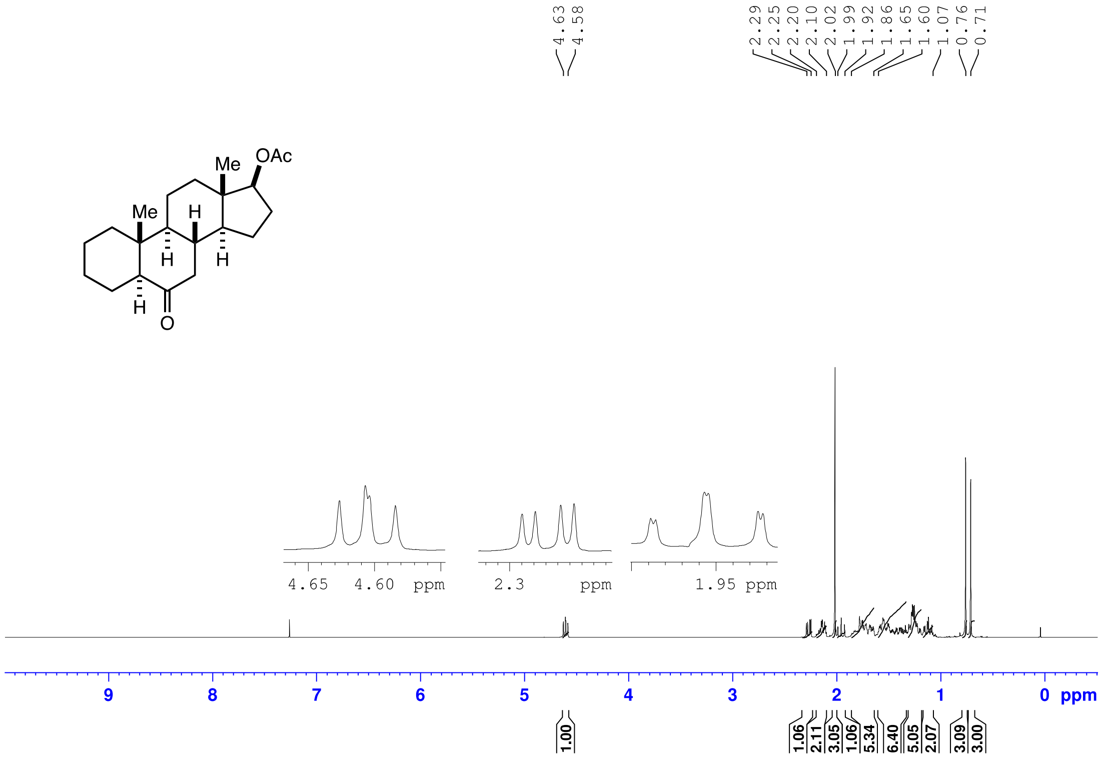

Fig. S72. ^{1}H NMR spectrum ($CDCl_3$, 400 MHz) of starting material for compound **13**.

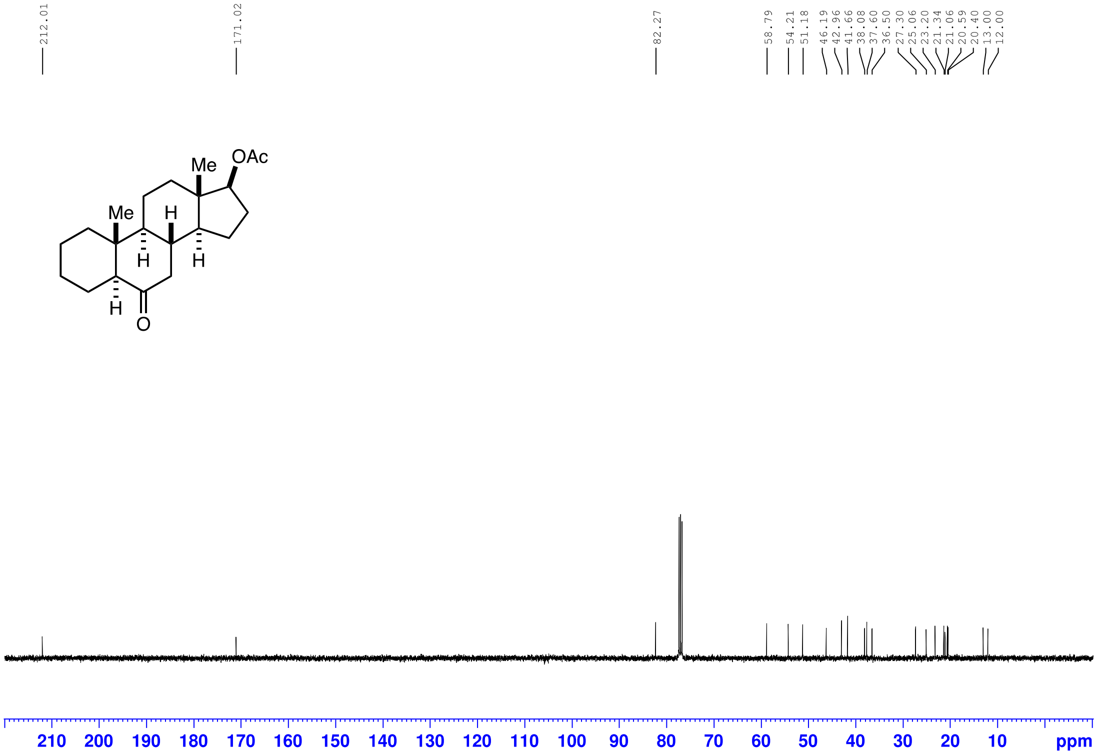

Fig. S73. ^{13}C NMR spectrum ($CDCl_3$, 100 MHz) of starting material for compound **13**.

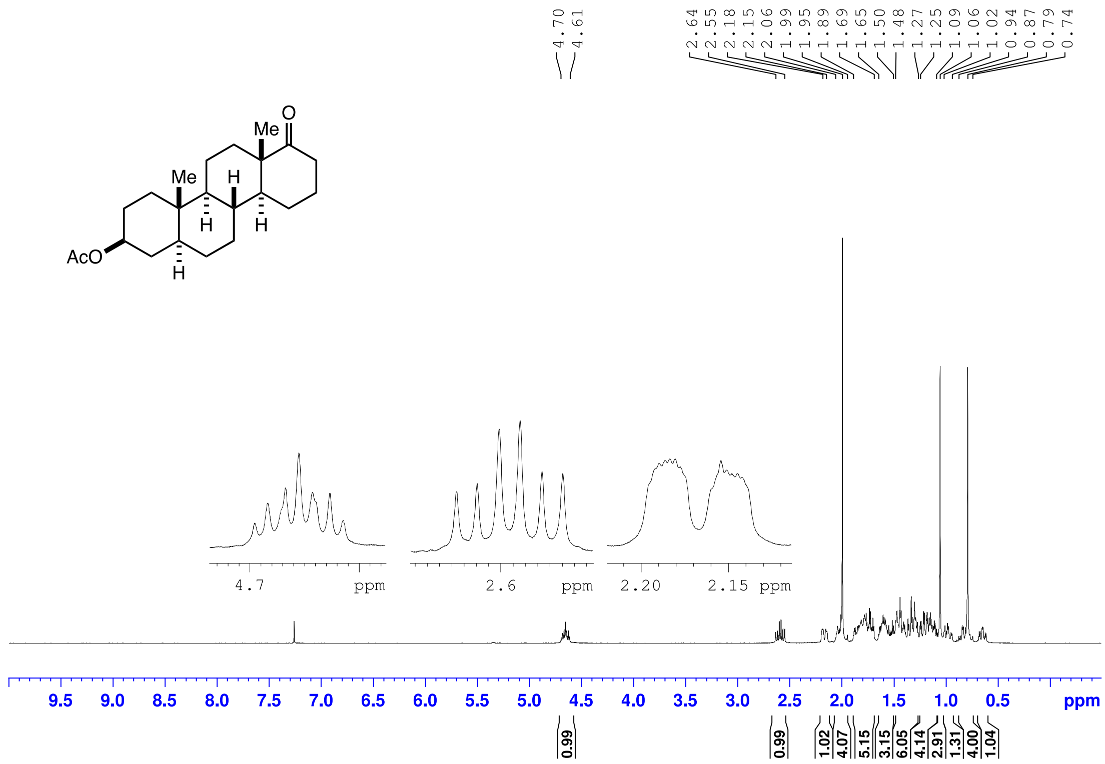

Fig. S74. ^{1}H NMR spectrum ($CDCl_3$, 400 MHz) of starting material for compound **14**.

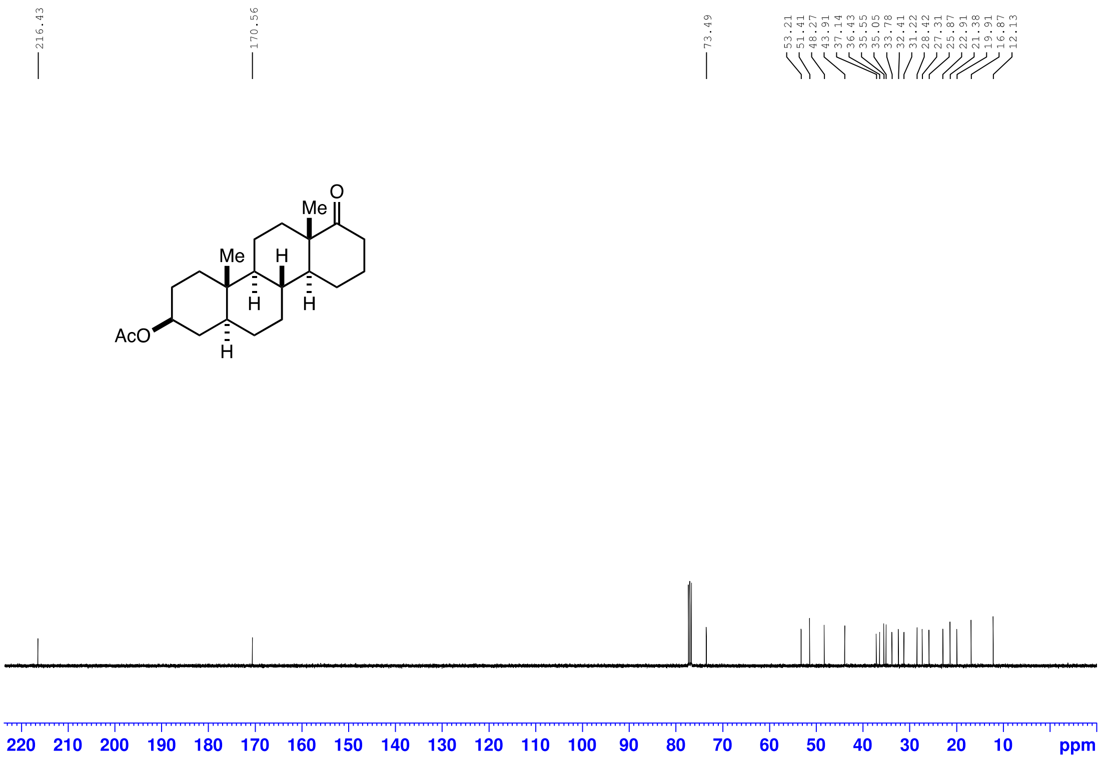

Fig. S75. ^{13}C NMR spectrum ($CDCl_3$, 100 MHz) of starting material for compound **14**.

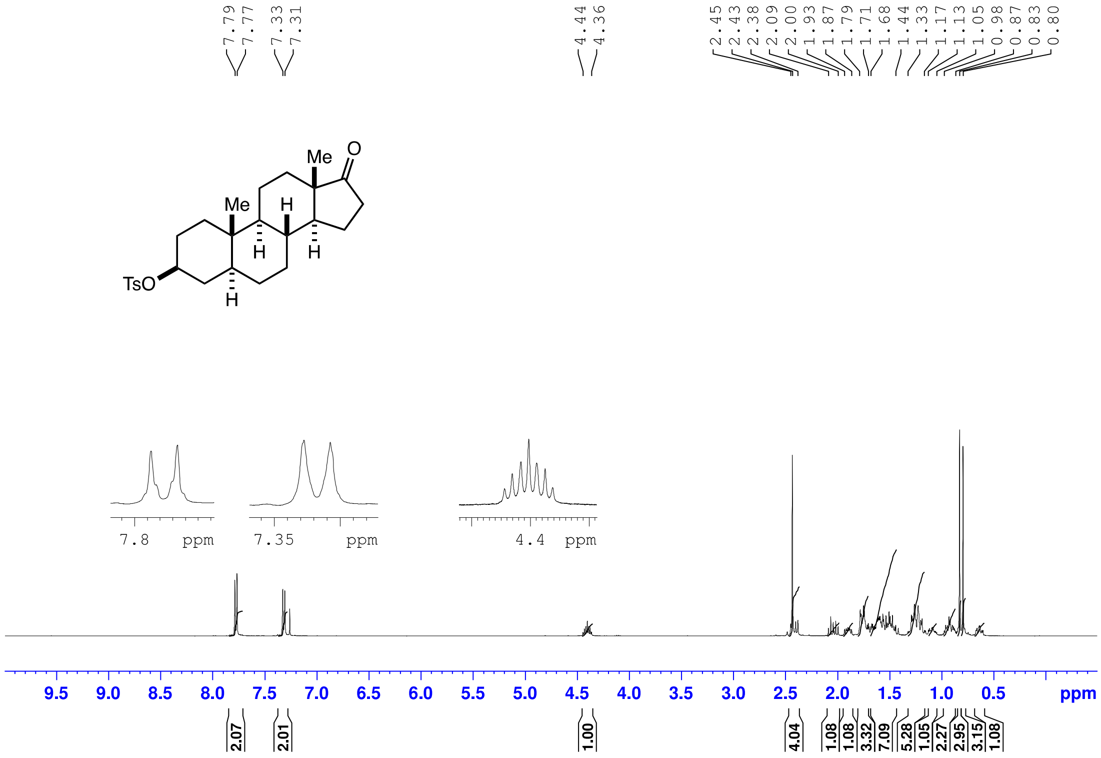

Fig. S76. ^{1}H NMR spectrum ($CDCl_3$, 400 MHz) of starting material for compound **15**.

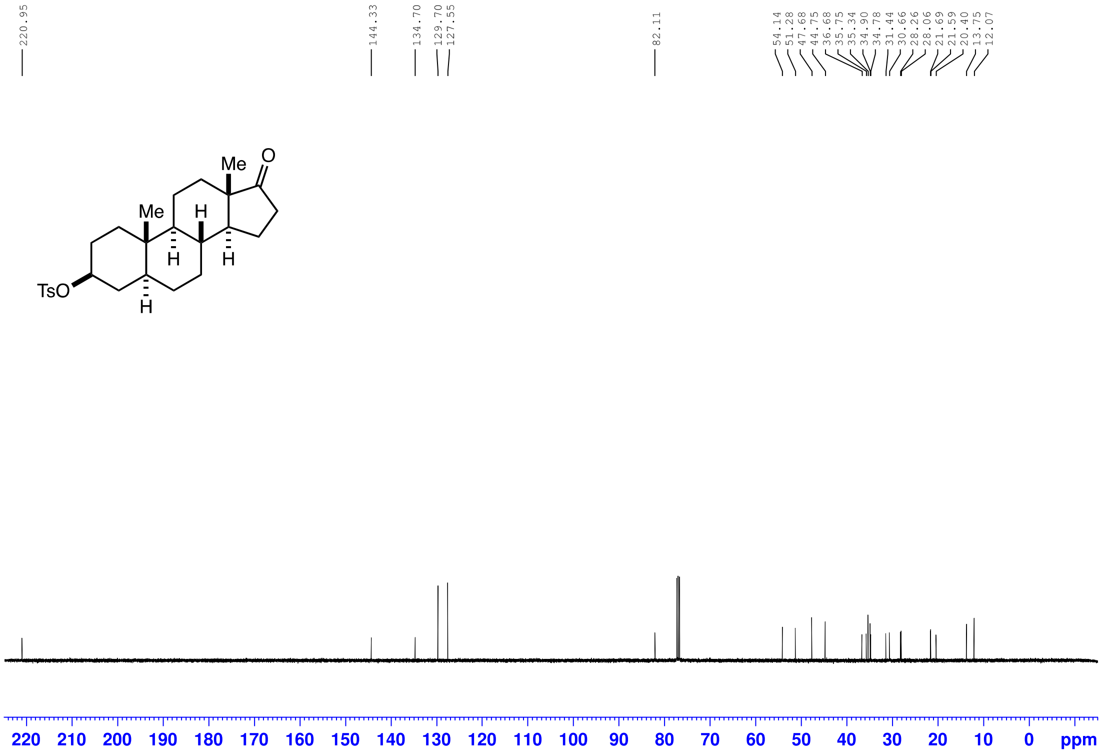

Fig. S77. ^{13}C NMR spectrum ($CDCl_3$, 100 MHz) of starting material for compound **15**.

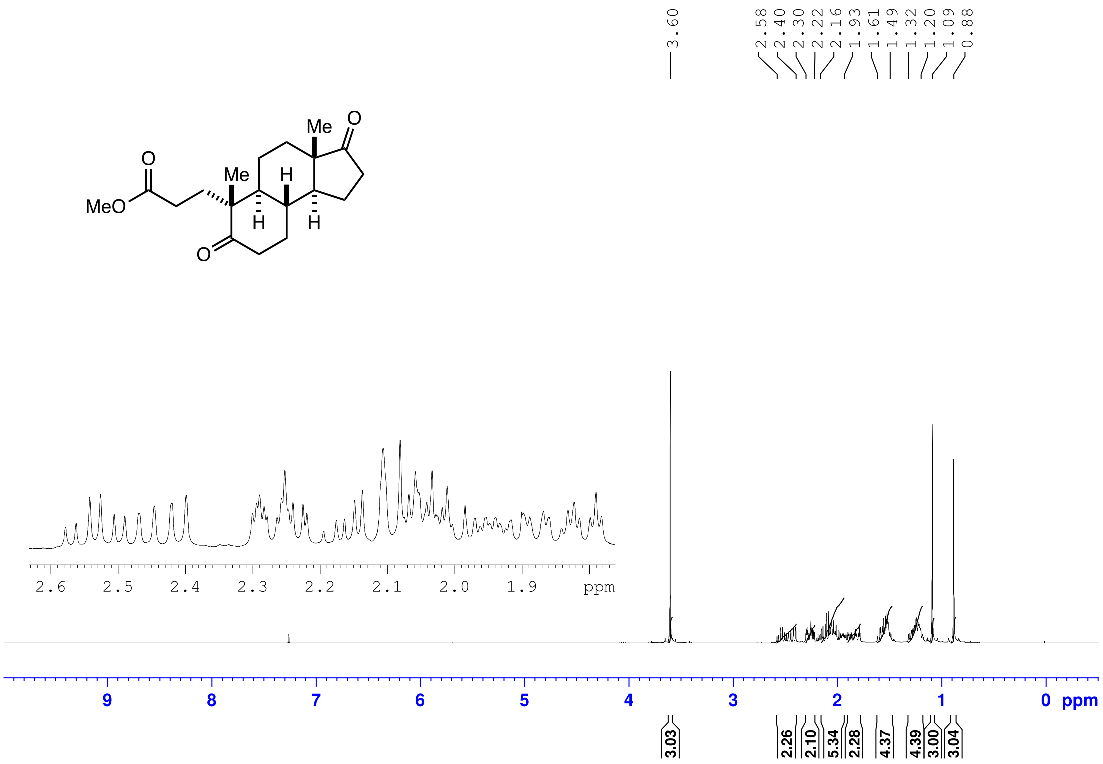

Fig. S78. ^{1}H NMR spectrum ($CDCl_3$, 400 MHz) of starting material for compound **16**.

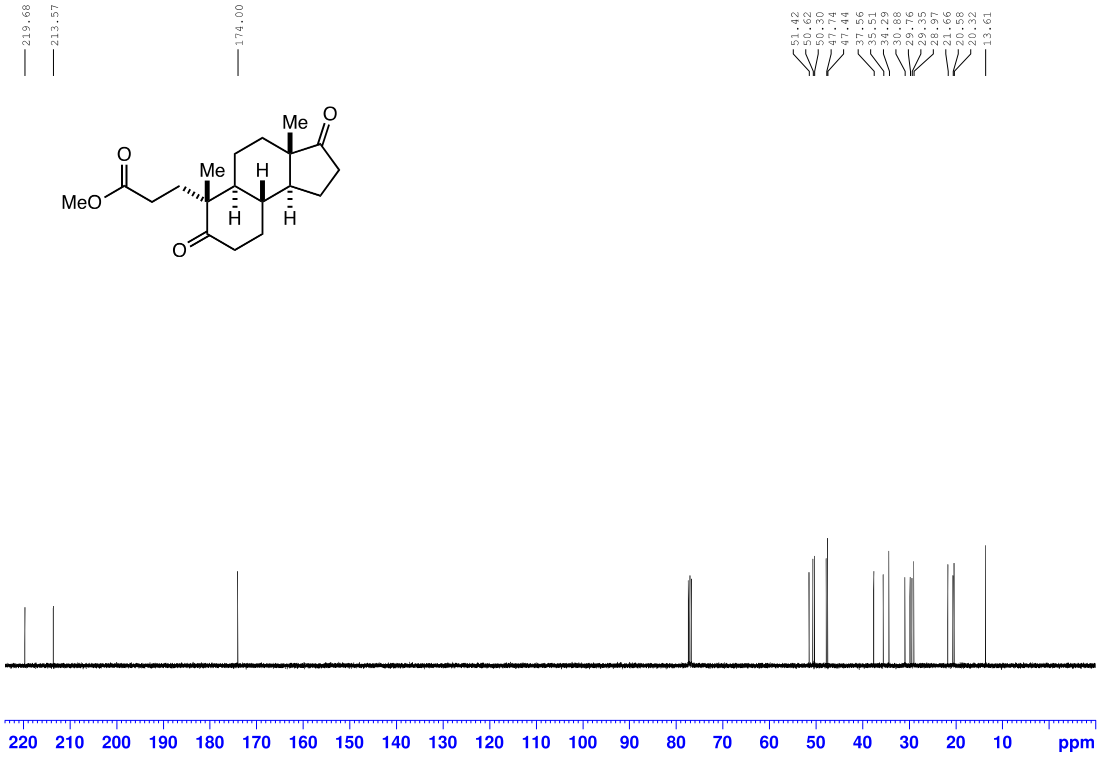

Fig. S79. ^{13}C NMR spectrum ($CDCl_3$, 100 MHz) of starting material for compound **16**.

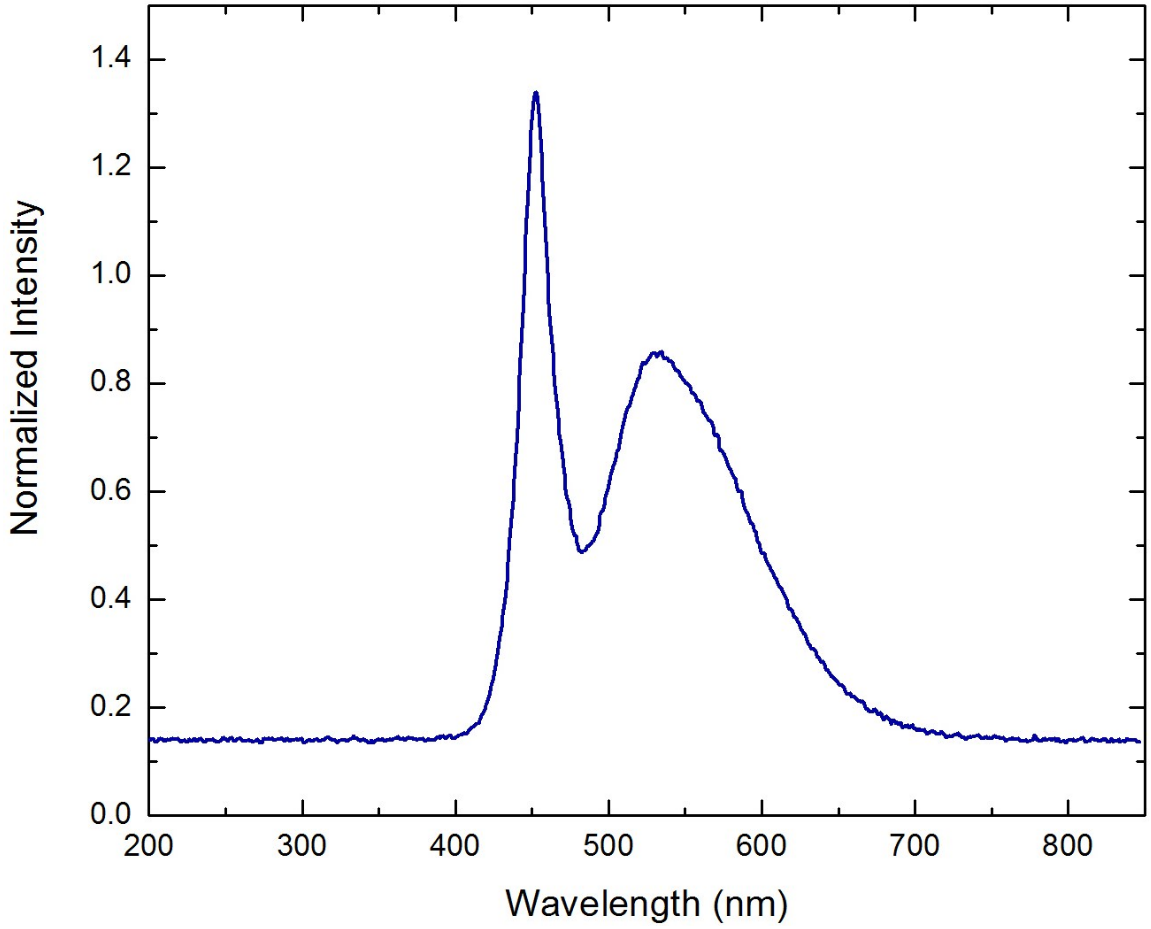

Fig. S80. UV-vis spectrum of cool white LED light source.

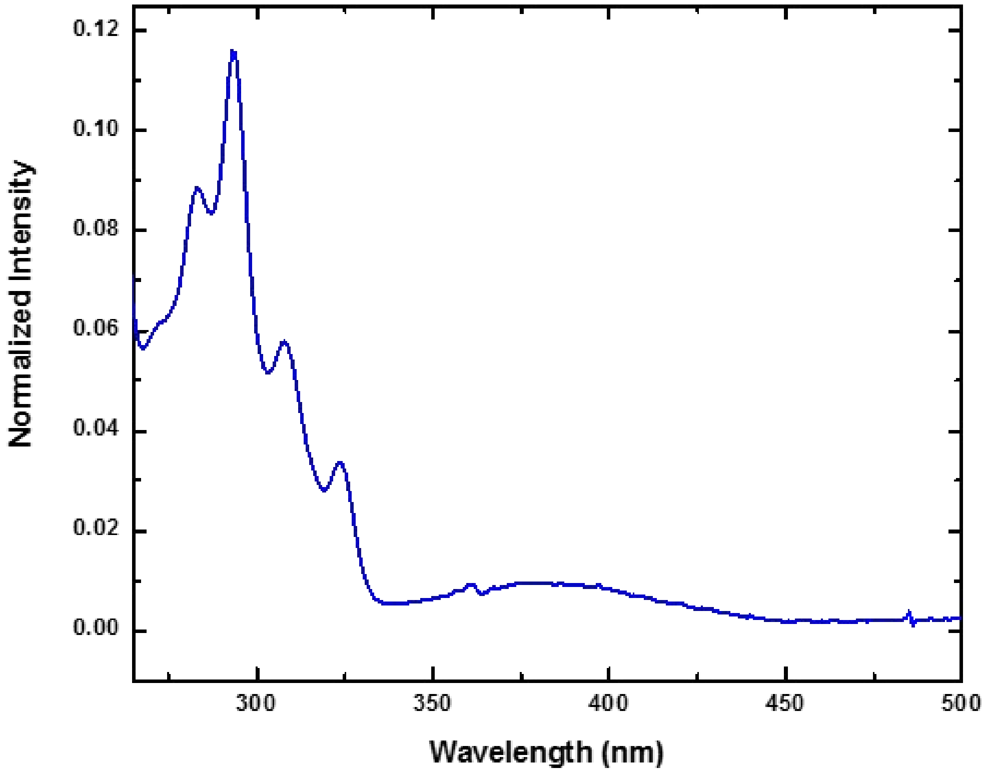

Fig. S81. UV-vis spectrum of 9-fluorenone in MeCN.

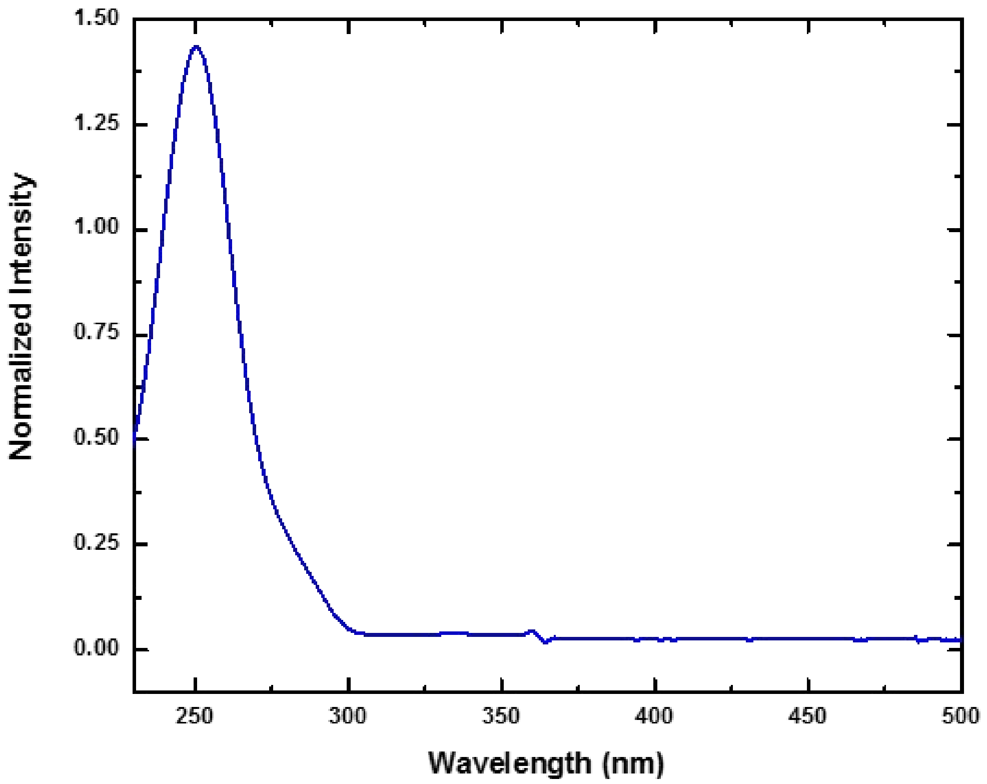

Fig. S82. UV-vis spectrum of benzophenone in MeCN.

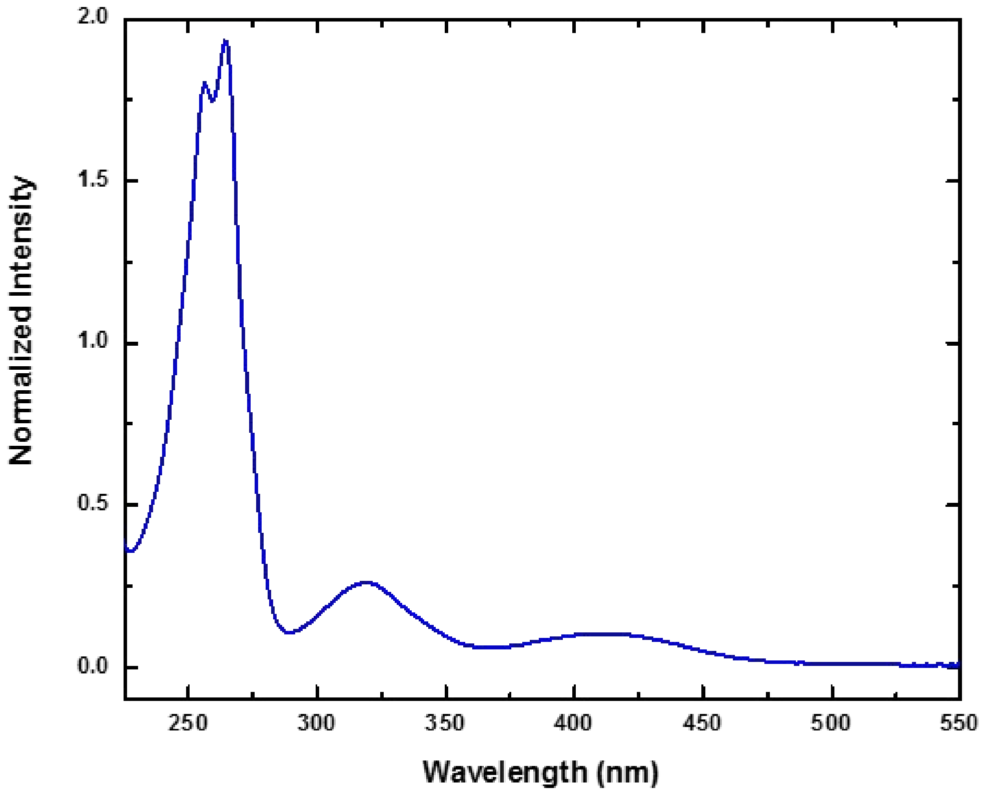

Fig. S83. UV-vis spectrum of 9,10-phenanthrenequinone in MeCN.

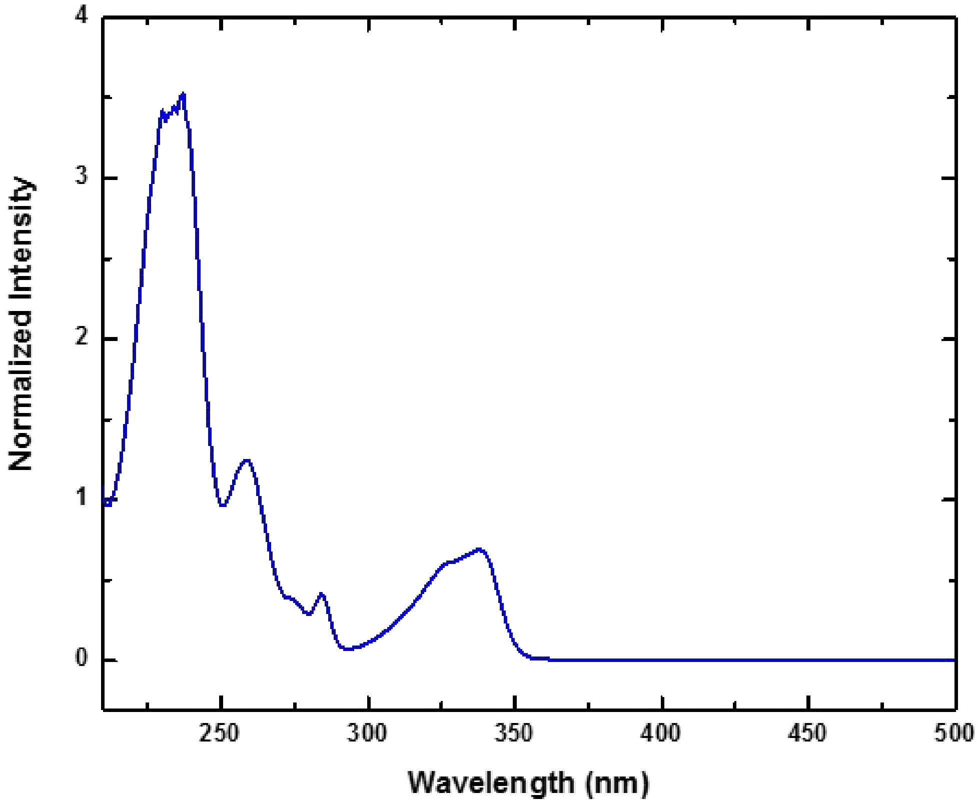

Fig. S84. UV-vis spectrum of xanthone in MeCN.

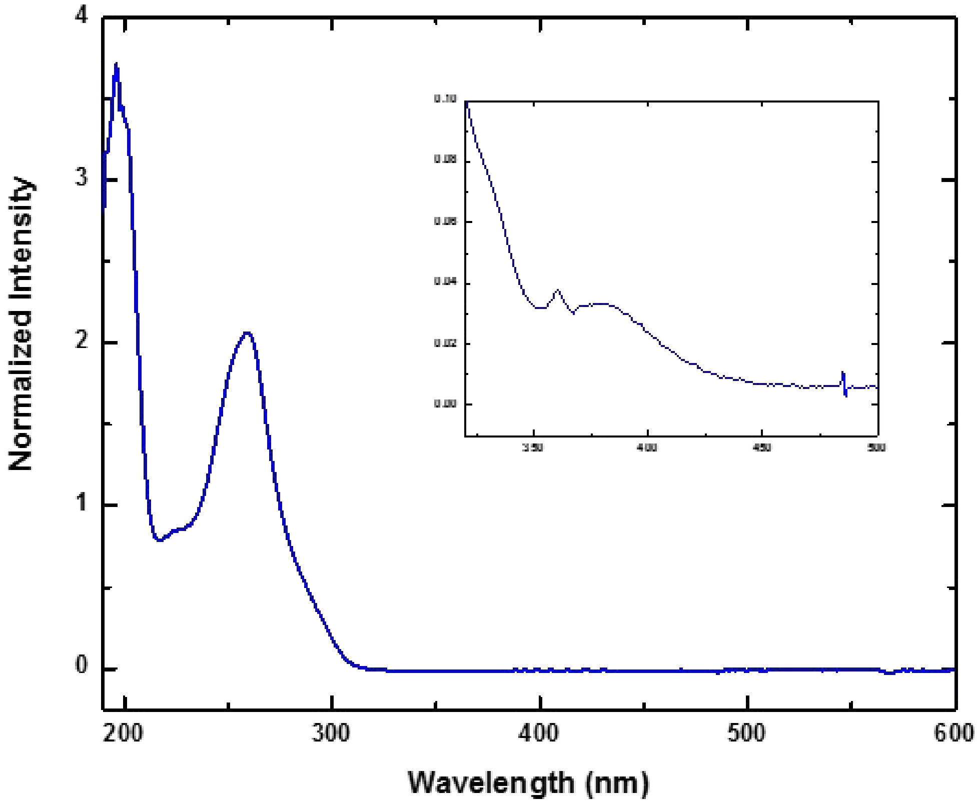

Fig. S85. UV-vis spectrum of benzil in MeCN.

Single Crystal X-ray Crystallography

All reflection intensities were measured at 110(2) (**12** and **12_minor**) or at 200(2) K (**17_18**)* using a SuperNova diffractometer (equipped with Atlas detector) with Cu *K*α radiation (λ = 1.54178 Å) under the program CrysAlisPro (Versions 1.171.36.32 or 1.171.38.43f, Rigaku Oxford Diffraction, 2013-2015). The same program was used to refine the cell dimensions and for data reduction. The structure was solved with the program SHELXS-2014/7 (Sheldrick, 2015) and was refined on F^2 with SHELXL-2014/7 (Sheldrick, 2015). Analytical numeric absorption correction using a multifaceted crystal model was applied using CrysAlisPro. The temperature of the data collection was controlled using the system Cryojet (manufactured by Oxford Instruments). The H atoms were placed at calculated positions using the instructions AFIX 13, AFIX 23 or AFIX 137 with isotropic displacement parameters having values 1.2 or 1.5 *U*eq of the attached C atoms. All important crystallographic data for **12**, **12_minor** and **17_18** are provided in Tables S1-S3.

12: The structure is ordered. The asymmetric unit contains four crystallographically independent molecules (*Z'* = 4). The structure is pseudomerohedrally twinned, and the twin relationship corresponds to a twofold axis along [1 0 2]. The BASF scale factor refines to 0.4202(14). The absolute configuration was established by anomalous-dispersion effects in diffraction measurements on the crystal. The Flack parameter refines to -0.06(5).

12_minor: The structure is partly disordered. The asymmetric unit contains four crystallographically independent molecules (*Z'* = 4). The fragments C20→C23 for molecules A and D are disordered over two orientations, and the occupancy factors of the major components of the disorder refine to 0.58(4) and 0.53(4). The crystal that was mounted on the diffractometer was twinned. The twin relationship corresponds to a twofold axis along the 0.8386a* + 0.4189b* + 0.3481c* reciprocal direction. The BASF scale factor refines to 0.2891(15). The absolute configuration was established by anomalous-dispersion effects in diffraction measurements on the crystal. The Flack parameter refines to 0.01(7).

18_19: The structure is disordered as the crystal contains a phase mixture of two different isomers for which the F atom can be positioned either on C12 (minor component) or on C16 (major component). The occupancy factor of the major component of the disorder refines to 0.643(5). The same observations were made by NMR. The absolute configuration could not be established by anomalous-dispersion effects in diffraction measurements on the crystal.

* A solid-solid phase transition occurs between 200 and 110 K. Data at 110 K suggested some significant anomalous diffuse scattering, and the refinement of the low-T phase was not acceptable.

Table S1. Experimental details for compound **12**.

	12
Crystal data	
Chemical formula	$C_{25}H_{37}FO_5$
M_r	436.54
Crystal system, space group	Monoclinic, $P2_1$
Temperature (K)	110
a, *b*, *c* (Å)	14.1167 (2), 11.44999 (15), 29.4125 (4)
□ (°)	103.8206 (15)
V (Å^3)	4616.48 (11)
Z	8
Radiation type	Cu K□
□ (mm^{-1})	0.74
Crystal size (mm)	0.48 × 0.37 × 0.05
Data collection	
Diffractometer	SuperNova, Dual, Cu at zero, Atlas
Absorption correction	Analytical<br>*CrysAlis PRO*, Agilent Technologies, Version 1.171.36.32 (release 02-08-2013 CrysAlis171 .NET) (compiled Aug 2 2013,16:46:58) Analytical numeric absorption correction using a multifaceted crystal model based on expressions derived by R.C. Clark & J.S. Reid. (Clark, R. C. & Reid, J. S. (1995). Acta Cryst. A51, 887-897)
T_{min}, T_{max}	0.783, 0.964
No. of measured, independent and observed [I > 2□(I)] reflections	58677, 18040, 17415
R_{int}	0.037
(sin □/□)$_{max}$ (Å^{-1})	0.616
Refinement	
R[F^2 > 2□(F^2)], wR(F^2), S	0.041, 0.109, 1.01
No. of reflections	18040
No. of parameters	1138
No. of restraints	1
H-atom treatment	H-atom parameters constrained
□ρ$_{max}$, □ρ$_{min}$ (e Å^{-3})	0.19, -0.20

Absolute structure	Flack x determined using 7774 quotients [(I+)-(I-)]/[(I+)+(I-)] (Parsons, Flack and Wagner, Acta Cryst. B69 (2013) 249-259).
Absolute structure parameter	-0.06 (5)

Computer programs: *CrysAlis PRO*, Agilent Technologies, Version 1.171.36.32 (release 02-08-2013 CrysAlis171 .NET) (compiled Aug 2 2013, 16:46:58), *SHELXS2014*/7 (Sheldrick, 2015), *SHELXL2014*/7 (Sheldrick, 2015), *SHELXTL* v6.10 (Sheldrick, 2008).[23]

Table S2. Experimental details for compound **12_minor**.

	12_minor
Crystal data	
Chemical formula	$C_{25}H_{37}FO_5$
M_r	436.54
Crystal system, space group	Triclinic, *P*1
Temperature (K)	110
a, *b*, *c* (Å)	6.62820 (13), 12.7871 (2), 29.3443 (5)
□, □, □ (°)	81.5019 (14), 84.6748 (15), 75.0869 (16)
V ($Å^3$)	2373.02 (7)
Z	4
Radiation type	Cu *K*□
□ (mm^{-1})	0.72
Crystal size (mm)	0.33 × 0.27 × 0.10
Data collection	
Diffractometer	SuperNova, Dual, Cu at zero, Atlas
Absorption correction	Analytical<br>*CrysAlis PRO* 1.171.38.43f (Rigaku Oxford Diffraction, 2015) Analytical numeric absorption correction using a multifaceted crystal model based on expressions derived by R.C. Clark & J.S. Reid. (Clark, R. C. & Reid, J. S. (1995). Acta Cryst. A51, 887-897) Empirical absorption correction using spherical harmonics, implemented in SCALE3 ABSPACK scaling algorithm.
T_{min}, T_{max}	0.833, 0.938
No. of measured, independent and observed [I > 2□(I)] reflections	52212, 19284, 18138
R_{int}	0.022
(sin □/□)$_{max}$ ($Å^{-1}$)	0.617
Refinement	
$R[F^2$ > 2□(F^2)], $wR(F^2)$, S	0.038, 0.109, 1.03
No. of reflections	19284
No. of parameters	1234
No. of restraints	349
H-atom treatment	H-atom parameters constrained
□ρ$_{max}$, □ρ$_{min}$ (e $Å^{-3}$)	0.26, -0.23

Absolute structure	Flack x determined using 7634 quotients [(I+)-(I-)]/[(I+)+(I-)] (Parsons, Flack and Wagner, Acta Cryst. B69 (2013) 249-259).
Absolute structure parameter	0.01 (7)

Computer programs: *CrysAlis PRO* 1.171.38.43f (Rigaku OD, 2015), *SHELXS2014*/7 (Sheldrick, 2015), *SHELXL2014*/7 (Sheldrick, 2015), *SHELXTL* v6.10 (Sheldrick, 2008).[23]

Table S3. Experimental details for compound **17_18**.

	17_18
Crystal data	
Chemical formula	$C_{21}H_{29}FO_2$
M_r	332.44
Crystal system, space group	Orthorhombic, $P2_12_12$
Temperature (K)	200
a, *b*, *c* (Å)	12.91998 (13), 18.73733 (15), 7.59349 (7)
V (Å^3)	1838.28 (3)
Z	4
Radiation type	Cu $K\alpha$
μ (mm^{-1})	0.66
Crystal size (mm)	0.36 × 0.35 × 0.27
Data collection	
Diffractometer	SuperNova, Dual, Cu at zero, Atlas
Absorption correction	Analytical<br>*CrysAlis PRO*, Agilent Technologies, Version 1.171.36.32 (release 02-08-2013 CrysAlis171 .NET) (compiled Aug 2 2013,16:46:58) Analytical numeric absorption correction using a multifaceted crystal model based on expressions derived by R.C. Clark & J.S. Reid. (Clark, R. C. & Reid, J. S. (1995). Acta Cryst. A51, 887-897)
T_{min}, T_{max}	0.824, 0.870
No. of measured, independent and observed [$I > 2\sigma(I)$] reflections	24088, 3624, 3507
R_{int}	0.059
$(\sin \theta/\lambda)_{max}$ (Å^{-1})	0.616
Refinement	
$R[F^2 > 2\sigma(F^2)]$, $wR(F^2)$, S	0.049, 0.132, 1.03
No. of reflections	3624
No. of parameters	441
No. of restraints	862
H-atom treatment	H-atom parameters constrained
$\Delta\rho_{max}$, $\Delta\rho_{min}$ (e Å^{-3})	0.17, -0.15
Absolute structure	Refined as an inversion twin.
Absolute structure	-0.7 (4)

parameter	

Computer programs: *CrysAlis PRO*, Agilent Technologies, Version 1.171.36.32 (release 02-08-2013 CrysAlis171 .NET) (compiled Aug 2 2013, 16:46:58), *SHELXS2014*/7 (Sheldrick, 2015), *SHELXL2014*/7 (Sheldrick, 2015), *SHELXTL* v6.10 (Sheldrick, 2008).[23]

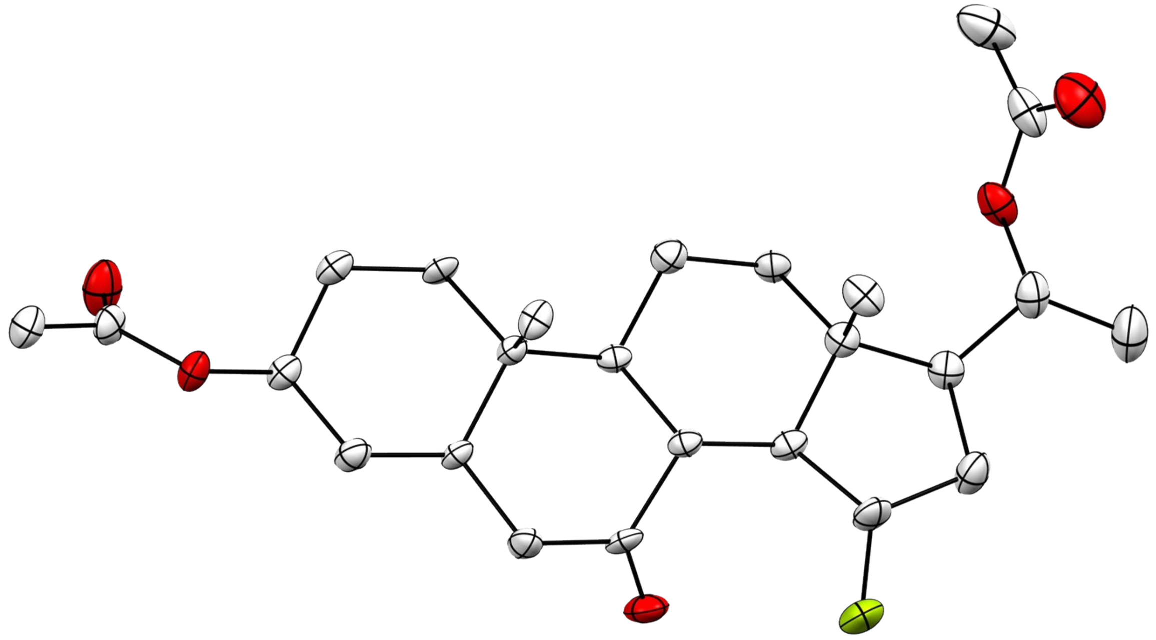

Fig. S86. ORTEP of **12** (Major Diastereomer).

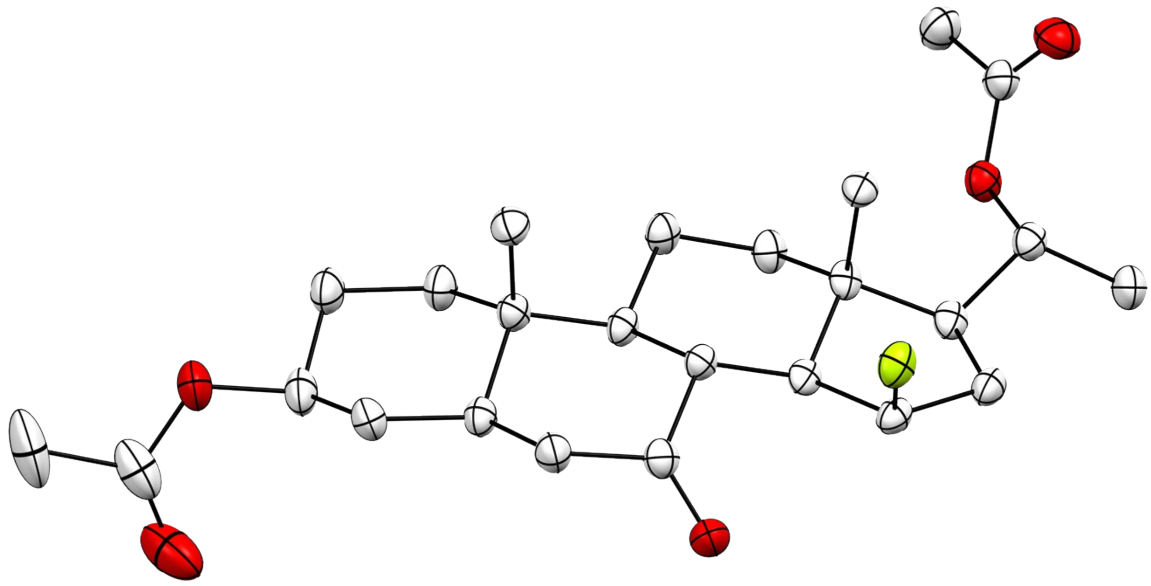

Fig. S87. ORTEP of **12** (Minor Diastereomer).

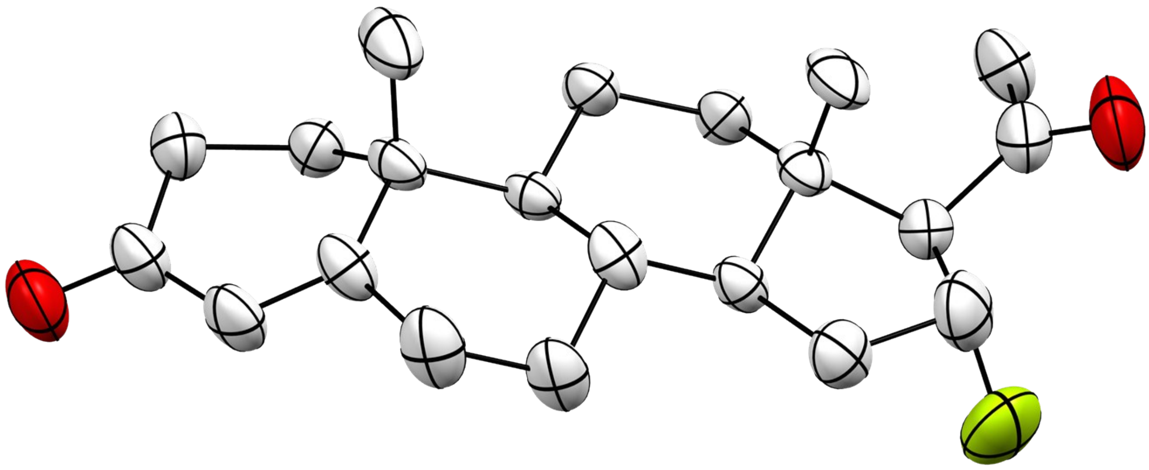

Fig. S88. ORTEP of **17_18** (Major Regioisomer).

References

[1] D. Naumann and J. Kischkewitz, *J. Fluorine Chem*., 1990, **47**, 283-299.
[2] A. Huczynski, J. Rutkowski and B. Brzezinski, *Struct. Chem.,* 2011, **22**, 627-634.
[3] B. Siewert, J. Wiemann, A. Köwitsch and R. Csuk, *Eur. J. Med. Chem.,* 2014, **72**, 84-101.
[4] W. Liu, X. Li, J. Chen, T. Li, M. Dong and X. Lei, *Chem. Eur. J.,* 2015, **21**, 5345-5349.
[5] K. Mitsuhashi, R. Ito, T. Arai and A. Yanagisawa, *Org. Lett.,* 2006, **8**, 1721-1724.
[6] W. R. Vaughan and R. Perry Jr., *J. Am. Chem. Soc.,* 1952, **74**, 5355-5356.
[7] T. Kitahara, M. Mori and K. Mori, *Tetrahedron,* 1987, **43**, 2689-2699.
[8] A. T. Rowland, *Steroids,* 1975, **26**, 251-254.
[9] R. Dodson and B. Riegel, *J. Org. Chem*., 1948, **13**, 424-437.
[10] C. F. D. Amigo, I. G. Collado, J. R. Hanson, R. Hernádez-Galán, P. B. Hitchcock, A. J. Macias-Sanchez and D. J. Mobbs, *J. Org. Chem.,* 2001, **66**, 4327-4332.
[11] S. Shankar and R. M. Coates, *J. Org. Chem.,* 1998, **63**, 9177-9182.
[12] S. F. R. Hinkley, N. B. Perry and R. T. Weavers, *Tetrahedron*, 1997, **53**, 7035-7044.
[13] T. C. Wilde, G. Blotny and R. M. Pollack, *J. Am. Chem. Soc.,* 2008, **130**, 6577-6585.
[14] X.-K. Liu, B.-J. Ye, Y. Wu, J.-X. Nan, Z.-H. Lin and H.-R. Piao, *Chem. Biol. Drug. Des.,* 2012, **79**, 523-529.
[15] Y. Zheng and Y. Li, *J. Org. Chem*., 2003, **68**, 1603-1606.
[16] D. N. Kirk, W. Klyne, C. M. Peach and M. A. Wilson, *J. Chem. Soc. C,* 1970, 1454-1460.
[17] D. H. R. Barton, D. J. Lester and S. V. Ley, *J. Chem. Soc., Perkin Trans. 1*, 1980, 2209-2212.
[18] K. Ohgane, F. Karaki, T. Noguch-Yachide, K. Dodo and Y. Hashimoto, *Bioorg. Med. Chem. Lett.,* 2014, **24**, 3480-3485.
[19] Z.-X. Jiang, J.-Q. Ye, L. Jiang and Y.-S. Zhao, *Steroids*, 2005, **70**, 690-693.
[20] W.-S. Zhou, Z.-Q. Wang and B. Jiang, *J. Chem. Soc., Perkin Trans. 1,* 1990, 1-3.
[21] R. Csuk, S. Shwarz, R. Kluge and D. Ströhl, *Eur. J. Med. Chem.,* 2010, **45**, 5718-5723.
[22] J.-B. Xia, C. Zhu and C. Chen, *Chem. Commun.,* 2014, **50**, 11701-11704.
[23] G. M. Sheldrick, *Acta Cryst.*, 2015, **C71**, 3-8.
